# Supplementary material for: Patient safety climate in general public hospitals in China: differences associated with department and job type based on a cross-sectional survey
Source: BMJ Open. 2018 Apr 17;8(4):e015604. doi: 10.1136/bmjopen-2016-015604 (PMC5905765; doi:10.1136/bmjopen-2016-015604)
Supplement: Supplementary file 1 [file bmjopen-2016-015604supp001.pdf]

Appendix A: General Respondent Information

Work Area Characteristics	N	%
Region		
Shanghai	2094	50.81
Hubei Province	1095	26.57
Gansu Province	932	22.62
Total	4121	100.00
Hospital level		
Tertiary	1827	44.33
Secondary	2294	55.67
Total	4121	100.00
Hospital size (beds)		
Small (≤500)	841	20.41
Middle (501-800)	1131	27.44
Large (>800)	2149	52.15
Total	4121	100.00
Affiliated with medical school		
Yes	2644	65.08
No	1419	34.92
Total	4063	100.00
Clinical department		
Internal Medicine	791	19.46
Surgery	772	18.99
Obstetrics & Gynecology	266	6.54
Pediatrics	128	3.15
ICU	146	3.59
Emergency Department (ED)	247	6.08
Anesthesiology	63	1.55
Operating Room (OR)	90	2.21
Clinical Auxiliary Departments (CADs)†	549	13.51
Others△	1013	24.92
Total	4065	100.00
Doctor-nurse ratio		
Mean (SD)	0.81	0.59
Bed-Nurse ratio		
Mean (SD)	2.53	2.52

Worker Characteristics	N	%
Gender		
Male	1365	33.69
Female	2687	66.31
Total	4052	100.00
Age		
<25	269	6.67
25-34	771	19.11
35-45	879	21.79
>=45	2115	52.43
Total	4034	100.00
Education		
Master's degree or above	737	18.03
Bachelor's degree	2205	53.94
Associate's degree or below	1146	28.03
Total	4088	100.00
Working years		
<5	1073	26.63
5-10	852	21.15
>10	2104	52.22
Total	4029	100.00
Monthly income (RMB)		
<4000	1311	32.25
4000-7999.99	1767	43.47
≥8000	987	24.28
Total	4065	100.00
Job type		
Frontline physicians	1563	38.66
(Associate) Chief physicians	*417*	*10.32*
Attending physicians	*628*	*15.53*
Resident or below	*518*	*12.81*
Frontline nurses	1480	36.61
Medical technicians	375	9.28
Managers‡	116	2.87
Non-professionals and non-managers	509	12.58
Total	4043	100.00

† Refers to laboratory department, image department, ECG department, pathology department, pharmacy department, supply center, etc.

△ Including stomatology, dermatology, ENT, ophthalmology, psychiatry, traditional Chinese medicine departments, and administrative offices.

‡ Including managers in the clinical departments, clinical auxiliary departments, and administrative departments.

Appendix B: Differences in Patient Safety Climate among Job Types (Pair-wise Comparison with Physicians) †

Dimensions	Nurses-Physicians		Medical Technicians-Physicians		Managers-Physicians		Others-Physicians	
	Estimate	95% CL	Estimate	95% CL	Estimate	95% CL	Estimate	95% CL
Hospital contributions to the safety climate								
Senior managers' engagement	-1.83	[-3.29,-0.38]	-1.24	[-3.71,1.23]	-1.62	[-4.82,1.58]	-2.25	[-3.96,-0.53]
Organizational resources for safety	-2.28	[-5.34,0.77]	-3.39	[-8.56,1.78]	-6.55	[-13.24,-0.15]	-5.30	[-8.89,-1.70]
Overall emphasis on patient safety	-0.98	[-2.78,0.81]	-1.53	[-4.59,1.53]	-0.24	[-4.20,3.72]	-1.60	[-3.71,0.51]
Work-unit contributions to the safety climate								
Unit managers' support	-0.65	[-2.95,1.65]	-1.89	[-5.80,2.02]	-1.76	[-6.83,3.30]	-2.90	[-5.6,-0.19]
Unit safety norms	0.07	[-1.43,1.56]	-2.00	[-4.53,0.53]	-1.57	[-4.85,1.71]	-0.36	[-2.12,1.39]
Unit recognition and support for safety efforts	-4.32	[-6.75,-1.90]	-3.59	[-7.68,0.51]	-5.08	[-10.39,-0.23]	-5.30	[-8.16,-2.44]
Collective learning	-1.06	[-2.38,0.26]	-2.11	[-4.35,0.13]	-1.13	[-4.04,1.77]	-0.36	[-1.92,1.19]
Psychological safety	-2.46	[-4.89,-0.04]	-4.40	[-8.51,-0.29]	-8.71	[-14.03,-3.39]	-3.04	[-5.89,-0.19]
Problem responsiveness	-2.46	[-4.19,-0.73]	-3.99	[-6.92,-1.07]	-5.41	[-9.20,-1.62]	-2.48	[-4.51,-0.45]
Interpersonal contributions to the safety climate								
Fear of shame	1.37	[-3.18,5.93]	-0.90	[-8.59,6.78]	0.57	[-9.39,10.52]	-0.40	[-5.78,4.97]
Fear of blame and punishment	-1.98	[-7.30,3.34]	-2.38	[-11.34,6.59]	1.35	[-10.26,12.96]	2.88	[-3.41,9.16]
Other aspects of the safety climate								
Provision of safe care	3.50	[-0.87,7.87]	1.42	[-5.96,8.80]	3.11	[-6.45,12.66]	0.94	[-4.22,6.09]
Overall	-1.36	[-2.53,-0.18]	-2.29	[-4.28,-0.3]	-2.74	[-5.32,-0.16]	-1.97	[-3.36,-0.59]

† Results reflect differences in predicted values (estimate and 95% 95% CL) among job types by pair-wise comparison with physicians based on 2-level random intercept models for each dimension and the overall safety climate adjusted for other individual characteristics (age, gender, education, working years, and monthly income) and hospital characteristics (tertiary level vs secondary level, hospital size, hospital location, and doctor-nurse ratio). When estimating the predicted mean values of the PPR for each dimension and overall by clinical department and job type, we held other covariates constant at their means.

Appendix C: Differences in Patient Safety Climate among Job Types (Pair-wise Comparison with Managers) †

Dimensions	Physicians-Managers		Nurses-Managers		Medical Technicians -Managers		Others-Managers	
	Estimate	95% CL	Estimate	95% CL	Estimate	95% CL	Estimate	95% CL
Hospital contributions to the safety climate								
Senior managers' engagement	1.62	[-1.38,4.62]	-0.21	[-3.36,2.94]	0.38	[-2.47,3.23]	-0.63	[-3.74,2.49]
Organizational resources for safety	6.55	[0.28,12.82]	4.27	[-2.33,10.86]	3.16	[-2.81,9.12]	1.25	[-5.27,7.78]
Overall emphasis on patient safety	0.24	[-3.47,3.94]	-0.75	[-4.64,3.15]	-1.29	[-4.82,2.23]	-1.36	[-5.21,2.00]
Work-unit contributions to the safety climate								
Unit managers' support	1.76	[-2.98,6.51]	1.11	[-3.87,6.10]	-0.13	[-4.64,4.38]	-1.13	[-6.07,3.80]
Unit safety norms	1.57	[-1.50,4.64]	1.64	[-1.60,4.87]	-0.43	[-3.35,2.49]	1.21	[-1.99,4.40]
Unit recognition and support for safety efforts	5.08	[0.11,10.05]	0.76	[-4.47,5.99]	1.50	[-3.23,6.23]	-0.22	[-5.39,4.96]
Collective learning	1.13	[-1.58,3.85]	0.07	[-2.78,2.93]	-0.97	[-3.56,1.61]	0.77	[-2.05,3.60]
Psychological safety	8.71	[3.73,13.70]	6.25	[1.01,11.49]	4.31	[-0.43,9.06]	5.67	[0.48,10.85]
Problem responsiveness	5.41	[1.86,8.96]	2.95	[-0.78,6.68]	1.42	[-1.96,4.79]	2.93	[-0.76,6.62]
Interpersonal contributions to the safety climate								
Fear of shame	-0.57	[-9.89,8.76]	0.81	[-9.00,10.62]	-1.47	[-10.34,7.41]	-0.97	[-10.68,8.74]
Fear of blame and punishment	-1.35	[-12.23,9.52]	-3.33	[-14.78,8.11]	-3.73	[-14.08,6.63]	1.52	[-9.81,12.85]
Other aspects of the safety climate								
Provision of safe care	-3.11	[-12.05,5.84]	0.39	[-9.02,9.81]	-1.69	[-10.21,6.83]	-2.17	[-11.49,7.15]
Overall	2.74	[0.32,5.15]	1.38	[-1.16,3.92]	0.44	[-1.85,2.74]	0.76	[-1.75,3.28]

† Results reflect differences in predicted values (estimate and 95% 95% CL) among job types by pair-wise comparison with managers based on 2-level random intercept models for each dimension and the overall safety climate adjusted for other individual characteristics (age, gender, education, working years, and monthly income) and hospital characteristics (tertiary level vs secondary level, hospital size, hospital location, and doctor-nurse ratio). When estimating the predicted mean values of the PPR for each dimension and overall by clinical department and job type, we held other covariates constant at their means.
