# Supplementary material for: Patient safety climate in general public hospitals in China: differences associated with department and job type based on a cross-sectional survey
Source: BMJ Open. 2018 Apr 17;8(4):e015604. doi: 10.1136/bmjopen-2016-015604 (PMC5905765; doi:10.1136/bmjopen-2016-015604)
Supplement: Author's manuscript [file bmjopen-2016-015604.draft_revisions.pdf]

BMJ Open is committed to open peer review. As part of this commitment we make the peer review history of every article we publish publicly available.

When an article is published we post the peer reviewers' comments and the authors' responses online. We also post the versions of the paper that were used during peer review. These are the versions that the peer review comments apply to.

The versions of the paper that follow are the versions that were submitted during the peer review process. They are not the versions of record or the final published versions. They should not be cited or distributed as the published version of this manuscript.

BMJ Open is an open access journal and the full, final, typeset and author-corrected version of record of the manuscript is available on our site with no access controls, subscription charges or pay-per-view fees (http://bmjopen.bmj.com).

If you have any questions on BMJ Open's open peer review process please email info.bmjopen@bmj.com

BMJ Open

Patient Safety Climate in General Public Hospitals in China: Multilevel Modeling of Differences Associated with Department and Job Type

Journal:	*BMJ Open*
Manuscript ID	bmjopen-2016-015604
Article Type:	Research
Date Submitted by the Author:	19-Dec-2016
Complete List of Authors:	Zhou, Ping; Fudan University, School of Public Health<br>Bai, Fei; National center for medical service administration<br>Tang, Huiqing; Health and Family Planning Commission of Hubei Province<br>Bai, Jie; Fudan University School of Public Health<br>Li, Minqi; Fudan University School of Public Health<br>Xue, Di; Fudan University School of Public Health
**Primary Subject Heading**:	Health services research
Secondary Subject Heading:	Medical management, Patient-centred medicine
Keywords:	patient safety climate, working department, job type, China, Public hospital

SCHOLARONE™
Manuscripts

For peer review only

Patient Safety Climate in General Public Hospitals in China: Multilevel Modeling of Differences Associated with Department and Job Type

Authors

Ping Zhou[1], Fei Bai[2], Huiqing Tang[3], Jie Bai[1], Minqi Li[1], Di Xue[1]

[1] Key Laboratory of Health Technology Assessment (MOH); School of Public Health, Fu Dan University, Shanghai 200032, P.R. China

[2] National center for medical service administration, Beijing,100044, P.R. China

[3] Health and Family Planning Commission of Hubei Province, Wuhan, 430071, P.R. China

Corresponding author:

Di Xue

Add.: P.O.Box 197, 138 Yi Xue-yuan Rd., Shanghai, 200032, P.R.China

Email: xuedi@shmu.edu.cn

Fax: 86-21-33565181

Word count for the main text of the manuscript: 3575

For peer review only - http://bmjopen.bmj.com/site/about/guidelines.xhtml

Abstract (192 words)

Objective: This study aimed to analyze the differences in the perception of patient safety climate among different working departments and job types in general public hospitals in China.

Design: Cross-sectional survey.

Setting: 18 tertiary hospitals and 36 secondary hospitals from 10 areas in Shanghai, Hubei Province, and Gansu Province, China.

Participants: In total, 4,176 staff including physicians, nurses, medical technicians, managers were recruited during March to June of 2015.

Main outcome measure: The Patient Safety Climate in Health Care Organizations (PSCHO) tool and the percentages of "problematic responses" (PPRs) were used to measure. Multivariate hierarchical linear models were applied in the analysis.

Results: In the study, 4,121 valid questionnaires were collected. The perceptions of the patient safety climate varied among departments and job types. Physicians responded with more negative evaluations of organizational resources for safety, unit recognition and support for safety efforts, psychological safety, problem responsiveness and overall climate than other staff. Department differences across all the dimensions and the overall safety climate mainly depended on job types.

Conclusions: The differences suggested that strategies and measures for the improvement of patient safety climate should be tailored for working departments and job types.

Keywords

patient safety climate, working departments, job type, public hospital, China

For peer review only - http://bmjopen.bmj.com/site/about/guidelines.xhtml

Strengths and Limitations of this study

- This study was carried out in Shanghai, Hubei Province, and Gansu Province, which represented high, middle, and low socioeconomic status levels located in the eastern, central and western regions of China, with a large sample of 4,176 staff from public general hospitals.
- This study was first to investigate the variation in the perception of patient safety climates among different departments and job types and the interaction of them in the public general hospitals of China.
- We used hierarchical linear models (HLMs) to examine the relationships between work departments and job types with the patient safety climate survey responses.
- To depict differences by job type within selected clinical departments, we added the interaction of the clinical department and job type variables with the model and graphically displayed the relevant predictions.
- Although our analyses represented an important advance over prior studies because we adjusted for important known individual and hospital characteristics, other characteristics that were not measured could play some role in distinguishing personnel by working department and job type.
- The results from 54 public general hospitals in three regions might not be generalizable to all hospitals in China, although our sample size was large and represented public hospitals in high, middle, and low socioeconomic level regions and the eastern, central, and western regions of China.

For peer review only

For peer review only - http://bmjopen.bmj.com/site/about/guidelines.xhtml

Introduction

Patient safety is a core issue in health care services. The Chinese government and hospitals have made great efforts to strengthen patient safety climate and to improve patient safety performance.[1-4]

Because patient safety climate is associated with positive outcomes, such as greater error reporting,[5] less adverse events,[6,7] lower mortality rates,[8] and lower readmission rates.[9] Measuring patient safety climate and understanding its variations can be helpful in targeting efforts to improve patient safety.[10-12] However, the climate can vary within organizations in different ways. Previous studies have indicated that patient safety climate of particular departments varies both across and within institutions.[13-17] Therefore, neglecting the patient safety climate at the unit level will mask important local variations, and measuring the patient safety climate of departments can identify important opportunities for improvement.

Previous literature has suggested that variations in patient safety climate may be related to the pace and complexity of the work performed in different work areas.[15,16] Most existing studies investigating the unit-specific climate have focused on measuring the climate in one or several particular types of departments with higher levels of intrinsic risk, such as the operating room (OR), intensive care unit (ICU), and/or emergency department (ED).[13-16,18] However, research concerning patient safety climates of other units, such as the pediatric, internal medicine, surgery, and medical technical departments, is deficient.

Some studies have measured the perception of patient safety climate among personnel by job type.[14,19-22] In some studies, physicians demonstrated more positive perceptions of patient safety climate than nurses and other clinical personnel.[16,18,21] However, in our previous study conducted in the hospitals of Pudong New Area,

For peer review only - http://bmjopen.bmj.com/site/about/guidelines.xhtml

Shanghai (one of the municipalities in China), we found that the physicians responded with more negative perceptions of patient safety climate than nurses.[22]

With the variation in the perception of patient safety climates among different departments and job types, the improvement efforts of patient safety climate should not be limited to the interventions at hospital level, but at department level and among different types of employees. Currently, limited articles have focused on patient safety climates at department level and among different types of employees in hospitals in China.

This study aimed to analyze the differences in the perception of patient safety climate among different working departments and job types in 54 public hospitals located in the eastern, central and western regions of China. We selected not only intrinsically hazardous departments but also others, including the internal medicine, surgery, obstetrics and gynecology, pediatric, and medical technical departments. We specifically explored: ① in which departments, the staff perceived patient safety climate more negatively, ② in which job types, the staff rated lower scores across safety climate dimensions, and ③ whether differences by job type persisted across departments.

Methods

Survey instrument

In the study, we applied the Patient Safety Climate in Healthcare Organizations (PSCHO) tool[23-25] to measure patient safety climate. We selected the PSCHO for measurement, because it demonstrated good reliability and validity[22,24,25] and captured particular underlying characteristics of Chinese culture (i.e., "fear of blame" and "fear of shame") .

The PSCHO contains 12 dimensions and three categories (based on the hospital, work unit, and interpersonal contributions to the safety climate).[23,24] The PSCHO

For peer review only - http://bmjopen.bmj.com/site/about/guidelines.xhtml

items use a five-point Likert scale ranging from ‘‘strongly disagree’’ to ‘‘strongly agree’’ with a neutral midpoint.

In this study, we added two items to the PSCHO: "Staff can freely voice their opinions on patient safety" in the dimension "Psychological Safety" and "We will analyze the accidents or unexpected events in a timely manner" in the dimension "Problem Responsiveness". The survey also asked informants to provide demographic information, including gender, age, education, working years, monthly income, working department and job type.

Sample

A cross-sectional survey was conducted using a stratified sampling method in Shanghai, Hubei Province, and Gansu Province in China from March to June of 2015. The regions were selected to capture various socioeconomic statuses and geographic distributions within China. First, we selected 3 provinces representing high, middle, and low socioeconomic status levels located in the eastern, central, and western regions of China.

Then, we selected 3 prefecture-level cities/counties (areas) in Hubei Province and Gansu Province representing high, middle, and low socioeconomic status levels within each province/municipality. In each area, two tertiary public general hospitals and four secondary public general hospitals were selected.

Because the tertiary hospitals in Shanghai were not evenly distributed among districts, 6 tertiary public general hospitals were selected in Shanghai to represent the tertiary hospitals owned by universities, the Shanghai government, or district governments. Additionally, 12 secondary public general hospitals were selected from 4 districts (A-D). Because District A was the largest district in Shanghai and comprised both urban and rural areas covering approximately 20% of the total Shanghai population, 6 secondary public general hospitals were selected in this

For peer review only

For peer review only - http://bmjopen.bmj.com/site/about/guidelines.xhtml

district. In the other 3 districts in Shanghai, 2 secondary public general hospitals were selected.

We recruited a sample of 18 tertiary hospitals and 36 secondary hospitals from 10 areas in 3 regions of China in this study.

Data sources

For each selected hospital, general data (i.e., hospital level, number of beds, number of physicians, and number of nurses) were collected, and an employee questionnaire survey was conducted.

In the employee survey, we randomly sampled 10% of the managers and administrative staff (at least 15), 10% of the frontline physicians (at least 15), nurses (at least 15) and health technicians and the staff working in the medical auxiliary departments (at least 5). The term manager is a hospital or department director (including clinical departments and administrative offices), and the administrative staff are employees working in the administrative offices related to patient safety and medical quality without managerial positions. Frontline workers are employees without management responsibilities who interact directly with patients. Internal medicine department, surgical department, obstetrics and gynecology, ICU, and ED employees were recruited in this survey.

Survey Data Analysis

Psychometric Analysis

We conducted item analyses and a confirmatory factor analysis to test the reliability and validity of the PSCHO revised for the Chinese context. The study demonstrated that eleven dimensions had high internal consistency (Cronbach's α coefficients ranging from 0.77 to 0.93), but one dimension ("Fear of blame and punishment") had a relatively lower Cronbach's α coefficient (0.66). The overall scale had a high Cronbach's α coefficient (0.96). The standardized Root Mean Square Residual

For peer review only - http://bmjopen.bmj.com/site/about/guidelines.xhtml

(SRMR) and the Root Mean Square Error of Approximation (RMSEA) of the PSCHO were 0.049 and 0.058, respectively. Bentler's Comparative Fit Index, the Bentler-Bonett Normed Fit Index and the Non-Normed Index values were all greater than 0.9. The adjusted goodness of fit (AGFI) was 0.83. However, the Goodness of Fit Index (GFI) was 0.84, which was slightly lower than the criterion for this index (GFI >0.85). Overall, the constructive validity of the PSCHO revised for the Chinese cultural context in this study was acceptable.[26,27]

Statistical Analysis

We used the percentage of "problematic responses" (PPRs) to measure the patient safety climate. A rating less than 3 for a positive statement or greater than 3 for a negative statement was identified as a problematic response. A lower PPR is indicative of a better perception of the safety climate. This scoring method identifies areas of non-uniformity in safety that are of potential concern and may benefit from interventions to improve the safety climate.[21-22,28-30]

We computed the PPR for each safety climate dimension with each item in the dimension weighted equally. We also calculated the average PPR for all questions in the survey as a summary statistic, which we referred to as the "overall safety climate". These percentages were calculated as the averages of all responses received. Comparisons among working departments and job types were calculated separately for the respondents who indicated that they worked in any of the 8 working departments (internal medicine, surgery, obstetrics and gynecology, pediatrics, ICU, ED, medical technical departments (MTD) and others) and then for employees by different job type (physicians, nurses, administrative staff, hospital or department directors and others).

We used a hierarchical linear model (HLM) to examine the relationships between work areas and worker characteristics with the patient safety climate survey responses. To account for the association between hospital membership and the

For peer review only - http://bmjopen.bmj.com/site/about/guidelines.xhtml

safety climate, which was significant both overall and for each dimension ($p<0.0001$), we included the respondents' hospital as a random factor in the form of a 2-level random intercept analysis. We estimated HLMs in which each safety climate dimension and the overall safety climate were the dependent variables and the working department and job type measures were the independent variables. All models were controlled for variables describing other respondent characteristics (i.e., gender, age, education, length of working years, and monthly income) and hospital characteristics (i.e., region, hospital level, bed size, and doctor-nurse ratio).

To indicate differences in perceptions among relative groups of personnel, we used regression coefficients to predict the PPR for each dimension and overall by working department and job type, with variables representing other individual characteristics held constant at their means and setting variables representing hospital characteristics to the mean for the participating hospitals. Thus, the predictions indicate average respondents in average hospitals for each personnel category. To depict differences by job type (focusing on physicians with various professional titles and other employees) within selected clinical departments, we added the interaction of the clinical department and job type variables with the model and graphically displayed the relevant predictions. We focused on physicians in the interaction analysis because they were the most important staff in the healthcare service and we intended to investigate whether there were differences among chief physicians, attending physicians, residents or below and other personnel within a particular department.

Results

Respondent characteristics

In this study, 4,176 staff from 54 selected hospitals responded to the survey and 98.68% of them returned valid questionnaires. Among valid respondents, 55.67% worked in secondary hospitals, 50.81% in Shanghai hospitals, 52.15% in large

For peer review only - http://bmjopen.bmj.com/site/about/guidelines.xhtml

bed-size hospitals (>800 beds). The percentages of respondents working in the internal medicine, surgery, obstetrics and gynecology, pediatric, ICU, ED, anesthesiology, OR and medical technical departments were 19.46%, 18.99%, 6.54%, 3.15%, 3.59%, 6.08%, 1.55%, 2.21% and 13.51%, respectively. According to job type, 38.66% were frontline physicians (including 10.32% associate chief physicians or chief physicians, 15.53% attending physicians, and 12.81% residents or below), 36.61% were frontline nurses, 9.28% were medical technicians and 2.87% were managers.

The respondents were predominantly female (66.31%) and older than 45 years (52.43%). Nearly 52% of the respondents had worked within their hospitals for 10 years or more (Appendix 1).

Perceptions of the Safety Climate

In this multi-region study, the mean PPR of the overall safety climate among all 54 hospitals was 9.00%. The dimension with the highest PPR was "fear of blame and punishment" (64.81%), and the dimension with the lowest PPR was collective learning (2.10%) (Table 1).

Variations among Working Departments

After controlling for the individual and hospital characteristics, the predicted PPR for the overall safety climate was the highest among the respondents working in EDs (9.63%), followed by the medical technical, pediatric and ICU departments (9.52%, 9.46% and 9.19%, respectively) and the lowest among the respondents in obstetrics and gynecology departments (6.82%) (Table 2).

The PPRs among the respondents in the EDs, ICUs, medical technical departments, and pediatric departments were also higher across the dimensions. The PPRs in the EDs were significantly higher for the senior managers' engagement, unit recognition and support for safety efforts and collective learning. The PPRs in the ICUs were

For peer review only - http://bmjopen.bmj.com/site/about/guidelines.xhtml

notably higher for the senior managers' engagement and psychological safety but relatively lower for fear of blame and punishment. The PPRs in the medical technical departments were considerably higher for 5 dimensions. The respondents in pediatrics responded more negatively on organizational resources for safety, collective learning and psychological safety.

Conversely, the respondents from anesthesiology and ORs and the surgery departments evaluated almost all dimensions more positively except fear of shame. Additionally, the PPRs for the obstetrics and gynecology respondents were universally lower across dimensions, especially for fear of blame and punishment (Table 2).

Variations among Job Types

After adjusting for the personnel and hospital characteristics, the PPR reported by the physicians was the highest for the overall safety climate (10.19%) and the PPR among the managers was the lowest (7.45%) (Table 3).

The PPRs of the physicians were consistently higher among the dimensions, especially for "organizational resources for safety", "unit recognition and support for safety efforts", "psychological safety", and "problem responsiveness". Additionally, the responses of the managers to the dimensions seemed to be more positive. Although no significant differences were detected among the various job types for "fear of shame", "fear of blame and punishment", and "provision of safe care", the PPR among the nurses was slightly higher for "fear of shame" (Table 3).

Variations within Working Departments among Physicians with Various Titles and Other Personnel

According to the models in which we evaluated both the working department and job type ((associate) chief physicians, attending physicians, residents or below and other personnel), working department differences across all the dimensions of safety

For peer review only - http://bmjopen.bmj.com/site/about/guidelines.xhtml

climate and overall depended on the job type (Appendix B). The PPRs reported by physicians with different titles varied significantly within departments almost for all the dimension and the overall safety climate (Figure 1).

Regarding the overall safety climate, (associate) chief physicians and residents or below in the pediatric departments responded with significantly higher PPRs (16.88% and 15.20%, respectively), attending physicians in the ICUs responded with remarkably higher PPRs (17.58%), and residents or below in the medical technical departments responded with dramatically higher PPRs than the other physicians and personnel (17.94%).

Additionally, attending physicians in the ICUs reported the highest PPRs among the compared staff regarding the senior managers' engagement, overall emphasis on patient safety, collective learning, and problem responsiveness (all above 10%), whereas (associate) chief physicians in the ICUs reported remarkably higher PPRs than the other physicians and staff for provision of safe care (68.27%). Residents or below in the EDs reported the most negative perceptions for unit safety norms and unit recognition and support for safety efforts among the respondents (12.52% and 25.88%, respectively). The PPRs of the chief and associate chief physicians in the pediatric departments were considerably higher than those of the other physicians and staff on organizational resources for safety (36.55%) and unit recognition and support for safety efforts (21.04%). The PPRs of the chief and associate chief physicians and residents or below in the pediatric departments were significantly higher than those of others for collective learning (both nearly 10%), psychological safety (both above 20%), and fear of shame (32.80% and 30.46%, respectively). Physicians in the pediatric departments universally demonstrated prominently higher PPRs for fear of blame and punishment. Attending physicians in the internal medicine departments perceived the highest PPR on organizational resources for safety. Residents or below in the medical technical departments responded most negatively on the overall emphasis on patient safety, unit safety norms, unit

For peer review only - http://bmjopen.bmj.com/site/about/guidelines.xhtml

recognition and support for safety efforts and collective learning (13.20%, 14.21%, 24.52%, and 13.77%, respectively). Chief and associate chief physicians and residents or below responded significantly more negatively on psychological safety (26.88% and 25.51%, respectively) and problem responsiveness (15.16% and 22.80%, respectively). Additionally, the chief physicians responded with notably lower PPR for fear of blame and punishment (32.26%) and with significantly higher PPR for provision of safe care (40.37%) than the other physicians and staff (Figure 1).

Furthermore, the personnel in the obstetrics and gynecology departments universally perceived more positively than that in the other departments on fear of blame and punishment, especially chief and associate chief physicians did. However, higher PPRs among respondents in the surgery departments and anesthesiology and ORs for fear of shame did not depend on various job types.

Discussion

Hospital Patient Safety Climate

The overall perception of the patient climate in this survey was relatively good (PPR=9%) But, substantial attention should be paid to the dimensions "fear of blame and punishment" (65%), "fear of shame" (20%),"provision of safe care" (16%) and "organizational resources for safety" (10%) according to the HRO theories.[31]

General Safety Climate Variations by Department and Job Type

The results highlight differences in perceptions of patient safety climate among working departments and job types. We found that personnel in the pediatric, ICU, ED, and medical technical departments perceived substantially lower levels of safety climate than the staff in other departments. The PPRs given to the safety climate by physicians were systematically higher than those by other staff in this study, while it was opposite in Western studies.[13,16,19,32] Staff in the anesthesiology and OR

For peer review only - http://bmjopen.bmj.com/site/about/guidelines.xhtml

departments and obstetrics and gynecology departments responded more positively across almost all dimensions of safety climate and overall. More resources may have been delivered and greater efforts made to overcome safety hazards in the anesthesiology department and ORs.[15,16] Furthermore, a notably higher percentage of personnel in the surgery departments problematically perceived the dimensions "fear of shame" and "fear of blame and punishment" more negatively than the other departments. Generally, surgeons have greater social identity and self-identity. However, medical errors and/or accidents occurring in surgery are usually more severe than those in other medical departments. Therefore, staff in surgery will bear more pressure and face more serious consequences.

ICUs and EDs Should Receive More Attention

Working in ICUs and EDs is associated with a higher level of risk, complexity, difficulty, faster pace and lower predictability.[15,16] These work areas are intrinsically hazardous, and personnel working in these areas are prone to encounter a high workload and pressure that can result in burnout.[33-36] Previous studies have indicated that burnout in healthcare providers may lead to a reduction in patient safety.[37-39] Nahrgang at al. generally argued[40] that the mental and physical energy levels in burnout employees lessened safe work behaviors and thus increased the likelihood of errors and work-related injuries. The results of this survey suggest that more attention should continue to be paid to ICUs and EDs.

We also found that attending physicians in the ICUs responded more negatively, especially on the dimensions senior managers' engagement, overall emphasis on patient safety, collective learning, and problem responsiveness, whereas residents in the EDs responded negatively on the dimensions unit safety norms, unit recognition and support for safety efforts. These health care workers are important personnel at the front line in the corresponding departments who perhaps experience more safety problems.[41] Hospital managers and policy makers should carefully consider their opinions.

For peer review only - http://bmjopen.bmj.com/site/about/guidelines.xhtml

Substantial Concerns in the Pediatric Departments

Based on this investigation, the personnel in pediatrics responded with relatively higher PPRs in many dimensions and regarding the overall safety climate, especially for organizational resources, which was an alarming finding. This result was consistent with the increasingly severe shortage of pediatricians in China reported in some articles and new sources.[42-44] Statistical analyses showed that the number of pediatricians in China increased by only 5,000 from 1995-2010,[45] and the current number of pediatric doctors per 1,000 kids was only 0.5, which was one-third of the ratio in the US.[46] Moreover, the issue will become more problematic after implementation of the Two-Child policy in China. Pediatricians are constantly leaving medical practice especially in the primary healthcare institutions, and a lot of new medical graduates are not willing to join.[47] In this study, chief and associate chief pediatricians were most worried about the patient climate. The healthcare human resource shortage would be very likely to impact health care quality and patient safety negatively.

Medical Technical Departments Need Attention

Another meaningful finding of our study was that the perceptions of patient safety climate among respondents in the medical technical departments were relatively worse than those in other departments regarding several dimensions of safety climate and overall, particularly among the residents in the medical technical departments. Those residents responded with significantly higher PPRs for overall emphasis on patient safety, unit safety norms, unit recognition and support for safety efforts, collective learning, psychological safety, problem responsiveness (6 of 12 dimensions), and overall safety climate than the other staff. Hospital managers might not pay sufficient attention to the medical technical departments compared with the clinical departments, especially the residents' training and motivation in these departments.

For peer review only - http://bmjopen.bmj.com/site/about/guidelines.xhtml

Limitations

First, the data were based on self-reporting, which might involve recall/report bias. Second, because this study was a cross-sectional study, we could not rule out the potential for omitted variables. Although our analyses represented an important advance over prior studies because we adjusted for important known individual and hospital characteristics, other characteristics that were not measured could play some role in distinguishing personnel by working department and job type. Third, the results from 54 public general hospitals in three regions might not be generalizable to all hospitals in China, although our sample size was reasonably large and represented public hospitals in high, middle, and low socioeconomic level regions and the eastern, central, and western regions of China.

Conclusions

Our study highlighted differences in perceptions of patient safety climate among and within working departments and job types that were not previously documented in China. The findings indicated that safety climate improvement efforts should involve greater attention to the climate in the ICU, ED, pediatric, and medical technical departments. Therefore, it is a more effective and reasonable approach to implement specific measures to improve patient safety climate according to targeted departments and personnel.

Acknowledgements

We gratefully acknowledge the significant contributions of the following members of the research project team: Hongbo Zhu, Xuefeng Wei, Jianjun Gu. The authors also acknowledge all the hospitals that provided assistance with data collection in this research project.

Contributors

Conceived and designed the study: Di Xue. Performed the investigation: Di Xue, Fei

For peer review only - http://bmjopen.bmj.com/site/about/guidelines.xhtml

Bai, Mingqi Li, Huiqing Tang, Jie Bai, Ping Zhou. Conducted statistical analyses and interpreted the data: Ping Zhou, Mingqi Li. Drafted the manuscript: Ping Zhou. Revised the manuscript: Di Xue. All authors read and approved the final manuscript.

Competing interests

The authors have declared that no competing interest exists.

Funding

This research project was supported by the National Natural Science Foundation of China, grant number 71473047.

Ethics approval

The study was approved by the Institutional Review Board of the School of Public Health, Fudan University (IRB# 2014-03-0502).

Data sharing statement

No additional data were available.

References

1. Cheng YM, Liu Y, Liu YM. [Review on research and application of adverse events reporting system in China]. *Chin Hosp Manage* 2012; 32: 40–2. (in Chinese).
2. Hu R. WHO Release of the Chinese Version of the Patient Safety Curriculum Guide: Multi-Professional Edition. *Chin Community Physician* 2012. p. 29. p. 23. (in Chinese).

For peer review only - http://bmjopen.bmj.com/site/about/guidelines.xhtml

3. Zhu J, Li L, Li Y, Shi M, Lu H, Garnick DW, *et al.* What constitutes patient safety culture in Chinese hospitals? *Int J Qual Health Care* 2012; 24: 250-7. doi: 10.1093/intqhc/mzs010.

4. [Internet] Xu SX, National Health and Family Planning Commission of PR. [Seven measures to improve medical quality and patient safety]. China Hospital CEO 25 Dec 2015. http://www.h-ceo.com/article/read/6978/6978.html (accessed 7 Aug 2016). (in Chinese).

5. Braithwaite J, Westbrook MT, Travaglia JF, Hughes C. Cultural and associated enablers of, and barriers to, adverse incident reporting. *Qual Saf Health Care* 2010; 19: 229–33. doi: 10.1136/qshc.2008.030213.

6. Singer S, Lin S, Falwell A, Gaba D, Baker L. Relationship of safety climate and safety performance in hospitals. *Health Serv Res* 2009; 44: 399–421. doi: 10.1111/j.1475-6773.2008.00918.x.

7. Mardon RE, Khanna K, Sorra J, Dyer N, Famolaro T. Exploring relationships between Hospital patient safety culture and adverse events. *J Patient Saf* 2010; 6: 226–32. doi: 10.1097/PTS.0b013e3181fd1a00.

8. Estabrooks CA, Tourangeau AE, Humphrey CK, Hesketh KL, Giovannetti P, Thomson D, *et al.* Measuring the Hospital practice environment: a Canadian context. *Res Nurs Health* 2002; 25: 256–68. doi: 10.1002/nur.10043.

9. Hansen LO, Williams MV, Singer SJ. Perceptions of hospital safety climate and incidence of readmission. *Health Serv Res* 2011; 46: 596–616. doi: 10.1111/j.1475-6773.2010.01204.x.

10. Rose JS, Thomas CS, Tersigni A, Sexton JB, Pryor D. A leadership framework for culture change in health care. *Jt Comm J Qual Patient Saf* 2006; 32: 433– 42.

11. Flin R, Burns C, Mearns K, Yule S, Robertson EM. Measuring safety climate in health care. *Qual Saf Health Care* 2006; 15: 109–15. doi: 10.1136/qshc.2005.014761.

For peer review only - http://bmjopen.bmj.com/site/about/guidelines.xhtml

12. Singer SJ, Vogus TJ. Safety climate research: taking stock and looking forward. *BMJ Qual Saf* 2013; 22: 1–4. doi: 10.1136/bmjqs-2012-001572.

13. Makary MA, Sexton JB, Freischlag JA, Holzmueller CG, Millman EA, Rowen L, *et al.* Operating room teamwork among physicians and nurses: teamwork in the eye of the beholder. *J Am Coll Surg* 2006; 202: 746 –52. doi: 10.1016/j.jamcollsurg.2006.01.017.

14. Cooper JB, Blum RH, Carroll JS, Dershwitz M, Feinstein DM, Gaba DM, *et al.* Differences in safety climate among hospital anesthesia departments and the effect of a realistic simulation-based training program. *Anesth Analg* 2008; 106: 574 –84. doi: 10.1213/01.ane.0000296462.39953.d3.

15. Huang DT, Clermont G, Sexton JB, Karlo CA, Miller RG, Weissfeld LA, *et al.* Perceptions of safety culture vary across the intensive care units of a single institution. *Crit Care Med* 2007; 35: 165–76. doi: 10.1097/01.CCM.0000251505.76026.CF.

16. Singer SJ, Gaba DM, Falwell A, Lin S, Hayes J, Baker L. Patient safety climate in 92 US hospitals: differences by work area and discipline. *Med Care* 2009; 47: 23-31. doi: 10.1097/MLR.0b013e31817e189d.

17. Paine LA, Rosenstein BJ, Sexton JB, Kent P, Holzmueller CG, Pronovost PJ. Assessing and improving safety culture throughout an academic medical centre: a prospective cohort study. *Qual Saf Health Care* 2010; 19: 547-54. doi: 10.1136/qshc.2009.039347.

18. Gehring K, Mascherek AC, Bezzola P, Schwappach DL. Safety climate in Swiss hospital units: Swiss version of the Safety Climate Survey. *J Eval Clin Practice* 2015; 21: 332–8. doi: 10.1111/jep.12326.

19. Sullivan A, Camargo C, Cleary P, Gordon J, Kaushal R, Magid D, *et al.* Do emergency physicians and nurses differently perceive safety-related factors? The National ED Safety Study [Abstract]. *Acad Emerg Med* 2007; 14 (Suppl): S210 –1. doi: 10.1197/j.aem.2007.03.1310.

For peer review only

For peer review only - http://bmjopen.bmj.com/site/about/guidelines.xhtml

20. Sexton JB, Holzmueller CG, Pronovost PJ, Thomas EJ, McFerran S, Nunes J, *et al.* Variation in caregiver perceptions of teamwork climate in labor and delivery units. *J Perinatol* 2006; 26: 463– 70. doi: 10.1038/sj.jp.7211556.

21. Thomas EJ, Sexton JB, Helmreich RL. Discrepant attitudes about teamwork among critical care nurses and physicians. *Crit Care Med* 2003; 31: 956–9. doi: 10.1097/01.CCM.0000056183.89175.76.

22. Zhou P, Bundorf MK, Gu J, He X, Xue D. Survey on patient safety climate in public hospitals in China. *BMC Health Serv Res* 2015; 15: 53. doi: 10.1186/s12913-015-0710-x.

23. Singer S, Meterko M, Baker L, Gaba D, Falwell A, Rosen A. Workforce perceptions of hospital safety culture: development and validation of the patient safety climate in healthcare organizations survey. *Health Serv Res* 2007; 42: 1999–2021. doi: 10.1111/j.1475-6773.2007.00706.x.

24. Hartmann CW, Rosen AK, Meterko M, Shokeen P, Zhao S, Singer S, *et al.* An overview of patient safety climate in the VA. *Health Serv Res* 2008; 43: 1263–84. doi: 10.1111/j.1475-6773.2008.00839.x.

25. Singer SJ, Hartmann CW, Hanchate A, Zhao S, Meterko M, Shokeen P, et al. Comparing safety climate between two populations of hospitals in the United States. *Health Serv Res* 2009; 44: 1563-84. doi: 10.1111/j.1475-6773.2009.00994.x.

26. Bentler PM, Bonett DG. Significance Tests and Goodness of Fit in the Analysis of Covariance Structures. *Psychol Bull*. 1980;88(3):588–606

27. Hu L, Bentler PM. Cutoff Criteria for Fit Indexes in Covariance Structure Analysis: Conventional Criteria versus New Alternatives. *Struct Equation Model* 1999;6(1):1–55.

28. Gaba DM, Howard SK, Jump B. Production pressure in the work environment. California anesthesiologists' attitudes and experiences. *Anaesthesiol* 1994; 81: 488–500. doi: 10.1097/00000542-199408000-00028.

29. Singer SJ, Gaba DM, Geppert JJ, Sinaiko AD, Howard SK, Park KC. The culture of safety: results of an organization-wide survey in 15 California

For peer review only - http://bmjopen.bmj.com/site/about/guidelines.xhtml

hospitals. *Qual Saf Health Care* 2003; 12: 112–8. doi: 10.1097/MLR.0b013e31817925c1.

30. Singer SJ, Falwell A, Gaba DM, Baker LC. Patient safety climate in US hospitals: variation by management Level. *Med Care* 2008; 46: 1149–56.

31. Gaba DM, Singer SJ, Sinaiko AD, Bowen JD, Ciavarelli AP. Differences in safety climate between Hospital personnel and Naval aviators. *Hum Factors* 2003; 45: 173–85. doi: 10.1518/hfes.45.2.175.27238.

32 Pronovost P, Weast B, Holzmueller C, et al. Evaluation of the culture of safety: survey of clinicians and managers in an academic medical center. *Qual Saf Health Care*. 2003;12:405– 410

33. Lin PH, Xiao HX, Lin XQ. [Survey on stress management of emergency physicians and its countermeasures in Fujian province]. *Chinese Rural Health Service Administration* 2014; 34: 503-6. (in Chinese).

34. Wang L, Liu ZF, Peng XW, Luan R. Present situation investigation and analysis of emergency departments of 20 tertiary general hospitals in Heilongjiang Province (in Chinese). *Management* 2015; 35: 68-9.

35. Sheng WJ, Zhang S, Zhang L. [Analysis on ICU healthcare providers' stress resources and its countermeasures]. *Chronic Pathematology Journal* 2010; 12: 1674-5. (in Chinese).

36. Li C, Tian XH, Feng LN, Zhang R. Study on correlation between job stress and coping styles and job burnout of ICU nurses (in Chinese). *Chinese Nurs Res* 2014; 28: 3236-7.

37. Teng CI, Shyu YI, Chiou WK, Fan HC, Lam SM. Interactive effects of nurse-experienced time pressure and burnout on patient safety: a cross-sectional survey. *Int J Nurs Stud* 2010; 47: 1442–50. doi: 10.1016/j.ijnurstu.2010.04.005.

38. West CP, Tan AD, Habermann TM, Sloan JA, Shanafelt TD. Association of resident fatigue and distress with perceived medical errors. *JAMA* 2009; 302: 1294–300. doi: 10.1001/jama.2009.1389.

For peer review only - http://bmjopen.bmj.com/site/about/guidelines.xhtml

39. Halbesleben JR, Rathert C. Linking physician burnout and patient outcomes: exploring the dyadic relationship between physicians and patients. *Health Care Manage Rev* 2008; 33: 29–39. doi: 10.1097/01.HMR.0000304493.87898.72.

40. Nahrgang JD, Morgeson FP, Hofmann DA. Safety at work: a meta-analytic investigation of the link between job demands, job resources, burnout, engagement, and safety outcomes. *J Applpsychol* 2011; 96: 71–94. doi: 10.1037/a0021484.

41. Singer SJ, Falwell A, Gaba DM, Baker LC. Patient safety climate in US hospitals: variation by management level. *Med Care* 2008; 46: 1149-56. doi: 10.1097/MLR.0b013e31817925c1.

42. Ren YQ, Lu MH, Zhang Y, Ji QY. [Issues and solutions of building a pediatrician pool in Shanghai]. *Hospital Administration Journal of Chinese PLA* 2016; 23: 228-30. (in Chinese).

43. Liu SH, Pi GH, Kang Q, Zhao J, He Y. [Current situation of pediatric in Nanchong of Sichuan province]. *Journal of North Sichuan Medical College* 2015; 30: 109-111. (in Chinese).

44. [Internet] Xin Hua News. China faces shortage of pediatricians: http://news.xinhuanet.com/english/health/2014-09/11/c_133636236.htm (accessed 15 Sept 2016).

45. [Internet] Zhao CY. China faces critical pediatrician shortage. China Daily: http://www.chinadaily.com.cn/china/2011-02/17/content_12034787.htm (accessed 10 Sept 2016).

46. [Internet] China Network Television(CNTV). China facing pediatrician shortage: http://www.china.org.cn/video/2016-01/20/content_37620967.htm. (accessed 10 Sept 2016).

47. Pan SJ, Chen JM, Bai JG. Analysis and countermeasures on the causes of pediatric human resources shortage--Take Shanxi province as an example. *Soft science of Health* 2016,30(7): 31-34. (in Chinese).

For peer review only - http://bmjopen.bmj.com/site/about/guidelines.xhtml

Tables and captions

Table 1 Problematic Responses for Dimensions and Items

Table 2 Patient Safety Climate by Working Department

Table 3 Patient Safety Climate by Job type

For peer review only

For peer review only - http://bmjopen.bmj.com/site/about/guidelines.xhtml

Table 1 Problematic Responses for Dimensions and Items

Dimensions and Items [†]	% Problematic
Senior managers' engagement	2.84
Organizational resources for safety	9.55
Overall emphasis on patient safety	2.63
Unit managers' support	5.42
Unit safety norms	4.51
Unit recognition and support for safety efforts	6.68
Collective learning	2.10
Psychological safety	5.68
Problem responsiveness	2.61
Fear of shame	20.42
Fear of blame and punishment	64.81
Provision of safe care	16.31
Overall average	9.00

† Means of all items in a dimension were averaged to the calculated dimension mean.

For peer review only

For peer review only - http://bmjopen.bmj.com/site/about/guidelines.xhtml

Table 2 Patient Safety Climate by Working Department[§] **(PPR)**

	Internal Medicine	Surgery	Obstetrics -Gynecology	Pediatrics	ICU	Emergency Department (ED)	Anesthesiology + Operating Room (OR)	Medical Technical Departments	Others
Senior mangers' engagement	2.74	2.06	1.80	2.19	4.86 **	4.62 **	1.87	1.53	1.89
Organizational resources for safety	9.38	7.54	7.02	14.41 **	10.54	8.83	6.10	7.12	7.80
Overall emphasis on patient safety	2.96	2.19	1.56	2.60	2.79	2.71	3.26	2.44	1.87
Unit managers' support	5.21	4.90	3.15	4.42	5.44	6.29	2.85	5.80	4.29
Unit safety norms	3.85	3.26	3.92	4.71	3.72	5.43	2.36	6.12 *	3.99
Unit recognition and support for safety efforts	5.95	5.51	4.29	8.57	5.10	8.33 *	3.03	8.97 *	5.34
Collective learning	2.14 *	1.40	0.91	3.17 *	2.54	2.60 *	0.44	3.20 **	0.91
Psychological safety	2.87	1.83	0.72	9.50 ***	6.69 *	5.29	2.28	8.95 ***	3.05
Problem responsiveness	0.93	0.42	-0.40	0.75	1.11	2.02	0.43	5.50 ***	0.26
Fear of shame#	19.77 *	23.51 ***	12.46	16.95	20.25	20.09	21.80 *	16.22	15.60
Fear of blame and punishment#	66.45	69.93	58.80 **	61.47	60.06	65.68	66.92	68.73	67.12
Provision of safe care	17.19	17.67	22.72	19.48	22.22	19.94	19.52	16.54	18.94
Overall	8.51	8.28	6.82	9.46	9.19	9.63 **	7.44	9.52 *	7.79

§Results reflect predictions based on 2-level hierarchical regressions for each dimension and the overall safety climate adjusted for other hospital and individual characteristics

P values indicate significant differences between the given department and other departments; * p<0.05, **p<0.01, **p<0.001.

For peer review only - http://bmjopen.bmj.com/site/about/guidelines.xhtml

Table 3 Patient Safety Climate by Job type§ **(PPR)**

	Physicians	Nurses	Medical technicians	Managers	Others
Senior mangers' engagement	4.01	2.17	2.76	2.39	1.76
Organizational resources for safety	12.25 *	9.97	8.86	5.71	6.96
Overall emphasis on patient safety	3.36	2.37	1.83	3.12	1.76
Unit managers' support	6.15	5.50	4.26	4.38	3.25
Unit safety norms	4.92	4.99	2.93	3.36	4.56
Unit recognition and support for safety efforts	9.78 *	5.45	6.19	4.70	4.48
Collective learning	2.86	1.80	0.75	1.72	2.49
Psychological safety	8.30 ***	5.84 **	3.90 *	-0.41	5.26 *
Problem responsiveness	4.09 ***	1.63	0.10	-1.32	1.61
Fear of shame#	18.39	19.76	17.48	18.95	17.99
Fear of blame and punishment#	65.04	63.07	62.67	66.40	67.92
Provision of safe care	17.57	21.07	18.98	20.67	18.50
Overall	10.19 **	8.83	7.89	7.45	8.21

§Results reflect predictions based on 2-level hierarchical regressions for each dimension and the overall safety climate adjust for other hospital and individual characteristics

P values indicate significant difference between the given job type and *Managers*; * p<0.05, **p<0.01, **p<0.001.

For peer review only

For peer review only - http://bmjopen.bmj.com/site/about/guidelines.xhtml

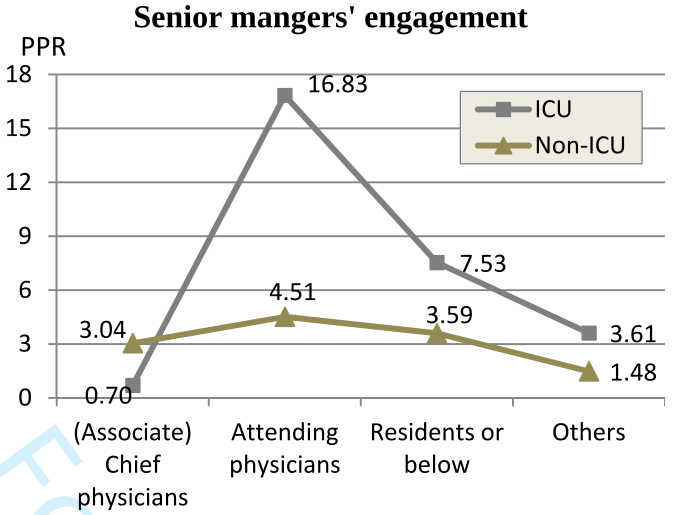
Senior mangers' engagement
PPR
18
15
12
9
6
3
0
16.83
7.53
3.61
3.04
4.51
3.59
1.48
0.70
ICU
Non-ICU
(Associate) Chief physicians
Attending physicians
Residents or below
Others

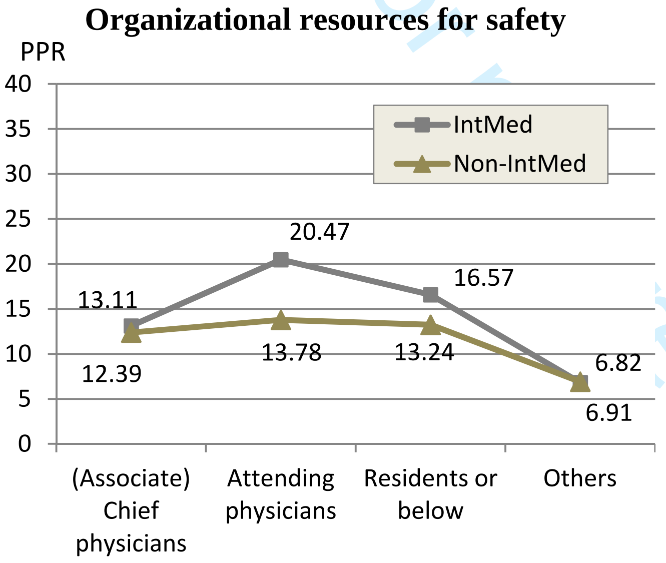
Organizational resources for safety
PPR
40
35
30
25
20
15
10
5
0
IntMed
Non-IntMed
20.47
13.11
16.57
12.39
13.78
13.24
6.82
6.91
(Associate) Chief physicians
Attending physicians
Residents or below
Others

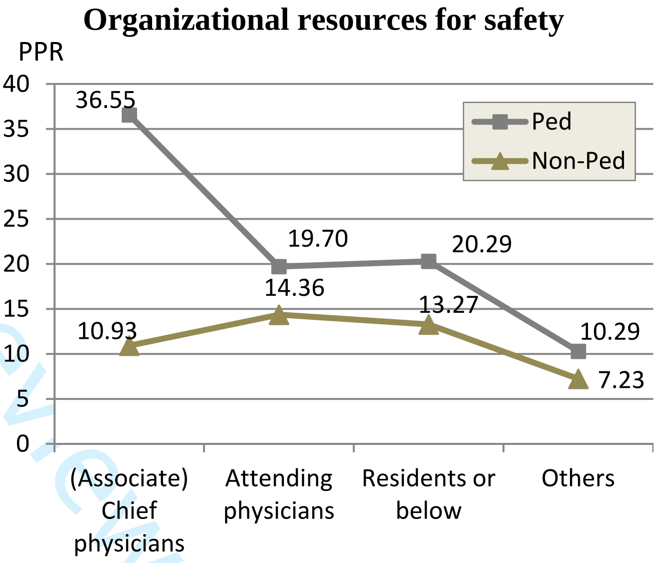
Organizational resources for safety
PPR
40
35
30
25
20
15
10
5
0
Ped
Non-Ped
36.55
19.70
20.29
14.36
13.27
10.93
10.29
7.23
(Associate) Chief physicians
Attending physicians
Residents or below
Others

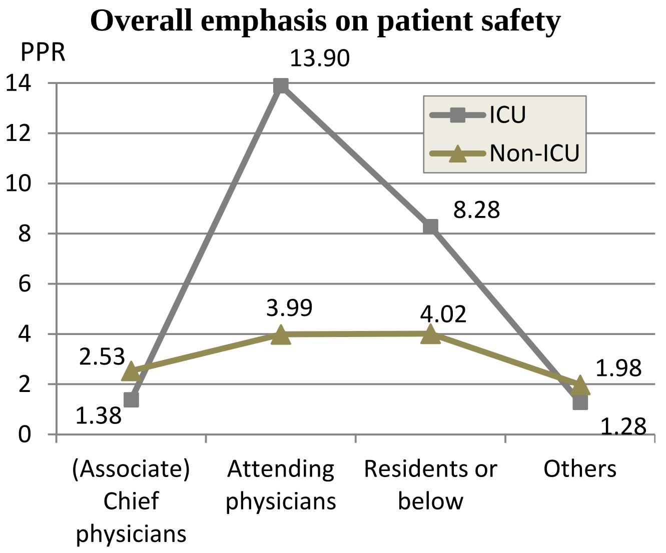
Overall emphasis on patient safety
PPR
14
12
10
8
6
4
2
0
ICU
Non-ICU
13.90
8.28
3.99
4.02
2.53
1.98
1.38
1.28
(Associate) Chief physicians
Attending physicians
Residents or below
Others

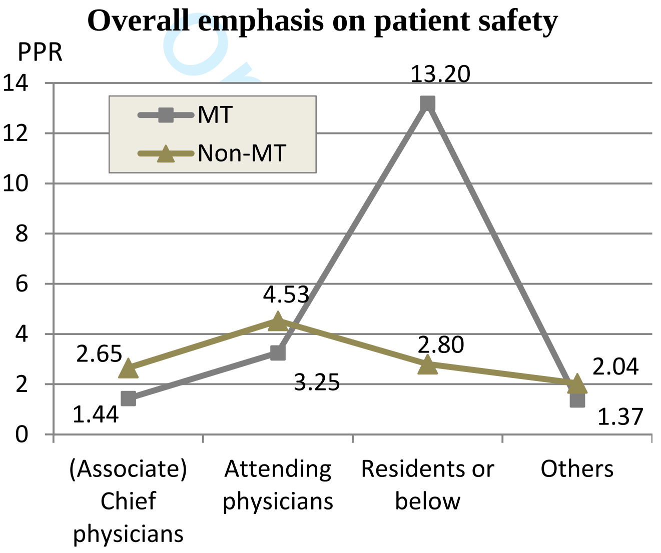
Overall emphasis on patient safety
PPR
14
12
10
8
6
4
2
0
MT
Non-MT
13.20
4.53
2.65
2.80
3.25
2.04
1.44
1.37
(Associate) Chief physicians
Attending physicians
Residents or below
Others

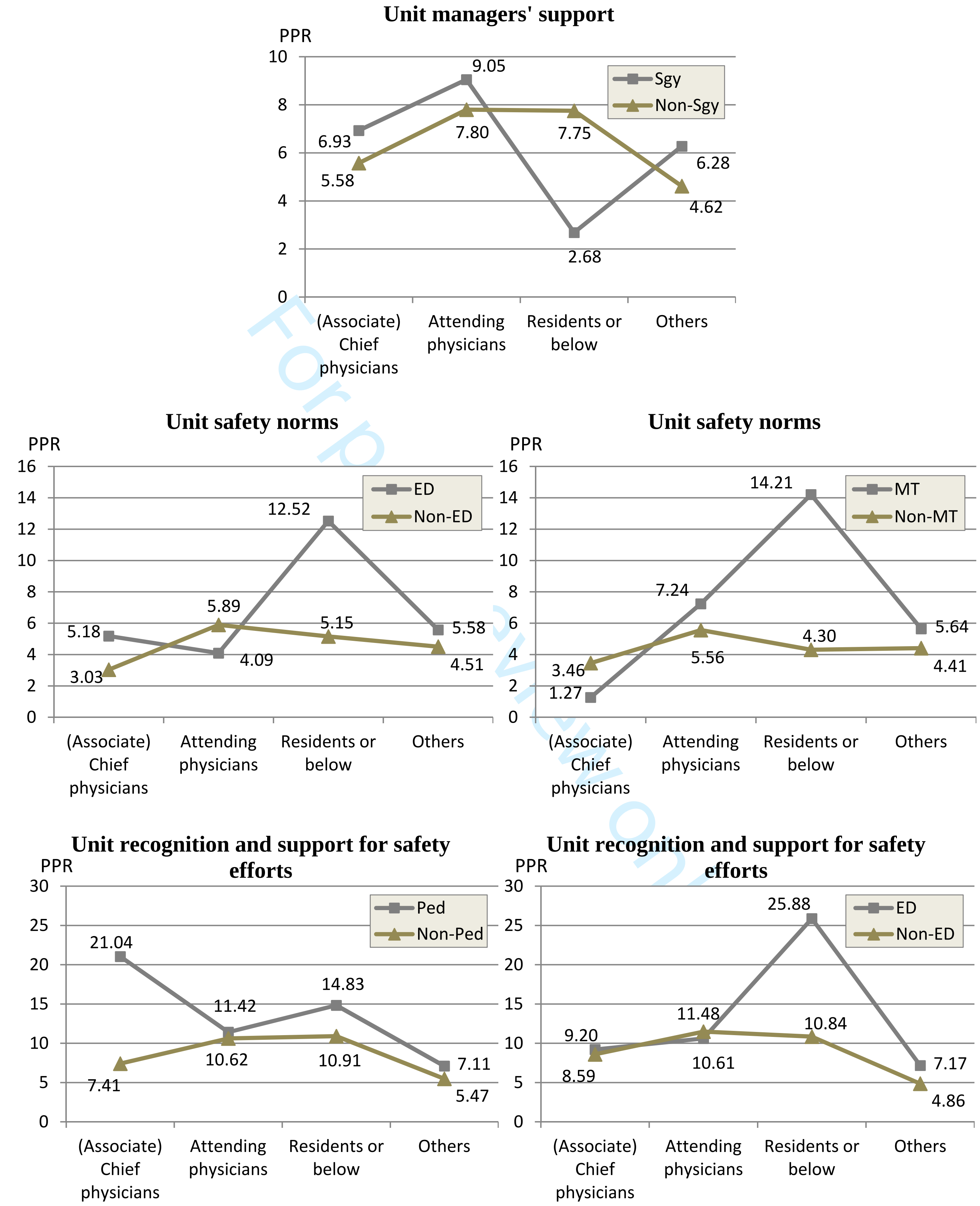

For peer review only

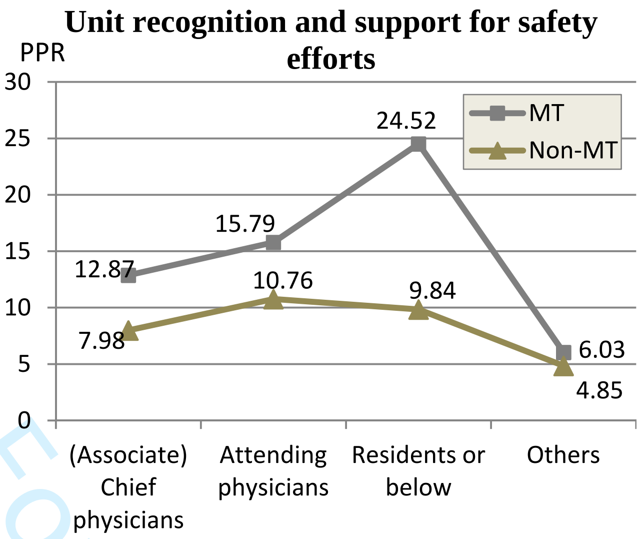

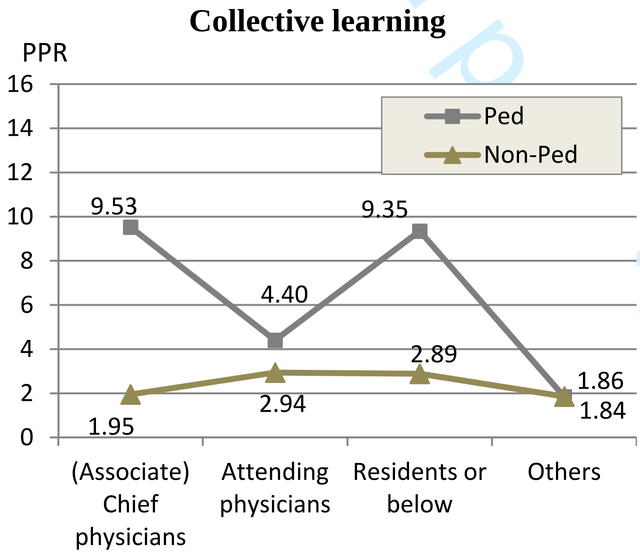

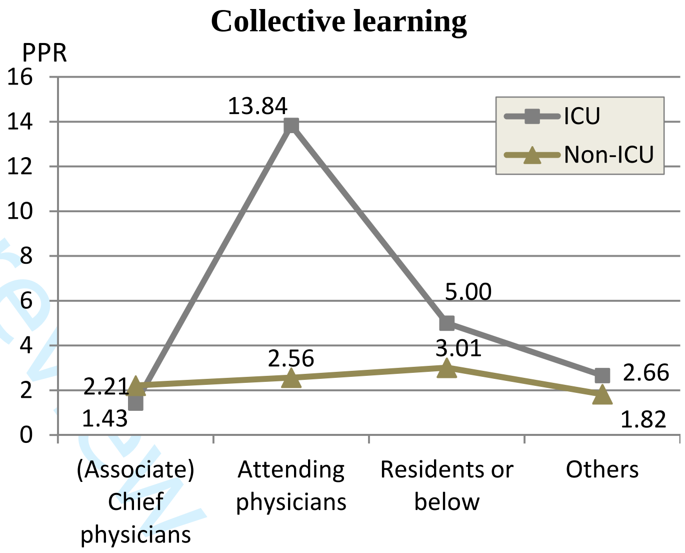

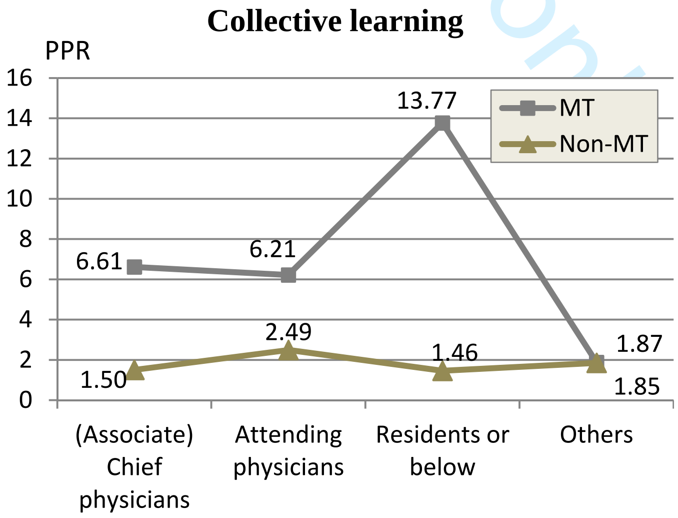

For peer review only

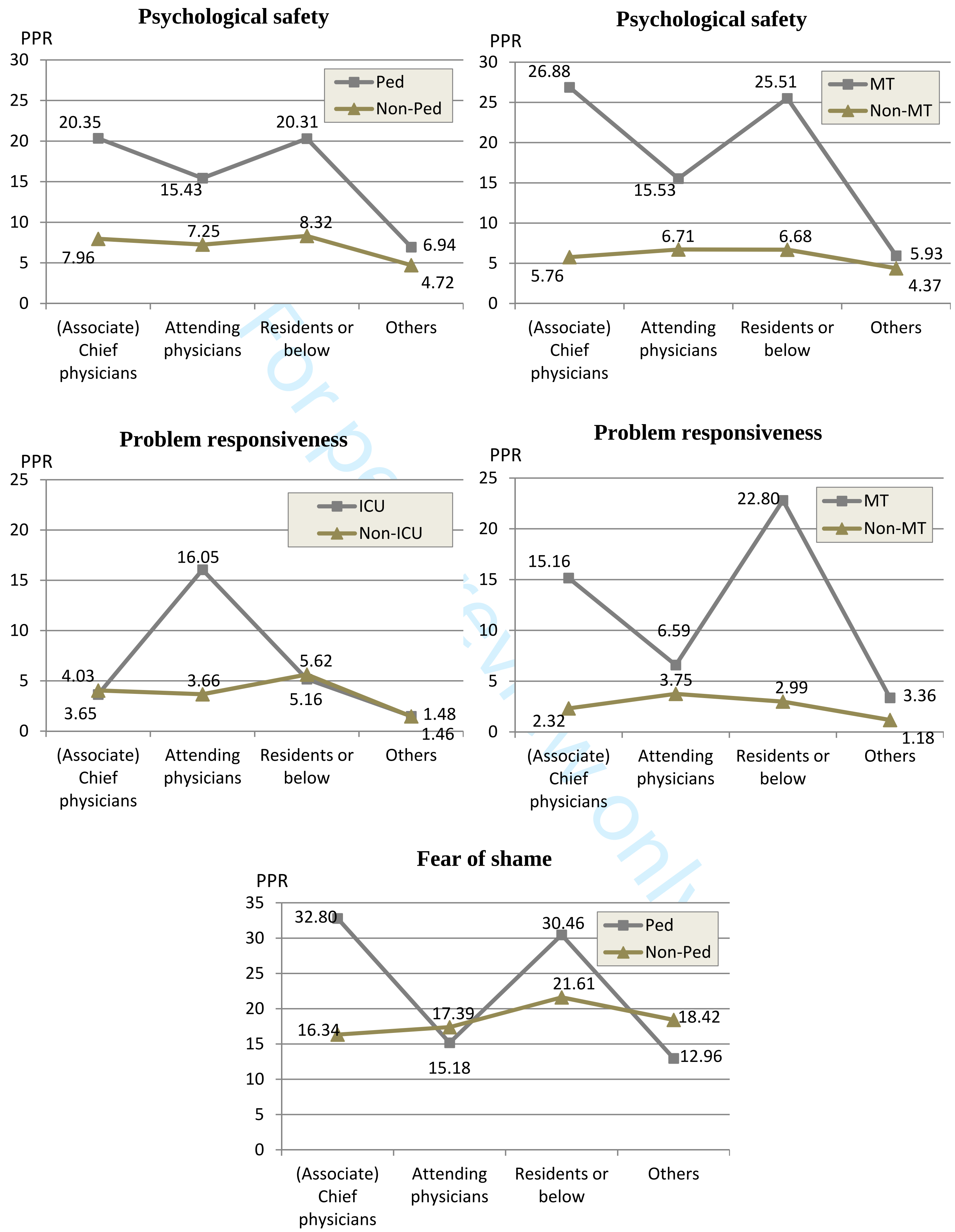
Psychological safety
PPR
Ped
Non-Ped
20.35
15.43
20.31
6.94
7.96
7.25
8.32
4.72
(Associate) Chief physicians
Attending physicians
Residents or below
Others
Psychological safety
PPR
MT
Non-MT
26.88
15.53
25.51
5.93
5.76
6.71
6.68
4.37
(Associate) Chief physicians
Attending physicians
Residents or below
Others
Problem responsiveness
PPR
ICU
Non-ICU
4.03
3.65
16.05
3.66
5.62
5.16
1.48
1.46
(Associate) Chief physicians
Attending physicians
Residents or below
Others
Problem responsiveness
PPR
MT
Non-MT
15.16
6.59
22.80
3.36
2.32
3.75
2.99
1.18
(Associate) Chief physicians
Attending physicians
Residents or below
Others
Fear of shame
PPR
Ped
Non-Ped
32.80
15.18
30.46
12.96
16.34
17.39
21.61
18.42
(Associate) Chief physicians
Attending physicians
Residents or below
Others

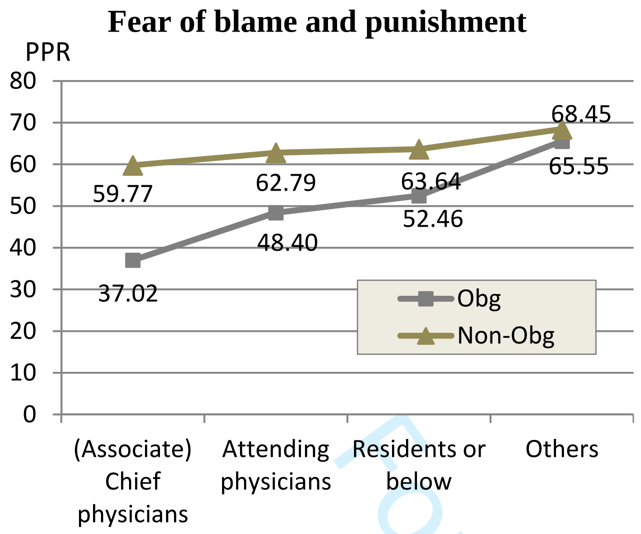
Fear of blame and punishment
PPR
80
70
60
50
40
30
20
10
0
59.77
62.79
63.64
68.45
65.55
52.46
48.40
37.02
Obg
Non-Obg
(Associate) Chief physicians
Attending physicians
Residents or below
Others

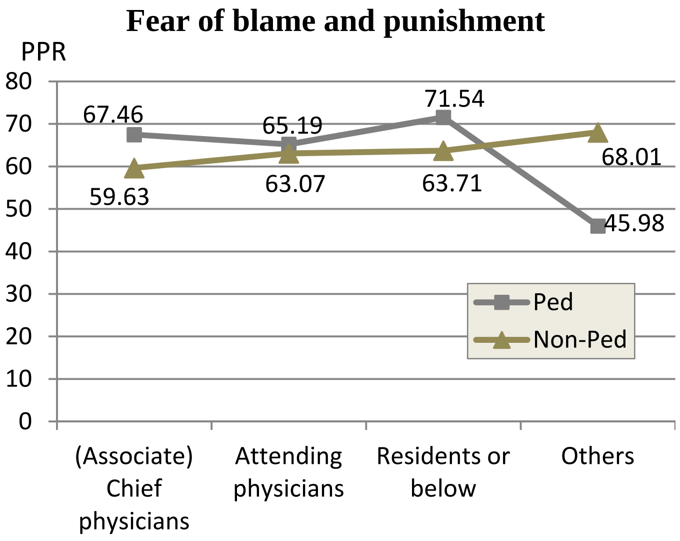
Fear of blame and punishment
PPR
80
70
60
50
40
30
20
10
0
67.46
65.19
71.54
68.01
59.63
63.07
63.71
45.98
Ped
Non-Ped
(Associate) Chief physicians
Attending physicians
Residents or below
Others

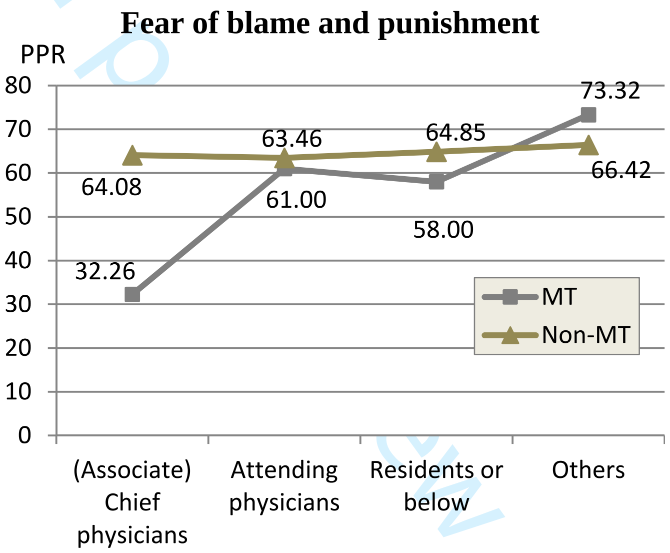
Fear of blame and punishment
PPR
80
70
60
50
40
30
20
10
0
73.32
63.46
64.85
64.08
61.00
66.42
58.00
32.26
MT
Non-MT
(Associate) Chief physicians
Attending physicians
Residents or below
Others

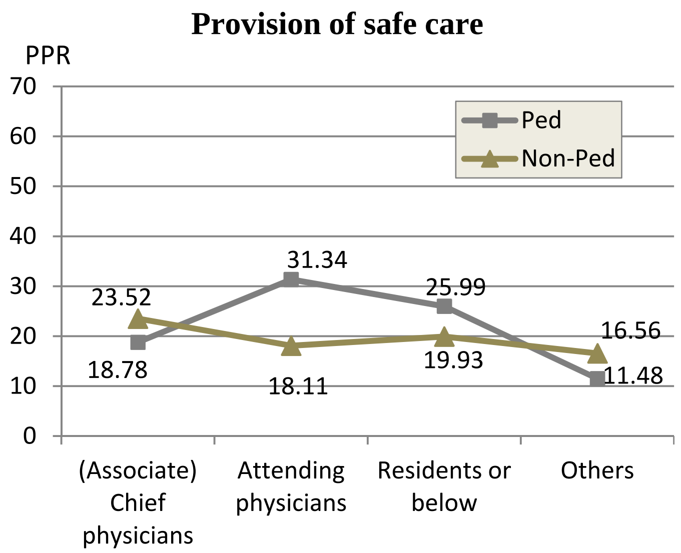
Provision of safe care
PPR
70
60
50
40
30
20
10
0
Ped
Non-Ped
31.34
25.99
23.52
16.56
18.78
18.11
19.93
11.48
(Associate) Chief physicians
Attending physicians
Residents or below
Others

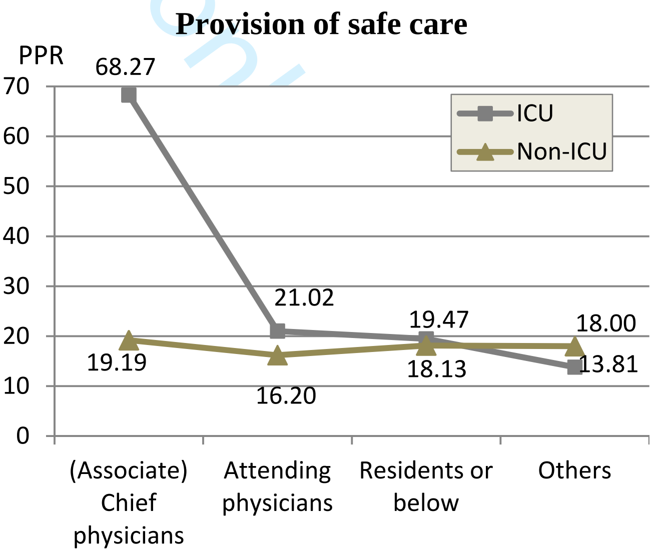
Provision of safe care
PPR
70
60
50
40
30
20
10
0
68.27
ICU
Non-ICU
21.02
19.47
18.00
19.19
18.13
13.81
16.20
(Associate) Chief physicians
Attending physicians
Residents or below
Others

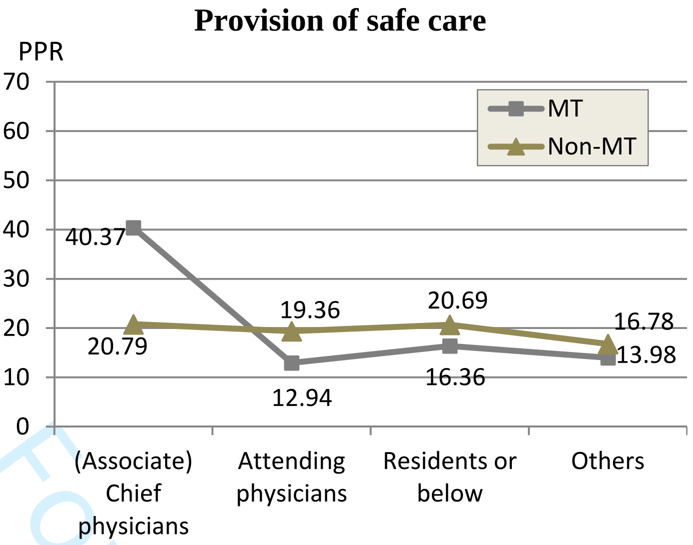

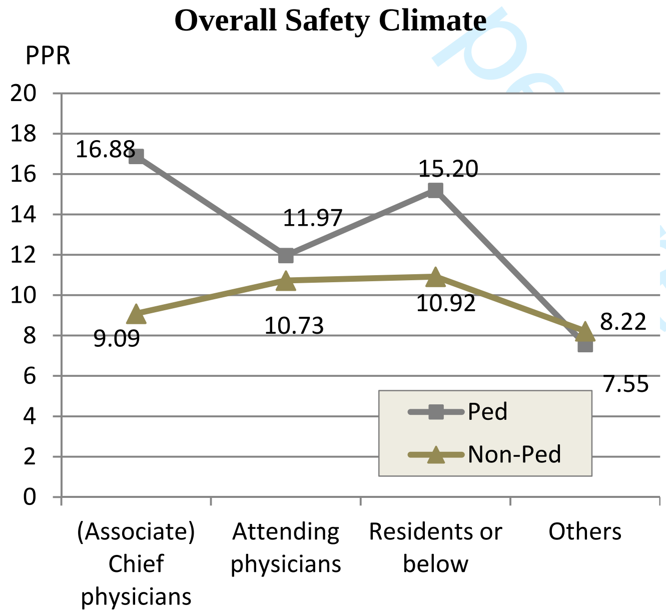

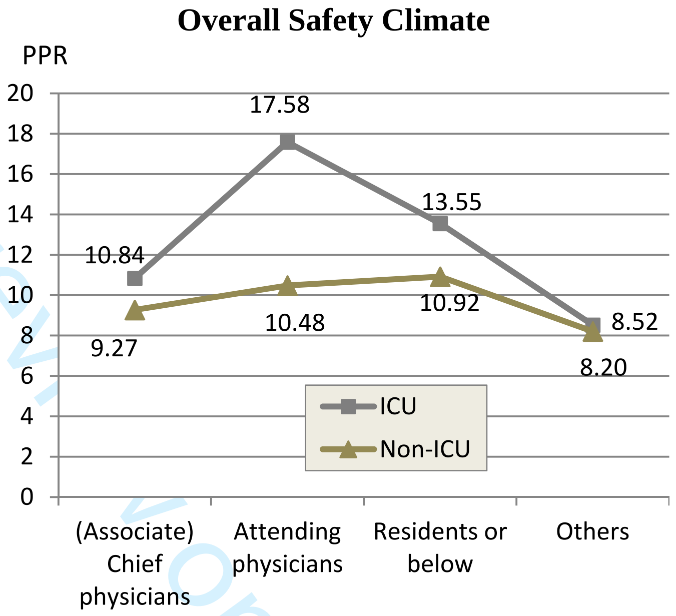

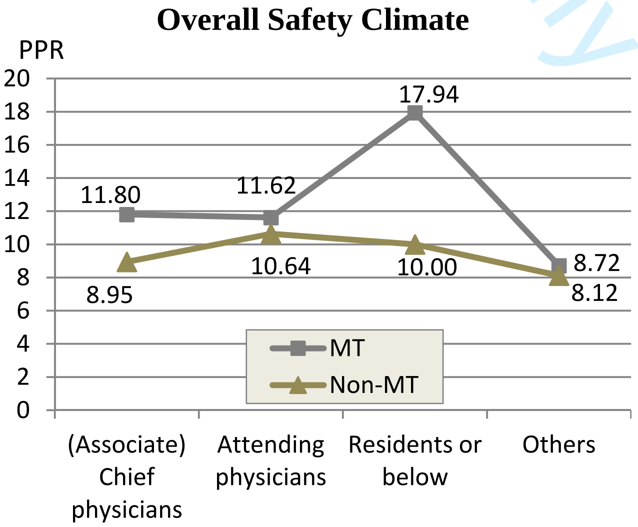

For peer review only

Figure 1 Patient safety climate by working department and job type (comparison among (associate) chief physicians, attending physicians, residents or below and others, including nurses, medical technicians, managers and other staff) overall and by the selected dimension.

Note: The results reflect predictions based on 2-level hierarchical regressions adjusted for other individual characteristics, working departments, interaction of job type * working department, and hospitals. The results of the job type*working department interaction displayed in Figure 1 were all significant ($p<0.05$).

IntMed: Internal Medicine; Sgy: Surgery; Obg: Obstetrics-Gynecology ; ICU: Intensive Care Unit; Ped: Pediatrics departments; MT: Medical Technical departments; ED: Emergency Department.

For peer review only

For peer review only

Appendix A: General Respondent Information

Work Area Characteristics	N	%
Region		
Shanghai	2094	50.81
Hubei province	1095	26.57
Gansu province	932	22.62
Total	4121	100
Hospital level		
Tertiary	1827	44.33
Secondary	2294	55.67
Total	4121	100
Bed size(beds)		
Small (⩽500)	841	20.41
Middle (501-800)	1131	27.44
Large (>800)	2149	52.15
Total	4121	100
Affiliated with Medical School		
Yes	2644	65.08
No	1419	34.92
Total	4063	100
Working Department		
Internal Medicine	791	19.46
Surgery	772	18.99
Obstetrics-Gynecology	266	6.54
Pediatrics	128	3.15
ICU	146	3.59
Emergency Department (ED)	247	6.08
Anesthesiology	63	1.55
Operating Room (OR)	90	2.21
Medical Technical Departments	549	13.51
Others△	1013	24.92
Total	4065	100
Doctor-nurse ratio		
Mean (SD)	0.81	0.59
Bed-Nurse ratio		
Mean (SD)	2.53	2.52

Worker Characteristics	N	%
Gender		
Male	1365	33.69
Female	2687	66.31
Total	4052	100
Age		
<25	269	6.67
25-34	771	19.11
35-45	879	21.79
>=45	2115	52.43
Total	4034	100
Education		
Master's degree or above	737	18.03
Bachelor's degree	2205	53.94
Associate's degree or below	1146	28.03
Total	4088	100
Working years		
<5	1073	26.63
5-10	852	21.15
>10	2104	52.22
Total	4029	100
Monthly income (RMB)		
<4000	1311	32.25
4000-7999.99	1767	43.47
⩾8000	987	24.28
Total	4065	100
Job type		
Frontline physicians	1563	38.66
(Associate) Chief physicians	*417*	*10.32*
Attending physicians	*628*	*15.53*
Resident or below	*518*	*12.81*
Frontline nurses	1480	36.61
Medical technicians	375	9.28
Managers△	116	2.87
Others¤	509	12.59
Total	4043	100

△ Including managers in the clinical, medical-technical, and administrative departments

¤ Including other staff working in medical auxiliary departments or administrative departments without managerial positions

For peer review only

Appendix B: **Summary of two-level HLM results**

Main effects only	senior mangers' engagement	organizational resources for safety	overall emphasis on patient safety	unit managers' support	unit safety norms	unit recognition and support for safety efforts	collective learning	psychological safety	problem responsiveness	fear of shame	fear of blame and punishment	provision of safe care	overall
Versus Others													
Frontline physicians	+ **	+ ***		+ **		+ ***		+ **	+ **				+ ***
Frontline nurses		+ *		+ *									
Medical technicians													
Managers								- *					
Versus all other working departments													
Internal medicine							+ *			+ *			
Surgery										+ ***			
Obstetrics-gynecology											- **		
Pediatrics		+ **					+ *	+ ***					
ICU	+ **							+ *					
Emergency Department(ED)	+ **					+ *	+ *						+ **
Anesthesiology + Operation Room(OR)										+ *			
Medical Technical Departments(MTs)					+ *	+ *	+ **	+ ***	+ ***				+ *

For peer review only

Main effects plus interactions	senior mangers' engagement	organizational resources for safety	overall emphasis on patient safety	unit managers' support	unit safety norms	unit recognition and support for safety efforts	collective learning	psychological safety	problem responsiveness	fear of shame	fear of blame and punishment	provision of safe care	overall
Versus Others													
Frontline physicians													
(Associate) Chief physicians													
Attending physicians				+ *		+ **							
Residents or below				+ *		+ *							
Frontline nurses		+ *											
Medical technicians						+ *					- *		
Managers													
Versus all other working departments													
Internal medicine													
Surgery										+ ***	+ *		
Obstetrics-gynecology													
Pediatrics											- ***		
ICU													
ED							+ *	+ *					+ *
Anesthesiology+ OR													
Medical technical departments(MTs)													

For peer review only

Main effects plus interactions	senior mangers' engagement	organizational resources for safety	overall emphasis on patient safety	unit managers' support	unit safety norms	unit recognition and support for safety efforts	collective learning	psychological safety	problem responsiveness	fear of shame	fear of blame and punishment	provision of safe care	overall
Selected interactions													
Internal medicine*(associate) chief physicians													
Internal medicine*attending physicians		+ *											
Internal medicine*residents or below													
Surgery*(associate) chief physicians													
Surgery*attending physicians													
Surgery*residents or below				- *									
Obstetrics-gynecology*(associate) chief physicians											- *		
Obstetrics-gynecology*attending physicians													
Obstetrics-gynecology*residents or below													
Pediatrics*(associate) chief physicians		+ **				+ *	+ *			+ *	+ *		+ **
Pediatrics*attending physicians											+ *	+ *	

For peer review only

Main effects plus interactions	senior mangers' engagement	organizational resources for safety	overall emphasis on patient safety	unit managers' support	unit safety norms	unit recognition and support for safety efforts	collective learning	psychological safety	problem responsiveness	fear of shame	fear of blame and punishment	provision of safe care	overall
Pediatrics*residents or below							+ *	+ *			+ **		+ *
ICU*(associate) chief physicians												+ ***	
ICU*attending physicians	+ **		+ *				+ ***		+ **				+ *
ICU*residents or below													
ED*(associate) chief physicians													
ED*attending physicians													
ED*residents or below					+ *	+ **							
(Anesthesiology+OR)*(associate) chief physicians													
(Anesthesiology+OR)*attending physicians													
(Anesthesiology+OR)*residents or below													
MTs*(associate) chief physicians								+ ***	+ *		- **	+ *	
MTs*attending physicians													
MTs*residents or below			+ **		+ **	+ **	+ ***	+ ***	+ ***				+ **

Notes:

The first type of models include independent variables: working departments and job types (frontline physicians, frontline nurses, managers, medical technicians, and others), and, in the second type of models only, interactions of different titles of physicians and various working departments. In addition, models control for

For peer review only

individuals' age; gender; education (doctor or PhD, master degree, bachelor degree); working years at institution; monthly income (<4000 RMB, 4000-7999.99 RMB, >=8000 RMB); Tertiary hospital or not; hospital size (small (<=500 beds), middle (501-800 beds), large (>800 beds)); hospital region(Shanghai, Gansu, Hubei); doctor-nurse ratio.

P-values denote significance of coefficients; * for p<0.1; ** p<0.05; ***p<0.01.

+ or - reflect direction of coefficients; only significant and marginally significant coefficients are shown.

For peer review only

STROBE Statement—checklist of items that should be included in reports of observational studies

	Item No	Recommendation	Our research
**Title and abstract**	1	(*a*) Indicate the study's design with a commonly used term in the title or the abstract	P1
		(*b*) Provide in the abstract an informative and balanced summary of what was done and what was found	P2
**Introduction**			
Background/rationale	2	Explain the scientific background and rationale for the investigation being reported	P4-5
Objectives	3	State specific objectives, including any prespecified hypotheses	P5
**Methods**			
Study design	4	Present key elements of study design early in the paper	P5-9
Setting	5	Describe the setting, locations, and relevant dates, including periods of recruitment, exposure, follow-up, and data collection	P6-7
Participants	6	(*a*) *Cohort study*—Give the eligibility criteria, and the sources and methods of selection of participants. Describe methods of follow-up	-
		Case-control study—Give the eligibility criteria, and the sources and methods of case ascertainment and control selection. Give the rationale for the choice of cases and controls	_
		Cross-sectional study—Give the eligibility criteria, and the sources and methods of selection of participants	P7
		(*b*) *Cohort study*—For matched studies, give matching criteria and number of exposed and unexposed	_
		Case-control study—For matched studies, give matching criteria and the number of controls per case	_
Variables	7	Clearly define all outcomes, exposures, predictors, potential confounders, and effect modifiers. Give diagnostic criteria, if applicable	P8-9
Data sources/ measurement	8*	For each variable of interest, give sources of data and details of methods of assessment (measurement). Describe comparability of assessment methods if there is more than one group	P5-6
Bias	9	Describe any efforts to address potential sources of bias	P9
Study size	10	Explain how the study size was arrived at	P6-7
Quantitative variables	11	Explain how quantitative variables were handled in the analyses. If applicable, describe which groupings were chosen and why	P8-9
Statistical methods	12	(*a*) Describe all statistical methods, including those used to control for confounding	P8-9
		(*b*) Describe any methods used to examine subgroups and interactions	P8-9
		(*c*) Explain how missing data were addressed	-
		(*d*) *Cohort study*—If applicable, explain how loss to follow-up was addressed	_
		Case-control study—If applicable, explain how matching of cases and controls was addressed	_
		Cross-sectional study—If applicable, describe analytical methods	P8-9

For peer review only

For peer review only - http://bmjopen.bmj.com/site/about/guidelines.xhtml

taking account of sampling strategy	
(*e*) Describe any sensitivity analyses	–

Continued on next page

For peer review only

Results			Our research
Participants	13*	(a) Report numbers of individuals at each stage of study—eg numbers potentially eligible, examined for eligibility, confirmed eligible, included in the study, completing follow-up, and analysed	-
		(b) Give reasons for non-participation at each stage	-
		(c) Consider use of a flow diagram	-
Descriptive data	14*	(a) Give characteristics of study participants (eg demographic, clinical, social) and information on exposures and potential confounders	P9-10
		(b) Indicate number of participants with missing data for each variable of interest	-
		(c) *Cohort study*—Summarise follow-up time (eg, average and total amount)	-
Outcome data	15*	*Cohort study*—Report numbers of outcome events or summary measures over time	-
		Case-control study—Report numbers in each exposure category, or summary measures of exposure	-
		Cross-sectional study—Report numbers of outcome events or summary measures	P10
Main results	16	(*a*) Give unadjusted estimates and, if applicable, confounder-adjusted estimates and their precision (eg, 95% confidence interval). Make clear which confounders were adjusted for and why they were included	P10-P13
		(*b*) Report category boundaries when continuous variables were categorized	-
		(*c*) If relevant, consider translating estimates of relative risk into absolute risk for a meaningful time period	-
Other analyses	17	Report other analyses done—eg analyses of subgroups and interactions, and sensitivity analyses	P11-13
**Discussion**			
Key results	18	Summarise key results with reference to study objectives	P13-15
Limitations	19	Discuss limitations of the study, taking into account sources of potential bias or imprecision. Discuss both direction and magnitude of any potential bias	P16
Interpretation	20	Give a cautious overall interpretation of results considering objectives, limitations, multiplicity of analyses, results from similar studies, and other relevant evidence	P13-15
Generalisability	21	Discuss the generalisability (external validity) of the study results	P16
**Other information**			
Funding	22	Give the source of funding and the role of the funders for the present study and, if applicable, for the original study on which the present article is based	P17

*Give information separately for cases and controls in case-control studies and, if applicable, for exposed and unexposed groups in cohort and cross-sectional studies.

Note: An Explanation and Elaboration article discusses each checklist item and gives methodological background and published examples of transparent reporting. The STROBE checklist is best used in conjunction with this article (freely available on the Web sites of PLoS Medicine at http://www.plosmedicine.org/, Annals of Internal Medicine at http://www.annals.org/, and Epidemiology at http://www.epidem.com/). Information on the STROBE Initiative is available at www.strobe-statement.org.

For peer review only - http://bmjopen.bmj.com/site/about/guidelines.xhtml

For peer review only

BMJ Open

Patient Safety Climate in General Public Hospitals in China: Multilevel Modeling of Differences Associated with Department and Job Type

Journal:	*BMJ Open*
Manuscript ID	bmjopen-2016-015604.R1
Article Type:	Research
Date Submitted by the Author:	28-Apr-2017
Complete List of Authors:	Zhou, Ping; Fudan University, School of Public Health<br>Bai, Fei; National center for medical service administration<br>Tang, Huiqing; Health and Family Planning Commission of Hubei Province<br>Bai, Jie; Fudan University School of Public Health<br>Li, Minqi; Fudan University School of Public Health<br>Xue, Di; Fudan University School of Public Health
**Primary Subject Heading**:	Health services research
Secondary Subject Heading:	Medical management, Patient-centred medicine
Keywords:	patient safety climate, working department, job type, China, Public hospital

SCHOLARONE™
Manuscripts

Patient Safety Climate in General Public Hospitals in China: Multilevel Modeling of Differences Associated with Department and Job Type

Authors

Ping Zhou[1], Fei Bai[2], Huiqing Tang[3], Jie Bai[1], Minqi Li[1], Di Xue[1]

[1] Key Laboratory of Health Technology Assessment (MOH); School of Public Health, Fu Dan University, Shanghai 200032, P.R. China

[2] National center for medical service administration, Beijing,100044, P.R. China

[3] Health and Family Planning Commission of Hubei Province, Wuhan, 430071, P.R. China

Corresponding author:

Di Xue

Add.: P.O.Box 197, 138 Yi Xue-yuan Rd., Shanghai, 200032, P.R.China

Email: xuedi@shmu.edu.cn

Fax: 86-21-33565181

Word count for the main text of the manuscript: 3824

For peer review only

Abstract (238 words)

Objective: This study aimed to analyze the differences in the perceived level of patient safety climate among different working departments and job types in general public hospitals in China.

Design: Cross-sectional survey.

Setting: 18 tertiary hospitals and 36 secondary hospitals from 10 areas in Shanghai, Hubei Province, and Gansu Province, China.

Participants: In total, 4,753 staff including physicians, nurses, medical technicians, managers were recruited during March to June of 2015.

Main outcome measure: The Patient Safety Climate in Health Care Organizations (PSCHO) tool and the percentages of "problematic responses" (PPRs) were used to measure. Multivariable two-level random intercept models were applied in the analysis.

Results: In the study, 4,121 valid questionnaires were collected. The perceptions of the patient safety climate varied among departments and job types. Physicians responded with more negative evaluations of organizational resources for safety, unit recognition and support for safety efforts, psychological safety, problem responsiveness and overall climate than other staff. Pediatrics, ICU and emergency departments should obtain more concerns and clinical auxiliary departments need attention. PPRs of fear of blame and punishment were prevalently significantly high and PPRs of fear of shame and provision of safe care were remarkably high especially in some departments. Besides. department differences across all the dimensions and the overall safety climate mainly depended on job types.

Conclusions: The differences suggested that strategies and measures for the improvement of patient safety climate should be tailored for working departments and job types.

Keywords

patient safety climate, working departments, job type, public hospital, China

For peer review only

For peer review only - http://bmjopen.bmj.com/site/about/guidelines.xhtml

Strengths and Limitations of this study

- This study was carried out in Shanghai Municipality, Hubei Province, and Gansu Province, which represented high, middle, and low socioeconomic status levels located in the eastern, central and western regions of China, with a large sample of 4,176 respondents from public general hospitals.
- This study was first to investigate the variation in the perceived level of patient safety climates among different departments and job types and the interaction of them in the public general hospitals of China.
- We used 2-level random intercept models to examine the relationships between work departments/job types and the patient safety climate survey responses and made results visualization by heat maps.
- To depict differences by job type within selected clinical departments, we added the interaction of the clinical department and job type variables with the model and graphically displayed the predictions by heat maps.
- Although our analyses represented an important advance over prior studies because we adjusted for important known individual and hospital characteristics, other characteristics that were not measured could play some role in distinguishing personnel by working department and job type.
- The results from 54 public general hospitals in three regions might not be generalizable to all hospitals in China, although our sample size was large and represented public hospitals in high, middle, and low socioeconomic level regions and the eastern, central, and western regions of China.

For peer review only

Introduction

Patient safety is a core issue in health care services. The Chinese government and hospitals have made great efforts to strengthen patient safety climate and to improve patient safety performance.[1-4]

Because patient safety climate is associated with positive outcomes, such as greater error reporting,[5] fewer adverse events,[6,7] lower mortality rates,[8] and lower readmission rates.[9] Measuring patient safety climate and understanding its variations can be helpful in targeting efforts to improve patient safety.[10-12] However, the climate can vary within organizations in different ways. Previous studies have indicated that patient safety climate of particular departments varies both across and within institutions.[13-17] Therefore, neglecting the patient safety climate at the unit level will mask important local variations, and measuring the patient safety climate of departments can identify important opportunities for improvement.

Previous literature has suggested that variations in patient safety climate may be related to the pace and complexity of the work performed in different work areas.[15,16] Most existing studies investigating the unit-specific climate have focused on measuring the climate in one or several particular types of departments with higher levels of intrinsic risk, such as the operating room (OR), intensive care unit (ICU), and/or emergency department (ED).[13-16,18] However, research concerning patient safety climates of other units, such as the pediatric, internal medicine, surgery, and clinical auxiliary departments, is deficient.

Some studies have measured the perception of patient safety climate among personnel by job type.[14,19-22] In some studies, physicians demonstrated more positive perceptions of patient safety climate than nurses and other clinical personnel.[16,18,21] However, in our previous study conducted in the hospitals of Pudong New Area,

For peer review only

Shanghai (one of the municipalities in China), we found that the physicians responded with more negative perceptions of patient safety climate than nurses.[22]

With the variation in the perception of patient safety climates among different departments and job types, the improvement efforts of patient safety climate should not be limited to the interventions at hospital level, but at department level and among different types of employees. Currently, limited articles have focused on patient safety climates at department level and among different types of employees in hospitals in China.

This study aimed to analyze the differences in the perception of patient safety climate among different working departments and job types in 54 public hospitals located in the eastern, central and western regions of China. We selected not only intrinsically hazardous departments but also others, including the internal medicine, surgery, obstetrics and gynecology, pediatric, and clinical auxiliary departments. We specifically explored: ① in which departments, the staff perceived patient safety climate more negatively, ② in which job types, the staff rated lower scores across safety climate dimensions, and ③ whether differences by job type within the selected clinical departments.

Methods

Survey instrument

In the study, we applied the Patient Safety Climate in Healthcare Organizations (PSCHO) tool[23-25] to measure patient safety climate. Although various instruments are available to measure a hospital's safety climate [26], we selected the PSCHO for measurement, because it demonstrated good reliability and validity[22,24,25] and captured particular underlying characteristics of Chinese culture, and use "fear of blame" and "fear of shame" to measure potential barriers to improve patient safety, [27] which captured particular underlying characteristics of Chinese culture. [22,28,29]

For peer review only

The PSCHO contains 12 dimensions and three categories (based on the hospital, work unit, and interpersonal contributions to the safety climate).[23,24] The PSCHO items use a five-point Likert scale ranging from ‘‘strongly disagree’’ to ‘‘strongly agree’’ with a neutral midpoint.

In this study, we added two items to the PSCHO: "Staff can freely voice their opinions on patient safety" in the dimension "Psychological Safety" and "We analyze accidents or unexpected events in a timely manner" in the dimension "Problem Responsiveness". These items were added because they reflect a more general psychological safety climate (not specific to certain concerns) and timely responses to adverse events in hospitals, respectively. The survey also asked informants to provide demographic information, including gender, age, education, working years, monthly income, working department and job type.

Sample

A cross-sectional survey was conducted using a stratified sampling method in Shanghai Municipality, Hubei Province, and Gansu Province in China from March to June of 2015. The regions were selected to capture various socioeconomic statuses and geographic distributions within China. First, we selected 3 provinces/municipality representing high, middle, and low socioeconomic status levels located in the eastern, central, and western regions of China.

Hubei Province has 12 prefecture-level cities and 1 autonomous prefecture. Gansu Province has 12 prefecture-level cities and 2 autonomous prefectures. We selected 3 prefecture-level cities or autonomous prefectures (areas) in Hubei Province and Gansu Province respectively to represent high, middle, and low socioeconomic status levels within each province. In each area, two tertiary public general hospitals and four secondary public general hospitals were selected.

For peer review only

Shanghai Municipality has 16 districts. Because the tertiary hospitals in Shanghai were not evenly distributed among districts, 6 tertiary public general hospitals were selected in Shanghai to represent the tertiary hospitals owned by universities, the Shanghai government, or district governments. Additionally, 12 secondary public general hospitals were selected from 4 districts (A-D) (areas). Because District A was the largest district in Shanghai and comprised both urban and rural areas covering approximately 20% of the total Shanghai population, 6 secondary public general hospitals were selected in this district. In the other 3 districts in Shanghai, 2 secondary public general hospitals were selected.

We recruited a sample of 18 tertiary hospitals and 36 secondary hospitals from 10 areas in 3 regions of China in this study.

Data sources

For each selected hospital, general data (i.e., hospital level, number of beds, number of physicians, and number of nurses) were collected, and anonymous, paper-based, self-administered employee questionnaires were distributed and collected by trained coordinators in the surveyed hospitals or regions according to our study design.

In the employee survey, we randomly sampled 10% of the managers and administrative staff (at least 15), 10% of the frontline physicians (at least 15), nurses (at least 15) and health technicians and the staff working in the medical auxiliary departments (at least 5) per hospital. The term manager is a hospital or department director (including clinical departments and administrative offices), and the administrative staff are employees working in the administrative offices related to patient safety and medical quality without managerial positions. Frontline workers are employees without management responsibilities who interact directly with patients. Employees working in internal medicine department, surgical department, obstetrics and gynecology, ICU, ED, anesthesiology and operating room (OR), clinical auxiliary departments et.al were recruited in this survey. The clinical

For peer review only

auxiliary departments are referred to laboratory department, image department, ECG department, pathology department, pharmacy department, supply center, etc

This study was approved by the Institutional Review Board of the School of Public Health, Fudan University (IRB# 2014-03-0502).

Survey Data Analysis

Psychometric Analysis

We conducted item analyses and a confirmatory factor analysis to test the reliability and validity of the PSCHO revised for the Chinese context. In the confirmatory factor analysis, we used PROC CALIS in SAS (SAS Institute Inc., Cary, NC) to form a structural equation model with the raw data. The study demonstrated that eleven dimensions had high internal consistency (Cronbach's α coefficients ranging from 0.77 to 0.93), but one dimension ("fear of blame and punishment") had a relatively lower Cronbach's α coefficient (0.66). The overall scale had a high Cronbach's α coefficient (0.96). The standardized Root Mean Square Residual (SRMR) and the Root Mean Square Error of Approximation (RMSEA) of the PSCHO were 0.049 and 0.058, respectively. And the Bentler's Comparative Fit Index, the Bentler-Bonett Normed Fit Index and the Non-Normed Index values were 0.913, 0.906, and 0.907 respectively. The adjusted goodness of fit (AGFI) was 0.83. However, the Goodness of Fit Index (GFI) was 0.84, which was slightly lower than the criterion for this index (GFI >0.85). Overall, the constructive validity of the PSCHO revised for the Chinese cultural context in this study was acceptable.[30-32]

Statistical Analysis

We used the percentage of "problematic responses" (PPRs) to measure the patient safety climate. A rating less than 3 for a positive statement or greater than 3 for a negative statement was identified as a problematic response. A lower PPR is

indicative of a better perception of the safety climate. This scoring method identifies areas of non-uniformity in safety that are of potential concern and may benefit from interventions to improve the safety climate.[21-22,33-35]

We computed the PPR for each safety climate dimension with each item in the dimension weighted equally. We also calculated the average PPR for all questions in the survey as a summary statistic, which we referred to as the "overall safety climate". These percentages were calculated as the averages of all responses received. Comparisons among working departments and job types were calculated separately for the respondents who indicated that they worked in any of the 9 types of working departments (internal medicine, surgery, obstetrics and gynecology, pediatrics, ICU, ED, anesthesiology and operating room (OR), clinical auxiliary departments (CAD) and others) and then for employees by different job type (frontline physicians, frontline nurses, medical technicians, managers and others).

Two-level random intercept model was used to examine how the working department and job types affected each dimension and the overall patient safety climate, with hospital as level-2 cluster. All models were controlled for other respondent characteristics (gender, age, education, length of working years, and monthly income) and hospital characteristics (region, hospital level, bed size, and doctor-nurse ratio). When estimating the predicted values of the PPR for each dimension and overall by working department and by job type, the mean value of all continuous covariates and the proportion of categorical covariates were used.

To test the appropriateness of using a two-level model to account for the nesting of individuals within hospitals, we first calculated and tested the intra-class correlation coefficient (ICC) of the empty model, which included no independent variables for

For peer review only

each dimension and the overall PPR..[36] The results revealed significant differences in employees' perceptions of patient safety climate among hospitals (P<0.0001).

Besides, to depict differences by job type (focusing on physicians with various professional titles and other employees) within selected clinical departments, we added the interaction of the clinical department and job type variables to the models and graphically displayed the relevant predictions by a heatmap. We focused on physicians in the interaction analysis because they were the most important staff in the healthcare service and we intended to investigate whether there were differences among chief physicians, attending physicians, residents or below and other personnel within a particular department.

Statistical analyses and graphics were generated by using SAS 9.20, Excel 2007 and R software 3.3.3..

Results

Respondent characteristics

In this study, 4,176 staff from 54 selected hospitals responded to the survey. The response rate and the valid response rate were 87.86% and 86.70% respectively. Among valid respondents, 55.67% worked in secondary hospitals, 50.81% in Shanghai hospitals, 52.15% in large bed-size hospitals (>800 beds). The percentages of respondents working in the internal medicine, surgery, obstetrics and gynecology, pediatric, ICU, ED, anesthesiology, OR and clinical auxiliary departments were 19.46%, 18.99%, 6.54%, 3.15%, 3.59%, 6.08%, 1.55%, 2.21% and 13.51%, respectively. According to job type, 38.66% were frontline physicians (including 10.32% associate chief physicians or chief physicians, 15.53% attending physicians, and 12.81% residents or below), 36.61% were frontline nurses, 9.28% were medical technicians and 2.87% were managers.

For peer review only

The respondents were predominantly female (66.31%) and older than 45 years (52.43%). Nearly 52% of the respondents had worked within their hospitals for 10 years or more (Appendix A).

Perceptions of the Safety Climate

In this multi-region study, the mean PPR of the overall safety climate among all 54 hospitals was 9.00%. The dimension with the highest PPR was "fear of blame and punishment" (64.81%), and the dimension with the lowest PPR was collective learning (2.10%) (Table 1).

Variations among Working Departments

After controlling for the hospital and the other individual characteristics, the top three predicted PPRs for the overall safety climate were in EDs (9.63%, 95%CL: 8.29%,10.98%), CAD (9.52%, 95%CL: 8.49%,10.55%), pediatric(9.46%, 95%CL: 7.70%,11.21%) and ICU departments (9.19%, 95%CL:7.56%,10.83%) and the lowest was in obstetrics and gynecology departments (6.82%, 95%CL: 5.52%,8.11%) (Figure 1, Appendix B).

Besides of universally high PPRs for "fear of blame and punishment" and "fear of shame" across 9 types of departments, PPRs for "provision of safe care" in the departments of obstetrics and gynecology, pediatrics, ICUs, EDs, and anesthesiology and ORs were higher (22.72%, 27.70%, 19.48%, 22.22%, and 19.94%, respectively) than other departments. In addition, PPRs for "organizational resources for safety" in pediatric departments and in ICUs were higher (14.41% and 10.54%, respectively), and "psychological safety" in pediatric departments was also relatively higher (9.50%) (Figure 1, Appendix B).

Variations among Job Types

After adjusting for the other personnel and hospital characteristics, the PPR reported by the physicians was the highest for the overall safety climate (10.19%, 95%CL:

For peer review only

9.52%,10.86%) and the PPR among the managers was the lowest (7.45%, 95%CL: 5.47%,9.43%) (Figure 2. Appendix C).

The PPRs of the physicians were consistently higher among the dimensions, especially for "organizational resources for safety", "unit recognition and support for safety efforts", "psychological safety", and "problem responsiveness". Additionally, the responses of the managers to the dimensions seemed to be more positive. (Figure 2, Appendix C-D).

Variations within Working Departments among Physicians with Various Titles and Other Personnel

Figure 3 displayed the variations of the predicted means of perception on each dimension and the overall patient safety climate among physicians with different titles and other personnel within each of 9 departments. It revealed that such variations in pediatrics, ICUs, EDs, and clinical auxiliary departments seemed significant across more dimensions.

Discussion

Patient Safety Climate in Hospitals

Patient safety climate is accounted as a core determinant for ensuring and promoting hospital safety in China. The Chinese Hospital Association (CHA) has promulgated annual patient safety goals since 2007, and the National Health and Family Planning Commission (previously called the Ministry of Health) set up a web-based voluntary adverse event reporting system in 2008. Additionally, many provincial governments have actively facilitated the use of clinical pathways, disease management programs, and various computer-assisted quality and safety programs and have linked government subsidies for public hospitals to the quality and safety of their medical care. [37,38] All these efforts are aimed to facilitate the improvement in patient safety climate in hospitals of China.

For peer review only

In this survey, the result revealed that the overall perception of the patient climate was relatively good (PPR=9%). But, substantial attention should be paid to the dimensions "fear of blame and punishment" (65%), "fear of shame" (20%),"provision of safe care" (16%) and "organizational resources for safety" (10%) according to the HRO theories.[39] The high prevalence of "fear of blame and punishment" and "fear of shame" may be attributed to inappropriate systems of performance assessments and rewards, hierarchical management style, bad doctor-patient relationships as well as quintessential Chinese notion of "face". Since hierarchical management style and quintessential notion of "face" are often seen in Chinese culture, our results of high PPRs in "fear of blame and punishment" and in "fear of shame" may exist in other hospitals with dominant Chinese culture.

General Safety Climate Variations by Department

The results highlighted significant differences in perceptions of patient safety climate among working departments. We found that personnel in the pediatric, ICU, ED, and clinical auxiliary departments perceived substantially lower levels of safety climate than the staff in other departments. Staff in the anesthesiology and ORs and obstetrics and gynecology departments responded more positively across almost all dimensions of safety climate and overall. More resources may have been delivered and greater efforts made to overcome safety hazards in the anesthesiology department and ORs.[15,16] Furthermore, a notably higher percentage of personnel in the surgery departments problematically perceived the dimensions "fear of shame" and "fear of blame and punishment" more negatively than the other departments. Generally, surgeons have greater social identity and self-identity. However, medical errors and/or accidents occurring in surgery are usually more severe than those in other medical departments. Therefore, staff in surgery will bear more pressure and face more serious consequences.

For peer review only - http://bmjopen.bmj.com/site/about/guidelines.xhtml

General Safety Climate Variations by Job Type

The results showed that the PPRs by physicians were systematically higher than those by other staff in this study, while it was opposite in Western studies.[13,16,19,40] Most frontline physicians are full-time employees of public hospitals in China, who hold dominant positions and close to patients during the whole process of medical services and are responsible for ensuring patient safety and quality in practice. And if adverse events or healthcare errors happen, patients are more likely to complain and blame the physicians who take charge of them. So frontline physicians might pay more attention to quality and safety measures and system, and they also could obtain more firsthand experiences or information of safety hazards.

This finding enlightened that hospital managers and unit directors should set up and continuously improve internal managerial system to stimulate frontline physicians to report or share their experiences and information on patient safety and healthcare quality, and to facilitate them to participate in related improving projects proactively. The measures to appoint frontline physicians to serve as unit quality controllers, to encourage them to lead a group of interdisciplinary members to carry out quality control circle activities, and to involve indicators on patient safety and quality improvement into individual performance assessment, etc. , which have been implemented in some hospitals in China, are worth to be generalized. [41-43]

Besides, the results revealed that the perceptions by managers were relatively consistent with frontline workers on many dimensions, which was different with many previous researches. [22,24,34,40] We thought that this finding might be extently attributed to the efforts and interventions by managers to promote speaking up and communicating down in public hospitals in China, which included activities such as safety culture-oriented simulation training, Leadship WalkRounds, efforts to engage frontline workers and managers in open discussion about safety events. [44-46] It was also possible, however, that these results were driven by the extent to that the

For peer review only

managers in this survey involved clinical departments' directors, which was different with our previous study in Shanghai 2013.[22] The clinical departments' directors in China are physicians who also provide healthcare for patients in the front line.

Substantial Concerns in the Pediatric Departments

Based on this investigation, the personnel in pediatrics responded with relatively higher PPRs in many dimensions and the overall safety climate, especially for "organizational resources for safety", which was an alarming finding. This result was consistent with the increasingly severe shortage of pediatricians in China reported in some articles and new sources.[47-49] Statistical analyses showed that the number of pediatricians in China increased by only 5,000 from 1995-2010,[50] and the current number of pediatric doctors per 1,000 kids was only 0.5, which was one-third of the ratio in the US.[51] Moreover, the issue will become more problematic after implementation of the Two-Child policy in China. Pediatricians are constantly leaving medical practice especially in the primary healthcare institutions, and a lot of new medical graduates are not willing to join.[52] In this study, chief and associate chief pediatricians were most worried about the patient climate, also particularly on organizational resources for safety. The healthcare human resource shortage would be very likely to impact health care quality and patient safety negatively.

ICUs and EDs Should Receive More Attention

Working in ICUs and EDs is associated with a higher level of risk, complexity, difficulty, faster pace and lower predictability.[15,16] These work areas are intrinsically hazardous, and personnel working in these areas are prone to encounter a high workload and pressure that can result in burnout.[53-56] Previous studies have indicated that burnout in healthcare providers may lead to a reduction in patient safety.[57-59] Nahrgang at al. generally argued [60] that the mental and physical energy levels in burnout employees lessened safe work behaviors and thus increased the likelihood of errors and work-related injuries. Flinn at al. conducted a post-call

For peer review only - http://bmjopen.bmj.com/site/about/guidelines.xhtml

assessment of junior doctors after extended work shifts (average 32.75h) showed a significant decline in cognitive functioning and clinical decision making performance. [61] Sharpe at al. indicated deteriorating performance of ICU residents during 26h of continuous wakefulness.[62] The results of this survey suggest that more attention should continue to be paid to ICUs and EDs. Facing the intrinsic characteristics of ICUs and EDs, it is significantly important and practical to design and optimize systems in such a way as to protect ICU and ED patients from preventable harm. This approach requires a balanced interdisciplinary effort directed to process characteristics and execution of several measures in parallel.[63] The recent examples including the standardization of processes [64-66], adaptation to cognitive limitations of human beings [67], optimization of working conditions [68], and the increasing use of supporting information technologies, et al. are worth learning.

We also found that attending physicians in the ICUs responded more negatively, almost all above 10% PPRs, whereas attending physicians and residents in the EDs responded negatively on the dimensions organizational resources for safety, unit safety norms, unit recognition and support for safety efforts. These health care workers are important personnel at the front line in the corresponding departments who perhaps experience more safety problems.[41] Hospital managers and policy makers should carefully consider their opinions.

Clinical auxiliary departments Need Attention

Another meaningful finding of our study was that the perceptions of patient safety climate among (associate) chief physicians and residents or below in the clinical auxiliary departments were worse than other staff within and outside the departments regarding many dimensions of safety climate and overall including organizational resources for safety, unit managers' support, unit recognition and support for safety efforts, collective learning, psychological safety, problem responsiveness，provision of safe care (7 of 12 dimensions), and overall safety climate. Hospital managers might not pay sufficient attention to the clinical auxiliary departments compared

For peer review only - http://bmjopen.bmj.com/site/about/guidelines.xhtml

with the clinical departments, especially the residents' training and motivation in this kind of departments.

Limitations

First, the data were based on self-reporting, which might involve recall/report bias. Second, because this study was a cross-sectional study, we could not rule out the potential for omitted variables. Although our analyses represented an important advance over prior studies because we adjusted for important known individual and hospital characteristics, other characteristics that were not measured could play some role in distinguishing personnel by working department and job type. Third, the results from 54 public general hospitals in three regions might not be generalizable to all hospitals in China, although our sample size was reasonably large and represented public hospitals in high, middle, and low socioeconomic level regions and the eastern, central, and western regions of China.

Conclusions

Our study highlighted differences in perceptions of patient safety climate among and within working departments and job types that were not previously documented in China. The findings indicated that safety climate improvement efforts should involve greater attention to the climate in the ICU, ED, pediatric, and clinical auxiliary departments. Therefore, it is a more effective and reasonable approach to implement specific measures to improve patient safety climate according to targeted departments and personnel.

Acknowledgements

We gratefully acknowledge the significant contributions of the following members of the research project team: Hongbo Zhu, Xuefeng Wei, Jianjun Gu. The authors also acknowledge all the hospitals that provided assistance with data collection in this research project.

For peer review only

Contributors

Conceived and designed the study: Di Xue. Performed the investigation: Di Xue, Fei Bai, Mingqi Li, Huiqing Tang, Jie Bai, Ping Zhou. Conducted statistical analyses and interpreted the data: Ping Zhou, Mingqi Li. Drafted the manuscript: Ping Zhou. Revised the manuscript: Di Xue. All authors read and approved the final manuscript.

Competing interests

The authors have declared that no competing interest exists.

Funding

This research project was supported by the National Natural Science Foundation of China, grant number 71473047.

Ethics approval

The study was approved by the Institutional Review Board of the School of Public Health, Fudan University (IRB# 2014-03-0502).

Data sharing statement

No additional data were available.

References

1. Cheng YM, Liu Y, Liu YM. [Review on research and application of adverse events reporting system in China]. *Chin Hosp Manage* 2012; 32: 40–2. (in Chinese).

For peer review only

2. Hu R. WHO Release of the Chinese Version of the Patient Safety Curriculum Guide: Multi-Professional Edition. *Chin Community Physician* 2012. p. 29. p. 23. (in Chinese).

3. Zhu J, Li L, Li Y, Shi M, Lu H, Garnick DW, *et al.* What constitutes patient safety culture in Chinese hospitals? *Int J Qual Health Care* 2012; 24: 250-7. doi: 10.1093/intqhc/mzs010.

4. [Internet] Xu SX, National Health and Family Planning Commission of PR. [Seven measures to improve medical quality and patient safety]. China Hospital CEO 25 Dec 2015. http://www.h-ceo.com/article/read/6978/6978.html (accessed 7 Aug 2016). (in Chinese).

5. Braithwaite J, Westbrook MT, Travaglia JF, Hughes C. Cultural and associated enablers of, and barriers to, adverse incident reporting. *Qual Saf Health Care* 2010; 19: 229–33. doi: 10.1136/qshc.2008.030213.

6. Singer S, Lin S, Falwell A, Gaba D, Baker L. Relationship of safety climate and safety performance in hospitals. *Health Serv Res* 2009; 44: 399–421. doi: 10.1111/j.1475-6773.2008.00918.x.

7. Mardon RE, Khanna K, Sorra J, Dyer N, Famolaro T. Exploring relationships between Hospital patient safety culture and adverse events. *J Patient Saf* 2010; 6: 226–32. doi: 10.1097/PTS.0b013e3181fd1a00.

8. Huang DT, Clermont G, Kong L, Weissfeld LA, Sexton JB, et al. Intensive care unit safety culture and outcomes: a US multicenter study. Int J Qual Health Care 2010,22: 151-161.

9. Hansen LO, Williams MV, Singer SJ. Perceptions of hospital safety climate and incidence of readmission. *Health Serv Res* 2011; 46: 596–616. doi: 10.1111/j.1475-6773.2010.01204.x.

10. Rose JS, Thomas CS, Tersigni A, Sexton JB, Pryor D. A leadership framework for culture change in health care. *Jt Comm J Qual Patient Saf* 2006; 32: 433– 42.

For peer review only

11. Flin R, Burns C, Mearns K, Yule S, Robertson EM. Measuring safety climate in health care. *Qual Saf Health Care* 2006; 15: 109–15. doi: 10.1136/qshc.2005.014761.

12. Singer SJ, Vogus TJ. Safety climate research: taking stock and looking forward. *BMJ Qual Saf* 2013; 22: 1–4. doi: 10.1136/bmjqs-2012-001572.

13. Makary MA, Sexton JB, Freischlag JA, Holzmueller CG, Millman EA, Rowen L, *et al.* Operating room teamwork among physicians and nurses: teamwork in the eye of the beholder. *J Am Coll Surg* 2006; 202: 746 –52. doi: 10.1016/j.jamcollsurg.2006.01.017.

14. Cooper JB, Blum RH, Carroll JS, Dershwitz M, Feinstein DM, Gaba DM, *et al.* Differences in safety climate among hospital anesthesia departments and the effect of a realistic simulation-based training program. *Anesth Analg* 2008; 106: 574 –84. doi: 10.1213/01.ane.0000296462.39953.d3.

15. Huang DT, Clermont G, Sexton JB, Karlo CA, Miller RG, Weissfeld LA, *et al.* Perceptions of safety culture vary across the intensive care units of a single institution. *Crit Care Med* 2007; 35: 165–76. doi: 10.1097/01.CCM.0000251505.76026.CF.

16. Singer SJ, Gaba DM, Falwell A, Lin S, Hayes J, Baker L. Patient safety climate in 92 US hospitals: differences by work area and discipline. *Med Care* 2009; 47: 23-31. doi: 10.1097/MLR.0b013e31817e189d.

17. Paine LA, Rosenstein BJ, Sexton JB, Kent P, Holzmueller CG, Pronovost PJ. Assessing and improving safety culture throughout an academic medical centre: a prospective cohort study. *Qual Saf Health Care* 2010; 19: 547-54. doi: 10.1136/qshc.2009.039347.

18. Gehring K, Mascherek AC, Bezzola P, Schwappach DL. Safety climate in Swiss hospital units: Swiss version of the Safety Climate Survey. *J Eval Clin Practice* 2015; 21: 332–8. doi: 10.1111/jep.12326.

19. Sullivan A, Camargo C, Cleary P, Gordon J, Kaushal R, Magid D, *et al.* Do emergency physicians and nurses differently perceive safety-related factors?

For peer review only

The National ED Safety Study [Abstract]. *Acad Emerg Med* 2007; 14 (Suppl): S210 –1. doi: 10.1197/j.aem.2007.03.1310.

20. Sexton JB, Holzmueller CG, Pronovost PJ, Thomas EJ, McFerran S, Nunes J, *et al.* Variation in caregiver perceptions of teamwork climate in labor and delivery units. *J Perinatol* 2006; 26: 463– 70. doi: 10.1038/sj.jp.7211556.
21. Thomas EJ, Sexton JB, Helmreich RL. Discrepant attitudes about teamwork among critical care nurses and physicians. *Crit Care Med* 2003; 31: 956–9. doi: 10.1097/01.CCM.0000056183.89175.76.
22. Zhou P, Bundorf MK, Gu J, He X, Xue D. Survey on patient safety climate in public hospitals in China. *BMC Health Serv Res* 2015; 15: 53. doi: 10.1186/s12913-015-0710-x.
23. Singer S, Meterko M, Baker L, Gaba D, Falwell A, Rosen A. Workforce perceptions of hospital safety culture: development and validation of the patient safety climate in healthcare organizations survey. *Health Serv Res* 2007; 42: 1999–2021. doi: 10.1111/j.1475-6773.2007.00706.x.
24. Hartmann CW, Rosen AK, Meterko M, Shokeen P, Zhao S, Singer S, *et al.* An overview of patient safety climate in the VA. *Health Serv Res* 2008; 43: 1263–84. doi: 10.1111/j.1475-6773.2008.00839.x.
25. Singer SJ, Hartmann CW, Hanchate A, Zhao S, Meterko M, Shokeen P, et al. Comparing safety climate between two populations of hospitals in the United States. *Health Serv Res* 2009; 44: 1563-84. doi: 10.1111/j.1475-6773.2009.00994.x.
26. Colla JB, Bracken AC, Kinney LM, et al. Measuring patient safety climate: a review of surveys. Qual Saf Health Care 2005;14:364-6.
27 Weick KE, Sutcliffe KM. Managing the Unexpected. San Francisco, CA: Jossey-Bass; 2001
28. Nie Y, Mao X, Cui H, He S, Li J, Zhang M. Hospital survey on patient safety culture in China. BMC Health Serv Res. 2013;13:228–38.

For peer review only

29. Chen IC, Li HH. Measuring patient safety culture in Taiwan using the Hospital Survey on Patient Safety Culture (HSOPSC). BMC Health Serv Res. 2010;10:152–61

30. Bentler PM, Bonett DG. Significance Tests and Goodness of Fit in the Analysis of Covariance Structures. *Psychol Bull*. 1980;88(3):588–606

31. McDonald RP, Ho MHR. Principles and practice in reporting structural Equation Analyses. *Psychological Methods,* 2002,7(1): 64-8232. Gefen D, Straub DW, Boudreau MC. Structural equation modeling and regression: guidelines for research practice. Communications of the association for information systems,2000,4(7): 1-77

33. Gaba DM, Howard SK, Jump B. Production pressure in the work environment. California anesthesiologists' attitudes and experiences. *Anaesthesiol* 1994; 81: 488–500. doi: 10.1097/00000542-199408000-00028.

34. Singer SJ, Gaba DM, Geppert JJ, Sinaiko AD, Howard SK, Park KC. The culture of safety: results of an organization-wide survey in 15 California hospitals. *Qual Saf Health Care* 2003; 12: 112–8. doi: 10.1097/MLR.0b013e31817925c1.

35. Singer SJ, Falwell A, Gaba DM, Baker LC. Patient safety climate in US hospitals: variation by management Level. *Med Care* 2008; 46: 1149–56.

36. Wang JC, Xie HY, Jiang BF. Multilevel models: methods and applications. Beijing: Higher Education Press, 2010

37. Gong TX. Impact of financial compensation policy: an intervention study in 15 hospitals of traditional Chinese medicine. Beijing: Beijing University of Chinese Medicine, 2012.

38. Zhou X, Wu EZ. The practice and discussion on performance appraisal reform in county-level public hospitals in Haining City. J Health Econ Res 2015;8:24-6, 27.

39. Gaba DM, Singer SJ, Sinaiko AD, Bowen JD, Ciavarelli AP. Differences in safety climate between Hospital personnel and Naval aviators. *Hum Factors* 2003; 45: 173–85. doi: 10.1518/hfes.45.2.175.27238.

For peer review only

40 Pronovost P, Weast B, Holzmueller C, et al. Evaluation of the culture of safety: survey of clinicians and managers in an academic medical center. *Qual Saf Health Care*. 2003;12:405– 410

41. He XY, Gu JJ, Sun X, et al. Medical quality control system in public general hospitals in Pudong New Area. Chinese Health Quality Management 2015;22(4):25-28. (in Chinese)

42. Wang YQ, Qin XY, Gao X, et al. Quality control circle in the continuous quality improvement campaign of the hospital. Chin J Hosp Admin 2014;30(6):425-427. (in Chinese)

43. Liu SJ, Xu MY. The practice of the assessment of medical quality in clinical departments in a general hospital. Chinese Hospital Management 2013,33(5):56-57 (in Chinese)

44. Wang LH, Zhao GG, Yang X, et al. On the practice and insights of building a safety-oriented culture in the hospital. Chin J Hosp Admin 2009;25(9):595-598. (in Chinese)

45. Wang LH, Zhao GG, Yang X, et al. On the practice and insights of building a safety-oriented culture in the hospital. *Chin J Hosp Admin,* 2009;25(9):595-598.(in Chinese)

46. Liu HS. Reinforcing internal communication to promote the harmonious development of the hospital. *China science and technology review*, 2009;(26):102.(in Chinese)

47. Ren YQ, Lu MH, Zhang Y, Ji QY. [Issues and solutions of building a pediatrician pool in Shanghai]. *Hospital Administration Journal of Chinese PLA* 2016; 23: 228-30. (in Chinese).

48. Liu SH, Pi GH, Kang Q, Zhao J, He Y. [Current situation of pediatric in Nanchong of Sichuan province]. *Journal of North Sichuan Medical College* 2015; 30: 109-111. (in Chinese).

49. [Internet] Xin Hua News. China faces shortage of pediatricians: http://news.xinhuanet.com/english/health/2014-09/11/c_133636236.htm (accessed 15 Sept 2016).

For peer review only

50. [Internet] Zhao CY. China faces critical pediatrician shortage. China Daily: http://www.chinadaily.com.cn/china/2011-02/17/content_12034787.htm (accessed 10 Sept 2016).

51. [Internet] China Network Television(CNTV). China facing pediatrician shortage: http://www.china.org.cn/video/2016-01/20/content_37620967.htm. (accessed 10 Sept 2016).

52. Pan SJ, Chen JM, Bai JG. Analysis and countermeasures on the causes of pediatric human resources shortage--Take Shanxi province as an example. *Soft science of Health* 2016,30(7): 31-34. (in Chinese).

53. Lin PH, Xiao HX, Lin XQ. [Survey on stress management of emergency physicians and its countermeasures in Fujian province]. *Chinese Rural Health Service Administration* 2014; 34: 503-6. (in Chinese).

54. Wang L, Liu ZF, Peng XW, Luan R. Present situation investigation and analysis of emergency departments of 20 tertiary general hospitals in Heilongjiang Province (in Chinese). *Management* 2015; 35: 68-9.

55. Sheng WJ, Zhang S, Zhang L. [Analysis on ICU healthcare providers' stress resources and its countermeasures]. *Chronic Pathematology Journal* 2010; 12: 1674-5. (in Chinese).

56. Li C, Tian XH, Feng LN, Zhang R. Study on correlation between job stress and coping styles and job burnout of ICU nurses (in Chinese). *Chinese Nurs Res* 2014; 28: 3236-7.

57. Teng CI, Shyu YI, Chiou WK, Fan HC, Lam SM. Interactive effects of nurse-experienced time pressure and burnout on patient safety: a cross-sectional survey. *Int J Nurs Stud* 2010; 47: 1442–50. doi: 10.1016/j.ijnurstu.2010.04.005.

58. West CP, Tan AD, Habermann TM, Sloan JA, Shanafelt TD. Association of resident fatigue and distress with perceived medical errors. *JAMA* 2009; 302: 1294–300. doi: 10.1001/jama.2009.1389.

59. Halbesleben JR, Rathert C. Linking physician burnout and patient outcomes: exploring the dyadic relationship between physicians and patients. *Health*

Care Manage Rev 2008; 33: 29–39. doi: 10.1097/01.HMR.0000304493.87898.72.

60. Nahrgang JD, Morgeson FP, Hofmann DA. Safety at work: a meta-analytic investigation of the link between job demands, job resources, burnout, engagement, and safety outcomes. *J Applpsychol* 2011; 96: 71–94. doi: 10.1037/a0021484.

61. Flinn F, Armstrong C. Junior doctors' extended work hours and the effects on their performance: the Irish case. *Int J Qual Healthcare* 2011; 23:210–217. doi: 10.1093/intqhc/mzq088

62. Sharpe R, Koval V, Ronco JJ, et al. The impact of prolonged continuous wakefulness on resident clinical performance in the intensive care unit: a patient simulator study. Crit Care Med 2010; 38:766–770. doi: 10.1097/CCM.0b013e3181cd122a

63. Valentin A. Approaches to decreasing medication and other care errors in the ICU. Curr Opin Crit Care, 2013,19(5): 474-479. doi: 10.1097/MCC.0b013e328364d4f9

64. Dehmel C, Braune SA, Kreymann G, et al. Do centrally preprepared solutions achieve more reliable drug concentrations than solutions prepared on the ward? Intensive Care Med 2011; 37:1311–6.

65. Poon EG, Keohane CA, Yoon CS, et al. Effect of bar-code technology on the safety of medication administration. N Engl J Med 2010; 362:1698–1707.

66. Askari M, Eslami S, Louws M, et al. Frequency and nature of drug-drug interactions in the intensive care unit. Pharmacoepidemiol Drug Saf 2013; 22:430–7.

67. Garrouste-Orgeas M, Soufir L, Tabah A, et al. A multifaceted program for improving quality of care in intensive care units: IATROREF study. Crit Care Med 2012; 40:468–76.

68. Wallace DJ, Angus DC, Barnato AE, et al. Nighttime intensivist staffing and mortality among critically ill patients. N Engl J Med 2012; 366:2093–101..

For peer review only

Tables and captions

Table 1 Problematic Responses for Dimensions and Items

Table 1 Problematic Responses for Dimensions and Items

**Dimensions and Items** †	**% Problematic**
Senior managers' engagement	2.84
Organizational resources for safety	9.55
Overall emphasis on patient safety	2.63
Unit managers' support	5.42
Unit safety norms	4.51
Unit recognition and support for safety efforts	6.68
Collective learning	2.10
Psychological safety	5.68
Problem responsiveness	2.61
Fear of shame	20.42
Fear of blame and punishment	64.81
Provision of safe care	16.31
Overall average	9.00

† Means of all items in a dimension were averaged to the calculated dimension mean.

For peer review only

Figures and legends

Figure 1. Heatmap of predictive means of staff among different working departments on each dimension and the overall safety climate.

Figure 2. Heatmap of predictive means of staff with different job types on each dimension and the overall safety climate.

Figure 3. Heatmap of predictive means of staff among 9 working departments across various title physicians and other staff on each dimension and the overall safety climate.

For peer review only

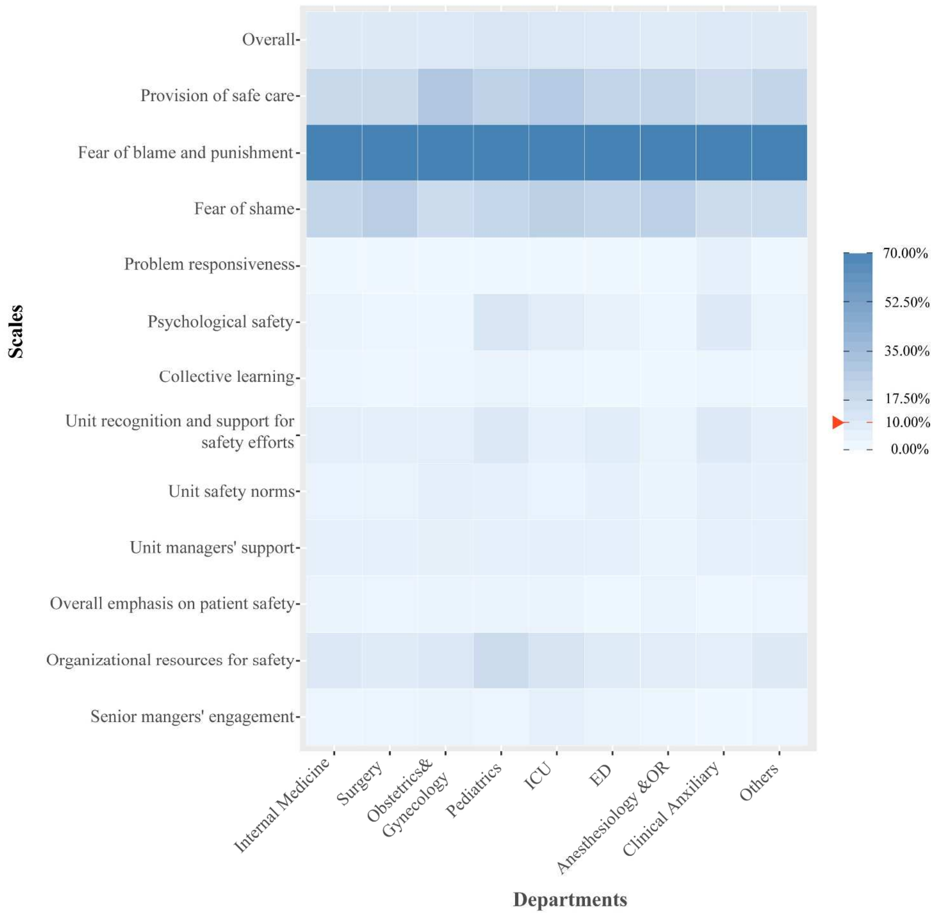

Figure 1. Heatmap of predictive means of staff among different working departments on each dimension and the overall safety climate

239x231mm (300 x 300 DPI)

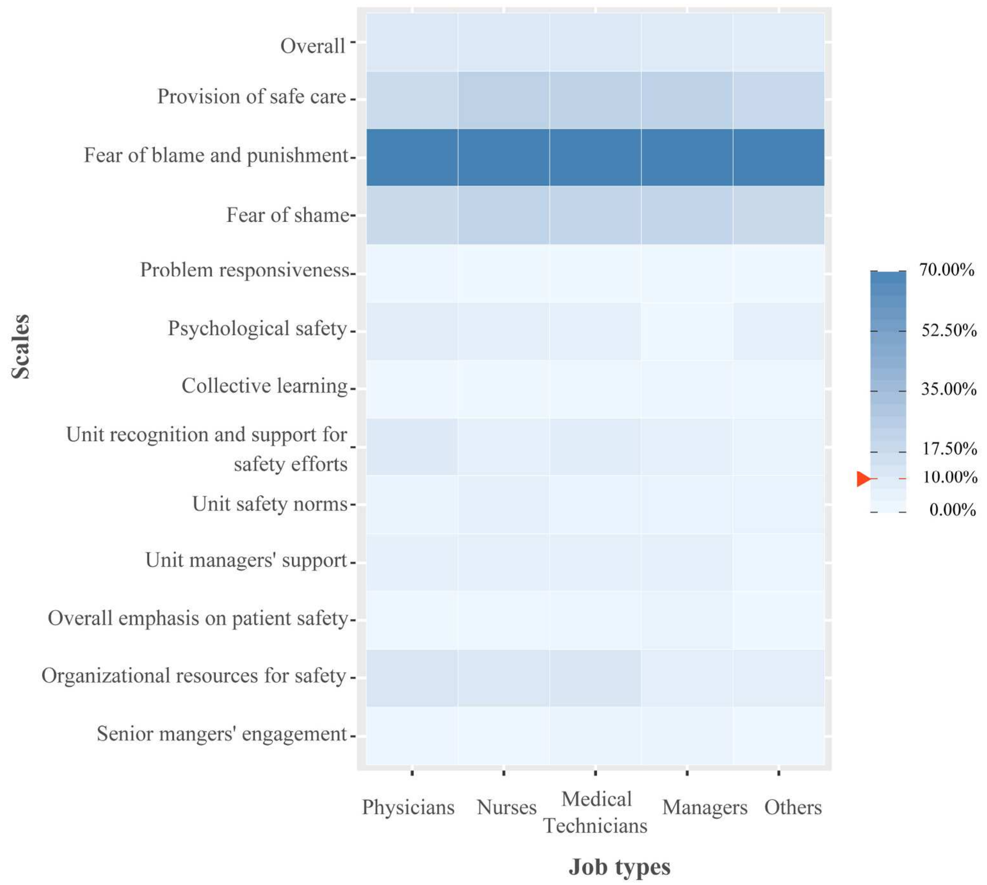

Figure 2. Heatmap of predictive means of staff with different job types on each dimension and the overall safety climate.

194x189mm (300 x 300 DPI)

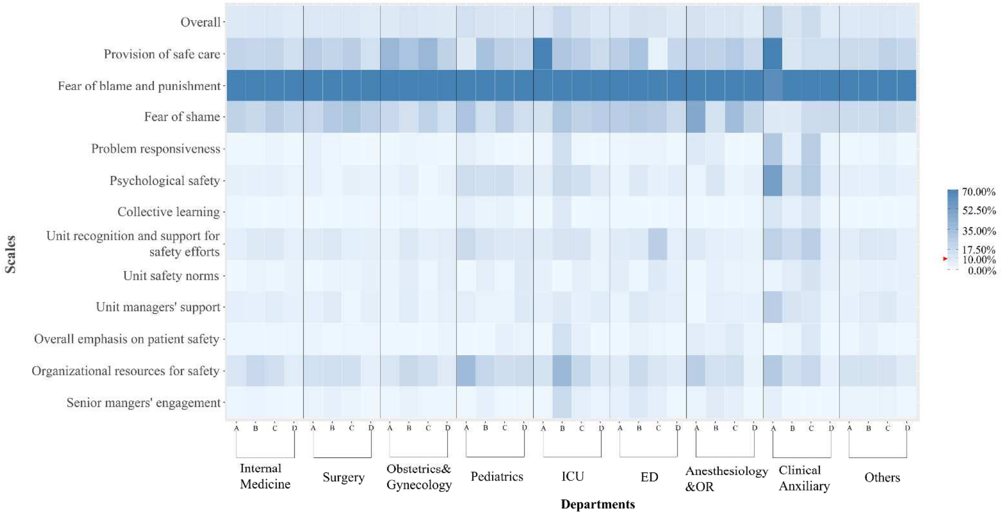

Figure 3. Heatmap of predictive means of staff among 9 working departments across various title physicians and other staff on each dimension and the overall safety climate.

Note:A- chief physicians or associate chief physicians; B-attending physicians; C-residents or below; D-others.

579x328mm (300 x 300 DPI)

For peer review only

Appendix A: General Respondent Information

Work Area Characteristics	N	%
Region		
Shanghai	2094	50.81
Hubei province	1095	26.57
Gansu province	932	22.62
Total	4121	100.00
Hospital level		
Tertiary	1827	44.33
Secondary	2294	55.67
Total	4121	100.00
Bed size(beds)		
Small (⩽500)	841	20.41
Middle (501-800)	1131	27.44
Large (>800)	2149	52.15
Total	4121	100.00
Affiliated with Medical School		
Yes	2644	65.08
No	1419	34.92
Total	4063	100.00
Working Department		
Internal Medicine	791	19.46
Surgery	772	18.99
Obstetrics-Gynecology	266	6.54
Pediatrics	128	3.15
ICU	146	3.59
Emergency Department (ED)	247	6.08
Anesthesiology	63	1.55
Operating Room (OR)	90	2.21
Clinical Auxiliary Departments†	549	13.51
Others	1013	24.92
Total	4065	100.00
Doctor-nurse ratio		
Mean (SD)	0.81	0.59
Bed-Nurse ratio		
Mean (SD)	2.53	2.52

Worker Characteristics	N	%
Gender		
Male	1365	33.69
Female	2687	66.31
Total	4052	100.00
Age		
<25	269	6.67
25-34	771	19.11
35-45	879	21.79
>=45	2115	52.43
Total	4034	100.00
Education		
Master's degree or above	737	18.03
Bachelor's degree	2205	53.94
Associate's degree or below	1146	28.03
Total	4088	100.00
Working years		
<5	1073	26.63
5-10	852	21.15
>10	2104	52.22
Total	4029	100.00
Monthly income (RMB)		
<4000	1311	32.25
4000-7999.99	1767	43.47
⩾8000	987	24.28
Total	4065	100.00
Job type		
Frontline physicians	1563	38.66
(Associate) Chief physicians	*417*	*10.32*
Attending physicians	*628*	*15.53*
Resident or below	*518*	*12.81*
Frontline nurses	1480	36.61
Medical technicians	375	9.28
Managers‡	116	2.87
Non-professionals and non-managers	509	12.58
Total	4043	100.00

† Referring to laboratory department, image department, ECG department, pathology department, pharmacy department, supply center, etc.

‡ Including the managers in the clinical departments, clinical auxiliary departments, and administrative departments.

For peer review only

Appendix B: **Patient Safety Climate by Working Department †**

Dimensions	Internal Medicine		Surgery		Obstetrics -Gynecology	
	Estimate	Confidence interval	Estimate	Confidence interval	Estimate	Confidence interval
Senior mangers' engagement	2.74	[1.58,3.90]	2.06	[0.87,3.25]	1.80	[0.21,3.39]
Organizational resources for safety	9.38	[6.90,11.86]	7.54	[5.00,10.09]	7.02	[3.65,10.40]
Overall emphasis on patient safety	2.96	[1.57,4.35]	2.19	[0.76,3.62]	1.56	[-0.38,3.49]
Unit managers' support	5.21	[3.40,7.01]	4.90	[3.05,6.76]	3.15	[0.65,5.64]
Unit safety norms	3.85	[2.67,5.03]	3.26	[2.05,4.47]	3.92	[2.29,5.55]
Unit recognition and support for safety efforts	5.95	[3.95,7.94]	5.51	[3.46,7.56]	4.29	[1.59,6.99]
Collective learning	2.14	[1.10,3.19]	1.40	[0.33,2.48]	0.91	[-0.53,2.35]
Psychological safety	2.87	[0.94,4.79]	1.83	[-0.15,3.80]	0.72	[-1.92,3.37]
Problem responsiveness	0.93	[-0.46,2.31]	0.42	[-1.00,1.84]	-0.40	[-2.30,1.49]
Fear of shame	19.77	[15.84,23.69]	23.51	[19.49,27.53]	12.46	[7.26,17.67]
Fear of blame and punishment	66.45	[61.69,71.21]	69.93	[65.06,74.79]	58.80	[52.60,65.01]
Provision of safe care	17.19	[13.45,20.93]	17.67	[13.84,21.50]	22.72	[17.75,27.70]
Overall	8.51	[7.57,9.46]	8.28	[7.30,9.25]	6.82	[5.52,8.11]

Continued:

Dimensions	Pediatrics		ICU		Emergency Department (ED)	
	Estimate	Confidence interval	Estimate	Confidence interval	Estimate	Confidence interval
Senior mangers' engagement	2.19	[0.02,4.35]	4.86	[2.84,6.87]	4.62	[2.97,6.28]
Organizational resources for safety	14.41	[9.85,18.97]	10.54	[6.28,14.79]	8.83	[5.32,12.35]
Overall emphasis on patient safety	2.60	[-0.04,5.24]	2.79	[0.33,5.26]	2.71	[0.70,4.73]
Unit managers' support	4.42	[1.02,7.82]	5.44	[2.27,8.62]	6.29	[3.69,8.89]
Unit safety norms	4.71	[2.50,6.92]	3.72	[1.66,5.79]	5.43	[3.73,7.12]
Unit recognition and support for safety efforts	8.57	[4.93,12.20]	5.10	[1.71,8.48]	8.33	[5.52,11.13]
Collective learning	3.17	[1.22,5.13]	2.54	[0.71,4.36]	2.60	[1.11,4.10]
Psychological safety	9.50	[5.90,13.09]	6.69	[3.34,10.04]	5.29	[2.54,8.05]
Problem responsiveness	0.75	[-1.81,3.32]	1.11	[-1.29,3.50]	2.02	[0.05,4.00]
Fear of shame	16.95	[10.01,23.88]	20.25	[13.78,26.71]	20.09	[14.68,25.50]
Fear of blame and punishment	61.47	[53.27,69.67]	60.06	[52.40,67.71]	65.68	[59.24,72.13]
Provision of safe care	19.48	[12.84,26.12]	22.22	[16.03,28.41]	19.94	[14.77,25.10]
Overall	9.46	[7.70,11.21]	9.19	[7.56,10.83]	9.63	[8.29,10.98]

Continued:

Dimensions	Anesthesiology + Operating Room (OR)		Clinical Auxiliary Departments†		Others	
	Estimate	Confidence interval	Estimate	Confidence interval	Estimate	Confidence interval
Senior mangers' engagement	1.87	[-0.11,3.84]	1.53	[0.27,2.80]	1.89	[0.85,2.94]
Organizational resources for safety	6.10	[1.93,10.28]	7.12	[4.43,9.81]	7.80	[5.55,10.05]
Overall emphasis on patient safety	3.26	[0.85,5.68]	2.44	[0.93,3.96]	1.87	[0.63,3.12]
Unit managers' support	2.85	0.26,5.96]	5.80	[3.84,7.77]	4.29	[2.67,5.91]
Unit safety norms	2.36	[0.34,4.38]	6.12	[4.83,7.40]	3.99	[2.93,5.06]
Unit recognition and support for safety efforts	3.03	[-0.30,6.36]	8.97	[6.80,11.13]	5.34	[3.52,7.15]
Collective learning	0.44	[-1.35,2.23]	3.20	[2.06,4.34]	0.91	[-0.03,1.85]
Psychological safety	2.28	[-1.01,5.56]	8.95	[6.86,11.05]	3.05	[1.32,4.78]
Problem responsiveness	0.43	[-1.92,2.78]	5.50	[4.00,7.01]	0.26	[-0.99,1.51]
Fear of shame	21.80	[15.44,28.15]	16.22	[11.99,20.45]	15.60	[11.99,19.20]
Fear of blame and punishment	66.92	[59.39,74.45]	68.73	[63.63,73.83]	67.12	[62.72,71.53]
Provision of safe care	19.52	[13.44,25.60]	16.54	[12.51,20.58]	18.94	[15.51,22.37]
Overall	7.44	[5.84,9.04]	9.52	[8.49,10.55]	7.79	[6.93,8.65]

† Results reflect predicted means (estimate and 95% confidence interval) by working departments based on 2-level random intercept models for each dimension and the overall safety climate adjusted for other individual characteristics (age, gender, education, working years and monthly income) and hospital characteristics (tertiary level vs secondary level, hospital size, hospital location, doctor-nurse ratio). When estimating the predicted mean values of the PPR for each dimension and overall by working department and job type, we held other covariates constant at their means.

Appendix C: **Patient Safety Climate by Job type †**

Dimensions	Physicians		Nurses		Medical technicians	
	Estimate	Confidence interval	Estimate	Confidence interval	Estimate	Confidence interval
Senior mangers' engagement	4.01	[3.20,4.81]	2.17	[1.28,3.07]	2.76	[0.95,4.58]
Organizational resources for safety	12.25	[10.48,14.02]	9.97	[8.02,11.92]	8.86	[5.04,12.69]
Overall emphasis on patient safety	3.36	[2.43,4.29]	2.37	[1.34,3.41]	1.83	[-0.38,4.04]
Unit managers' support	6.15	[4.92,7.38]	5.50	[4.13,6.86]	4.26	[1.41,7.10]
Unit safety norms	4.92	[4.11,5.74]	4.99	[4.09,5.90]	2.93	[1.07,4.78]
Unit recognition and support for safety efforts	9.78	[8.33,11.22]	5.45	[3.87,7.04]	6.19	[3.14,9.24]
Collective learning	2.86	[2.14,3.58]	1.80	[0.99,2.60]	0.75	[-0.89,2.39]
Psychological safety	8.30	[6.96,9.63]	5.84	[4.35,7.32]	3.90	[0.89,6.91]
Problem responsiveness	4.09	[3.12,5.07]	1.63	[0.56,2.71]	0.10	[-2.05,2.25]
Fear of shame	18.39	[15.43,21.35]	19.76	[16.56,22.97]	17.48	[11.64,23.33]
Fear of blame and punishment	65.04	[61.35,68.73]	63.07	[59.10,67.03]	62.67	[55.72,69.61]
Provision of safe care	17.57	[14.76,20.37]	21.07	[18.03,24.11]	18.98	[13.39,24.58]
Overall	10.19	[9.52,10.86]	8.83	[8.09,9.57]	7.89	[6.42,9.36]

Continued:

Dimensions	Managers ‡		Non-professionals and non-managers	
	Estimate	Confidence interval	Estimate	Confidence interval
Senior mangers' engagement	2.39	[-0.06,4.83]	1.76	[0.49,3.03]
Organizational resources for safety	5.71	[0.56,10.85]	6.96	[4.25,9.67]
Overall emphasis on patient safety	3.12	[0.11,6.13]	1.76	[0.24,3.28]
Unit managers' support	4.38	[0.52,8.24]	3.25	[1.28,5.22]
Unit safety norms	3.36	[0.85,5.86]	4.56	[3.27,5.85]
Unit recognition and support for safety efforts	4.70	[0.60,8.79]	4.48	[2.30,6.65]
Collective learning	1.72	[-0.49,3.94]	2.49	[1.35,3.64]
Psychological safety	-0.41	[-4.48,3.65]	5.26	[3.15,7.36]
Problem responsiveness	-1.32	[-4.22,1.58]	1.61	[0.10,3.12]
Fear of shame	18.95	[11.19,26.72]	17.99	[13.73,22.24]
Fear of blame and punishment	66.40	[57.25,75.54]	67.92	[62.78,73.06]
Provision of safe care	20.67	[13.24,28.11]	18.50	[14.45,22.56]
Overall	7.45	[5.47,9.43]	8.21	[7.18,9.25]

† Results reflect predicted means (estimate and 95% confidence interval) by job types based on 2-level random intercept models for each dimension and the overall safety climate adjusted for other individual † Results reflect predicted means (estimate and 95% confidence interval) by working departments based on 2-level random intercept models for each dimension and the overall safety climate adjusted for other individual characteristics (age, gender, education, working years, and monthly income) and hospital characteristics (tertiary level vs secondary level, hospital size, hospital location, and doctor-nurse ratio). When estimating the predicted mean values of the PPR for each dimension and overall by working department and job type, we held other covariates constant at their means.

‡ Including the managers in the clinical departments, clinical auxiliary departments, and administrative departments.

Appendix D: Differences of Patient Safety Climate among Job Types (pair-wise comparison with Managers) †

Dimensions	Physicians-Managers		Nurses-Managers		Medical technicians -Managers		Others-Managers	
	Estimate	Confidence interval	Estimate	Confidence interval	Estimate	Confidence interval	Estimate	Confidence interval
Senior mangers' engagement	1.62	[-1.38,4.62]	-0.21	[-3.36,2.94]	0.38	[-2.47,3.23]	-0.63	[-3.74,2.49]
Organizational resources for safety	6.55	[0.28,12.82]	4.27	[-2.33,10.86]	3.16	[-2.81,9.12]	1.25	[-5.27,7.78]
Overall emphasis on patient safety	0.24	[-3.47,3.94]	-0.75	[-4.64,3.15]	-1.29	[-4.82,2.23]	-1.36	[-5.21,2.00]
Unit managers' support	1.76	[-2.98,6.51]	1.11	[-3.87,6.10]	-0.13	[-4.64,4.38]	-1.13	[-6.07,3.80]
Unit safety norms	1.57	[-1.50,4.64]	1.64	[-1.60,4.87]	-0.43	[-3.35,2.49]	1.21	[-1.99,4.40]
Unit recognition and support for safety efforts	5.08	[0.11,10.05]	0.76	[-4.47,5.99]	1.50	[-3.23,6.23]	-0.22	[-5.39,4.96]
Collective learning	1.13	[-1.58,3.85]	0.07	[-2.78,2.93]	-0.97	[-3.56,1.61]	0.77	[-2.05,3.60]
Psychological safety	8.71	[3.73,13.70]	6.25	[1.01,11.49]	4.31	[-0.43,9.06]	5.67	[0.48,10.85]
Problem responsiveness	5.41	[1.86,8.96]	2.95	[-0.78,6.68]	1.42	[-1.96,4.79]	2.93	[-0.76,6.62]
Fear of shame	-0.57	[-9.89,8.76]	0.81	[-9.00,10.62]	-1.47	[-10.34,7.41]	-0.97	[-10.68,8.74]
Fear of blame and punishment	-1.35	[-12.23,9.52]	-3.33	[-14.78,8.11]	-3.73	[-14.08,6.63]	1.52	[-9.81,12.85]
Provision of safe care	-3.11	[-12.05,5.84]	0.39	[-9.02,9.81]	-1.69	[-10.21,6.83]	-2.17	[-11.49,7.15]
Overall	2.74	[0.32,5.15]	1.38	[-1.16,3.92]	0.44	[-1.85,2.74]	0.76	[-1.75,3.28]

† Results reflect differences of predicted values (estimate and 95% confidence interval) among job types by pair-wise comparison with Managers, based on 2-level random intercept models for each dimension and the overall safety climate adjusted for other individual characteristics (age, gender, education, working years, and monthly income) and hospital characteristics (tertiary level vs secondary level, hospital size, hospital location, and doctor-nurse ratio). When estimating the predicted mean values of the PPR for each dimension and overall by working department and job type, we held other covariates constant at their means.

Appendix E: Patient Safety Climate by Job Type across Working Departments[†] mean/standard error

Dimensions	Internal Medicine				Surgery				Obstetrics &Gynecology			
	A	B	C	D	A	B	C	D	A	B	C	D
Senior mangers' engagement	3.98/1.21	6.83/1.00	3.06/1.07	1.73/0.77	3.77/1.24	5.61/1.03	3.88/1.23	0.57/0.78	2.43/2.13	4.18/1.64	1.99/1.75	1.47/1.05
Organizational resources for safety	12.12/2.56	18.95/2.11	14.48/2.25	7.52/1.63	12.88/2.62	14.95/2.18	12.86/2.58	5.80/1.67	7.41/4.48	14.04/3.44	11.45/3.67	6.70/2.23
Overall emphasis on patient safety	3.63/1.49	5.33/1.22	2.98/1.31	2.75/0.93	3.33/1.53	2.69/1.26	2.69/1.51	2.42/0.95	2.36/2.63	1.71/2.02	1.12/2.15	2.34/1.29
Unit managers' support	6.91/1.92	7.44/1.57	7.72/1.68	5.06/1.20	5.43/1.97	8.49/1.62	2.65/1.94	5.95/1.23	3.08/3.38	6.53/2.59	2.79/2.77	4.14/1.66
Unit safety norms	2.34/1.24	6.76/1.02	3.52/1.09	4.43/0.78	2.18/1.27	3.95/1.05	3.18/1.26	4.48/0.80	3.01/2.18	6.10/1.68	2.23/1.79	5.18/1.08
Unit recognition and support for safety efforts	7.10/2.03	11.04/1.68	10.14/1.79	5.39/1.30	7.71/2.08	10.54/1.73	6.30/2.05	5.74/1.33	3.74/3.55	8.11/2.73	5.00/2.91	5.68/1.77
Collective learning	3.47/1.09	4.26/0.90	2.02/0.96	2.02/0.69	1.53/1.12	1.89/0.93	2.37/1.11	1.96/0.70	1.79/1.93	2.95/1.48	1.00/1.58	1.07/0.95
Psychological safety	6.21/2.01	7.51/1.66	6.33/1.77	3.80/1.27	5.54/2.07	3.45/1.71	4.88/2.04	4.15/1.30	2.98/3.55	5.18/2.72	1.13/2.90	3.09/1.75
Problem responsiveness	2.62/1.43	4.92/1.18	4.05/1.26	1.33/0.91	4.76/1.47	3.35/1.22	1.37/1.45	0.99/0.93	2.01/2.52	3.22/1.93	2.18/2.06	0.09/1.25
Fear of shame	21.99/3.87	18.74/3.21	23.22/3.42	18.60/2.52	17.25/3.96	26.14/3.31	25.96/3.90	24.1/2.57	11.61/6.70	10.68/5.17	17.82/5.51	12.05/3.39
Fear of blame and punishment	67.05/4.54	62.64/3.78	63.77/4.02	65.09/3.00	61.75/4.65	66.77/3.90	59.89/4.58	74.02/3.06	40.83/7.81	50.88/6.04	54.80/6.43	64.76/3.99
Provision of safe care	21.23/3.69	19.64/3.06	19.63/3.26	14.16/2.40	22.02/3.78	20.29/3.16	21.14/3.72	14.21/2.45	22.81/6.4	22.71/4.94	29.97/5.26	19.91/3.23
Overall	9.83/0.98	11.90/0.81	10.30/0.86	8.05/0.62	9.17/1.01	10.88/0.83	9.29/0.99	8.54/0.64	6.30/1.72	9.05/1.32	7.77/1.41	7.54/0.85

For peer review only

Dimensions	Pediatrics				ICU				ED			
	A	B	C	D	A	B	C	D	A	B	C	D
Senior mangers' engagement	4.43/2.95	3.40/2.26	4.18/2.19	1.52/1.59	0.71/2.95	16.00/2.93	6.97/2.15	3.42/1.29	4.14/2.13	9.29/1.75	9.24/2.02	2.78/1.07
Organizational resources for safety	37.03/6.19	17.64/4.76	18.12/4.60	10.62/3.35	10.77/6.19	25.18/6.14	17.71/4.53	8.39/2.71	7.06/4.48	15.10/3.67	13.22/4.25	9.32/2.26
Overall emphasis on patient safety	3.56/3.65	-0.33/2.80	7.03/2.70	2.68/1.95	0.64/3.65	14.48/3.62	5.50/2.66	1.07/1.58	0.33/2.63	5.15/2.15	6.50/2.50	2.22/1.31
Unit managers' support	9.11/4.68	2.25/3.59	5.70/3.47	5.98/2.51	4.92/4.68	12.24/4.65	5.36/3.41	6.21/2.04	2.64/3.38	8.67/2.77	9.95/3.21	7.26/1.69
Unit safety norms	4.22/3.02	4.91/2.32	5.02/2.24	6.09/1.62	3.70/3.02	8.61/3.00	6.37/2.21	3.32/1.32	5.92/2.18	3.36/1.79	11.25/2.07	5.78/1.09
Unit recognition and support for safety efforts	20.28/4.90	10.52/3.77	12.23/3.64	6.74/2.66	7.05/4.90	13.78/4.86	11.54/3.59	3.56/2.15	7.26/3.55	8.82/2.91	23.32/3.37	7.40/1.80
Collective learning	8.23/2.67	2.63/2.05	6.64/1.98	1.50/1.43	-0.12/2.67	12.46/2.65	2.17/1.95	2.38/1.16	-0.10/1.93	2.49/1.58	5.21/1.83	3.50/0.96
Psychological safety	18.01/4.91	13.34/3.77	18.12/3.64	6.76/2.64	5.59/4.90	16.33/4.87	13.87/3.58	6.87/2.14	2.15/3.55	8.59/2.90	8.29/3.36	8.26/1.78
Problem responsiveness	8.90/3.48	1.67/2.67	3.26/2.59	0.98/1.88	0.93/3.48	15.07/3.46	2.97/2.54	0.71/1.52	1.02/2.52	4.04/2.06	7.07/2.39	3.11/1.27
Fear of shame	31.58/9.24	13.57/7.11	26.76/6.88	10.19/5.05	11.77/9.23	21.65/9.16	20.20/6.77	21.7/4.09	23.63/6.70	21.95/5.51	24.09/6.36	17.96/3.44
Fear of blame and punishment	71.40/10.76	67.35/8.29	73.89/8.02	45.77/5.92	66.06/10.74	40.27/10.66	60.27/7.89	60.99/4.79	67.97/7.81	54.52/6.43	60.71/7.42	67.83/4.05
Provision of safe care	11.31/8.83	29.56/6.80	26.17/6.57	13.69/4.83	65.10/8.82	21.21/8.76	21.59/6.47	14.57/3.90	22.86/6.40	25.02/5.26	8.06/6.08	19.77/3.28
Overall	16.07/2.38	10.75/1.83	13.60/1.77	7.33/1.28	9.85/2.38	16.61/2.36	11.96/1.74	8.34/1.04	8.86/1.72	11.27/1.41	13.71/1.63	9.78/0.87

For peer review only

Dimensions	Anesthesiology &OR				Clinical Auxiliary Departments				Others			
	A	B	C	D	A	B	C	D	A	B	C	D
Senior mangers' engagement	1.75/2.86	6.85/2.31	5.08/2.48	0.24/1.26	3.17/3.19	1.02/2.47	4.75/2.36	0.92/0.99	3.06/1.43	3.68/1.27	3.02/1.34	1.32/0.56
Organizational resources for safety	16.31/6.00	11.56/4.86	15.54/5.21	3.48/2.65	13.66/6.69	12.91/5.19	18.58/4.96	4.91/2.08	11.39/3.00	12.27/2.67	11.16/2.82	7.61/1.20
Overall emphasis on patient safety	3.88/3.53	4.99/2.86	7.47/3.06	2.28/1.54	0.57/3.95	3.25/3.06	11.64/2.92	0.78/1.20	1.77/1.76	4.56/1.55	1.23/1.65	1.75/0.66
Unit managers' support	-0.10/4.53	5.46/3.67	4.20/3.93	3.96/1.99	12.18/5.06	11.10/3.92	11.63/3.75	3.44/1.55	4.08/2.26	7.24/2.00	7.71/2.12	4.29/0.87
Unit safety norms	0.70/2.93	4.85/2.37	2.40/2.54	3.01/1.29	1.58/3.27	6.83/2.53	13.79/2.42	5.63/1.01	3.77/1.46	5.18/1.30	3.89/1.37	4.72/0.57
Unit recognition and support for safety efforts	5.80/4.75	5.31/3.85	4.41/4.13	3.66/2.10	11.52/5.30	14.74/4.11	22.95/3.93	6.03/1.66	6.65/2.38	9.72/2.12	8.30/2.24	5.11/0.97
Collective learning	-0.37/2.58	1.28/2.09	-0.42/2.24	1.45/1.13	5.77/2.89	4.89/2.24	12.49/2.14	1.33/0.89	0.66/1.29	1.18/1.14	0.17/1.21	1.55/0.50
Psychological safety	1.52/4.75	8.93/3.85	1.65/4.13	4.68/2.09	26.71/5.31	14.04/4.11	24.91/3.93	5.85/1.63	5.63/2.37	5.16/2.10	6.13/2.22	4.54/0.92
Problem responsiveness	5.14/3.37	6.30/2.73	-0.69/2.93	0.82/1.49	14.16/3.77	5.57/2.92	23.23/2.79	2.89/1.17	1.33/1.69	2.69/1.50	3.43/1.58	0.76/0.66
Fear of shame	32.43/8.95	10.61/7.26	37.35/7.78	19.34/4.00	4.61/9.97	8.06/7.74	17.04/7.40	17.89/3.17	15.11/4.52	15.78/4.03	17.91/4.25	15.64/1.92
Fear of blame and punishment	49.52/10.42	55.99/8.46	78.23/9.07	68.60/4.69	31.77/11.60	62.70/9.02	59.12/8.62	74.83/3.74	63.58/5.29	65.28/4.73	65.98/4.98	67.66/2.32
Provision of safe care	15.77/8.55	17.89/6.94	28.28/7.44	17.88/3.82	35.64/9.53	9.90/7.40	15.79/7.07	15.97/3.03	16.05/4.31	16.32/3.85	20.12/4.06	18.77/1.82
Overall	8.06/2.30	9.22/1.87	10.92/2.00	7.33/1.02	11.13/2.57	10.50/1.99	17.25/1.9	8.38/0.80	8.28/1.15	9.51/1.02	9.32/1.08	8.02/0.46

† Results reflect predicted values (mean and standard errors) among various job types (referring to (associate) chief physicians, attending physicians, residents or below, and others) within one particular type of departments on each dimension and the overall safety climate, based on 2-level random intercept models with interactions of various titles of physicians and 9 working departments adjusted for other individual characteristics (age, gender, education, working years, and monthly income) and hospital characteristics (tertiary level vs secondary level, hospital size, hospital location, and doctor-nurse ratio). When estimating the predicted mean values of the PPR for each dimension and overall by working department and job type, we held other covariates constant at their means.

‡ A- chief physicians or associate chief physicians; B-attending physicians; C-residents or below; D-others.

For peer review only

STROBE Statement—checklist of items that should be included in reports of observational studies

	Item No	Recommendation	Our research
**Title and abstract**	1	(*a*) Indicate the study's design with a commonly used term in the title or the abstract	P1
		(*b*) Provide in the abstract an informative and balanced summary of what was done and what was found	P2
**Introduction**			
Background/rationale	2	Explain the scientific background and rationale for the investigation being reported	P4-5
Objectives	3	State specific objectives, including any prespecified hypotheses	P
**Methods**			
Study design	4	Present key elements of study design early in the paper	P5-10
Setting	5	Describe the setting, locations, and relevant dates, including periods of recruitment, exposure, follow-up, and data collection	P6-7
Participants	6	(*a*) *Cohort study*—Give the eligibility criteria, and the sources and methods of selection of participants. Describe methods of follow-up	-
		Case-control study—Give the eligibility criteria, and the sources and methods of case ascertainment and control selection. Give the rationale for the choice of cases and controls	_
		Cross-sectional study—Give the eligibility criteria, and the sources and methods of selection of participants	P7
		(*b*) *Cohort study*—For matched studies, give matching criteria and number of exposed and unexposed	_
		Case-control study—For matched studies, give matching criteria and the number of controls per case	_
Variables	7	Clearly define all outcomes, exposures, predictors, potential confounders, and effect modifiers. Give diagnostic criteria, if applicable	P9-10
Data sources/ measurement	8*	For each variable of interest, give sources of data and details of methods of assessment (measurement). Describe comparability of assessment methods if there is more than one group	P5-6
Bias	9	Describe any efforts to address potential sources of bias	P9
Study size	10	Explain how the study size was arrived at	P6-7
Quantitative variables	11	Explain how quantitative variables were handled in the analyses. If applicable, describe which groupings were chosen and why	P8-10
Statistical methods	12	(*a*) Describe all statistical methods, including those used to control for confounding	P8-10
		(*b*) Describe any methods used to examine subgroups and interactions	P8-10
		(*c*) Explain how missing data were addressed	-
		(*d*) *Cohort study*—If applicable, explain how loss to follow-up was addressed	_
		Case-control study—If applicable, explain how matching of cases and controls was addressed	_
		Cross-sectional study—If applicable, describe analytical methods	P8-10

For peer review only - http://bmjopen.bmj.com/site/about/guidelines.xhtml

taking account of sampling strategy		
(*e*) Describe any sensitivity analyses	_	

Continued on next page

For peer review only

Results			Our research
Participants	13*	(a) Report numbers of individuals at each stage of study—eg numbers potentially eligible, examined for eligibility, confirmed eligible, included in the study, completing follow-up, and analysed	-
		(b) Give reasons for non-participation at each stage	-
		(c) Consider use of a flow diagram	-
Descriptive data	14*	(a) Give characteristics of study participants (eg demographic, clinical, social) and information on exposures and potential confounders	P10-11
		(b) Indicate number of participants with missing data for each variable of interest	-
		(c) *Cohort study*—Summarise follow-up time (eg, average and total amount)	-
Outcome data	15*	*Cohort study*—Report numbers of outcome events or summary measures over time	-
		Case-control study—Report numbers in each exposure category, or summary measures of exposure	-
		Cross-sectional study—Report numbers of outcome events or summary measures	P10
Main results	16	(*a*) Give unadjusted estimates and, if applicable, confounder-adjusted estimates and their precision (eg, 95% confidence interval). Make clear which confounders were adjusted for and why they were included	P10-P12
		(*b*) Report category boundaries when continuous variables were categorized	-
		(*c*) If relevant, consider translating estimates of relative risk into absolute risk for a meaningful time period	-
Other analyses	17	Report other analyses done—eg analyses of subgroups and interactions, and sensitivity analyses	P12
**Discussion**			
Key results	18	Summarise key results with reference to study objectives	P12-17
Limitations	19	Discuss limitations of the study, taking into account sources of potential bias or imprecision. Discuss both direction and magnitude of any potential bias	P17
Interpretation	20	Give a cautious overall interpretation of results considering objectives, limitations, multiplicity of analyses, results from similar studies, and other relevant evidence	P12-17
Generalisability	21	Discuss the generalisability (external validity) of the study results	P17
**Other information**			
Funding	22	Give the source of funding and the role of the funders for the present study and, if applicable, for the original study on which the present article is based	P18

*Give information separately for cases and controls in case-control studies and, if applicable, for exposed and unexposed groups in cohort and cross-sectional studies.

Note: An Explanation and Elaboration article discusses each checklist item and gives methodological background and published examples of transparent reporting. The STROBE checklist is best used in conjunction with this article (freely available on the Web sites of PLoS Medicine at http://www.plosmedicine.org/, Annals of Internal Medicine at http://www.annals.org/, and Epidemiology at http://www.epidem.com/). Information on the STROBE Initiative is available at www.strobe-statement.org.

For peer review only

BMJ Open

Patient Safety Climate in General Public Hospitals in China: Differences Associated with Department and Job Type Based on a Cross-sectional Survey

Journal:	*BMJ Open*
Manuscript ID	bmjopen-2016-015604.R2
Article Type:	Research
Date Submitted by the Author:	16-Sep-2017
Complete List of Authors:	Zhou, Ping; Fudan University, School of Public Health<br>Bai, Fei; National center for medical service administration<br>Tang, Huiqing; Health and Family Planning Commission of Hubei Province<br>Bai, Jie; Fudan University School of Public Health<br>Li, Minqi; Fudan University School of Public Health<br>Xue, Di; Fudan University School of Public Health
**Primary Subject Heading**:	Health services research
Secondary Subject Heading:	Medical management, Patient-centred medicine
Keywords:	patient safety climate, working department, job type, China, Public hospital

SCHOLARONE™
Manuscripts

Patient Safety Climate in General Public Hospitals in China: Differences Associated with Department and Job Type Based on a Cross-sectional Survey

Authors

Ping Zhou[1], Fei Bai[2], Huiqing Tang[3], Jie Bai[1], Minqi Li[1], Di Xue[1]

[1] Key Laboratory of Health Technology Assessment (MOH); School of Public Health, Fu Dan University, Shanghai 200032, P.R. China

[2] National Center for Medical Service Administration, Beijing, 100044, P.R. China

[3] Health and Family Planning Commission of Hubei Province, Wuhan, 430071, P.R. China

Corresponding author:

Di Xue

Address: P.O. Box 197, 138 Yi Xue-yuan Rd., Shanghai, 200032, P.R. China

Email: xuedi@shmu.edu.cn

Fax: 86-21-33565181

Word count for the main text of the manuscript: 3824

For peer review only

Abstract (238 words)

Objective: This study analyzed differences in the perceived patient safety climate among different working departments and job types in general public hospitals in China.

Design: Cross-sectional survey.

Setting: Eighteen tertiary hospitals and 36 secondary hospitals from 10 areas in Shanghai, Hubei Province and Gansu Province, China.

Participants: Overall, 4,753 staff, including physicians, nurses, medical technicians, and managers, were recruited from March to June 2015.

Main outcome measure: The Patient Safety Climate in Health Care Organizations (PSCHO) tool and the percentages of "problematic responses" (PPRs) were used as outcome measures. Multivariable two-level random intercept models were applied in the analysis.

Results: A total of 4,121 valid questionnaires were collected. Perceptions regarding the patient safety climate varied among departments and job types. Physicians responded with relatively more negative evaluations of "organizational resources for safety," "unit recognition and support for safety efforts," "psychological safety," "problem responsiveness" and overall safety climate. Pediatrics departments, intensive care units (ICUs), emergency departments (EDs) and clinical auxiliary departments (CADs) require more attention. PPRs of "fear of blame and punishment" were universally significantly high, and PPRs of "fear of shame" and "provision of safe care" were remarkably high, especially in some departments. Department differences across all dimensions and the overall safety climate primarily depended on job type.

Conclusions: The differences suggest that strategies and measures for improving the patient safety climate should be tailored to working department and job type.

Keywords

patient safety climate, working departments, job type, public hospital, China

For peer review only

Strengths and limitations of this study

- This study was conducted in Shanghai Municipality, Hubei Province and Gansu Province, which represent high, middle, and low socioeconomic status levels in the eastern, central and western regions of China, using a large sample of 4,176 respondents from public general hospitals.
- This study is the first to investigate variations in the perceived patient safety climate among different departments and job types and their interaction in China's public general hospitals.
- We used 2-level random intercept models to examine the relationships between working departments/job types and the patient safety climate survey responses.
- To depict differences by job type within selected working departments, we incorporated the interaction of the working department and job type variables in the model and graphically displayed the predictions using a heat map.
- Although our analyses represent an important advance over prior studies because we adjusted for important known individual and hospital characteristics, other characteristics that were not measured could play a role in distinguishing personnel by working department and job type.
- The results from 54 public general hospitals in three regions might not be generalizable to all hospitals in China, although our sample size was large and represented public hospitals in high, middle, and low socioeconomic level regions in the eastern, central, and western regions of China.

For peer review only

Introduction

Patient safety is a core issue in health care services. Both hospitals and the Chinese government have made great efforts to strengthen the patient safety climate and improve patient safety performance.[1-4]

Because the patient safety climate is associated with positive outcomes such as greater error reporting,[5] fewer adverse events,[6,7] lower mortality rates,[8] and lower readmission rates,[9] measuring patient safety climate and understanding its variations can be helpful in targeting efforts to improve patient safety.[10-12] However, the patient safety climate can vary within organizations. Previous studies have indicated that the patient safety climate of particular departments varies both across and within institutions.[13-17] Therefore, neglecting the patient safety climate at the unit level will mask important local variations, and measuring departments' patient safety climate can identify important opportunities for improvement.

The previous literature has suggested that variations in the patient safety climate may be related to the pace and complexity of the work performed in different work areas.[15,16] Most studies investigating unit-specific climates have focused on measuring the climate in one or more types of departments with higher levels of intrinsic risk, such as the operating room (OR), the intensive care unit (ICU), and/or the emergency department (ED).[13-16,18] However, research concerning the patient safety climates of other units, such as the pediatric, internal medicine, surgery, and clinical auxiliary departments, is insufficient.

Some studies have measured the perception of patient safety climate among personnel by job type.[14,19-22] In some studies, physicians demonstrated more positive perceptions of patient safety climate than did nurses and other clinical personnel.[16,18,21] However, in a previous study conducted in hospitals in Pudong

For peer review only

New Area, Shanghai (a Chinese municipality), we found that physicians had more negative perceptions of the patient safety climate than did nurses.[22]

Given the variations in the perception of patient safety climate among different departments and job types, efforts to improve the patient safety climate should not be limited to interventions at the hospital level but should extend to the department level and different types of employees. Few articles have focused on patient safety climates at the department level and among different types of employees in hospitals in China.

This study analyzed differences in the perception of the patient safety climate among different working departments and job types in 54 public hospitals located in the eastern, central and western regions of China. We selected not only intrinsically hazardous departments but also other departments, including the internal medicine, surgery, obstetrics and gynecology, pediatric, and clinical auxiliary departments. We specifically explored: (1) in which departments the staff perceived the patient safety climate more negatively; (2) in which job types the staff rated lower scores across safety climate dimensions; and (3) whether there were differences by job type in the selected working departments.

Methods

Survey instrument

In the study, we applied the Patient Safety Climate in Healthcare Organizations (PSCHO) tool[23-25] to measure patient safety climate. Although various instruments are available to measure a hospital's safety climate,[26] we selected the PSCHO tool because of its good reliability and validity[22,24,25]. Moreover, it includes "fear of blame" and "fear of shame" to measure potential barriers to improving patient safety,[27] both of which capture underlying characteristics of Chinese culture.[22,28,29]

For peer review only

The PSCHO contains 12 dimensions and four categories (based on hospital, work unit, interpersonal contributions to the safety climate and other aspects of the safety climate).[23,24] The PSCHO items use a five-point Likert scale ranging from "strongly disagree" to "strongly agree," with a neutral midpoint.

In this study, we added two items to the PSCHO: "Staff can freely voice their opinions on patient safety" in the dimension "Psychological Safety" and "We analyze accidents or unexpected events in a timely manner" in the dimension "Problem Responsiveness." These items were added because they reflect a more general psychological safety climate (not specific to certain concerns) and timely responses to adverse events in hospitals, respectively. The survey also asked informants to provide demographic information, including gender, age, education, working years, monthly income, working department and job type.

Sample

A cross-sectional survey was conducted using a stratified sampling method in Shanghai Municipality, Hubei Province and Gansu Province in China from March to June of 2015. The regions were selected to capture various socioeconomic statuses and geographic distributions within China. First, we selected 3 provinces/municipalities representing high, middle, and low socioeconomic status levels located in the eastern, central, and western regions of China.

Hubei Province has 12 prefecture-level cities and 1 autonomous prefecture. Gansu Province has 12 prefecture-level cities and 2 autonomous prefectures. We selected 3 prefecture-level cities or autonomous prefectures (areas) in Hubei Province and Gansu Province, respectively, to represent high, middle, and low socioeconomic status levels within each province. In each area, two tertiary public general hospitals and four secondary public general hospitals were selected.

For peer review only

Shanghai Municipality has 16 districts. Because Shanghai's tertiary hospitals are not evenly distributed among these districts, 6 tertiary public general hospitals were selected to represent tertiary hospitals owned by universities, the Shanghai government, or district governments. Additionally, 12 secondary public general hospitals were selected from 4 districts (A-D) (areas). Because District A is the largest district in Shanghai and comprises both urban and rural areas covering approximately 20% of the total Shanghai population, 6 secondary public general hospitals were selected in this district. In the other 3 districts in Shanghai, 2 secondary public general hospitals were selected.

We recruited a sample of 18 tertiary hospitals and 36 secondary hospitals from 10 areas in 3 regions of China.

Data sources

For each selected hospital, general data (i.e., hospital level, number of beds, number of physicians, and number of nurses) were collected, and anonymous, paper-based, self-administered employee questionnaires were distributed and collected by trained coordinators in the surveyed hospitals or regions according to our study design.

In the employee survey, we randomly sampled 10% of the managers and administrative staff (at least 15), 10% of the frontline physicians (at least 15), nurses (at least 15) and health technicians and staff working in medical auxiliary departments (at least 5) in each hospital. The term manager refers to a hospital or department director (including working departments and administrative offices), and the term administrative staff refers to non-managerial employees working in administrative offices related to patient safety and medical quality. Frontline workers are non-managerial employees who interact directly with patients. Employees working in internal medicine, surgery, obstetrics and gynecology, pediatrics, the ICU, the ED, anesthesiology and operating room (OR), clinical auxiliary departments, and other departments (including stomatology, dermatology, ENT, ophthalmology,

For peer review only

psychiatry, traditional Chinese medicine departments, administrative and supporting departments) were recruited. The term "clinical auxiliary departments" refers to laboratory departments, imaging departments, electrocardiograph departments, pathology departments, pharmacy departments, supply centers, etc.

This study was approved by the Institutional Review Board of the School of Public Health, Fudan University (IRB# 2014-03-0502). Written informed consent ecause our survey of employees was anonymous and had During the survey, anonymous, self-administered questionnaires were distributed and collected by trained coordinators in the surveyed hospitals or regions according to our study design. Sampled employees who were willing to participate in our study filled the questionnaires and returned them to the coordinators.

Survey Data Analysis

Psychometric analysis

We conducted item analyses and confirmatory factor analysis to test the reliability and validity of the PSCHO revised for the Chinese context. In the confirmatory factor analysis, we used PROC CALIS in SAS (SAS Institute Inc., Cary, NC) to form a structural equation model with the raw data. The study demonstrated that eleven dimensions had high internal consistency (Cronbach's α coefficients ranged from 0.77 to 0.93), but one dimension ("fear of blame and punishment") had a lower Cronbach's α coefficient (0.66). The overall scale had a high Cronbach's α coefficient (0.96). The standardized root mean square residual (SRMR) and the root mean square error of approximation (RMSEA) of the PSCHO were 0.049 and 0.058, respectively. The Bentler's comparative fit index, the Bentler-Bonett normed fit index and the non-normed index values were 0.913, 0.906, and 0.907, respectively. The adjusted goodness of fit (AGFI) was 0.83. However, the goodness of fit index (GFI) was 0.84, slightly lower than the criterion for this index (GFI >0.85). Overall,

For peer review only

the constructive validity of the PSCHO revised for the Chinese cultural context in this study was acceptable.[30-32]

Statistical analysis

We used the percentage of "problematic responses" (PPRs) to measure the patient safety climate. A rating of less than 3 for a positive statement or greater than 3 for a negative statement was identified as a problematic response. A lower PPR is indicative of a better perception of the safety climate. This scoring method identifies areas of non-uniformity in safety that are of potential concern and may benefit from interventions to improve the safety climate.[21,22,33-35]

We computed the PPR for each safety climate dimension with each item in the dimension weighted equally. We also calculated the average PPR for all questions in the survey as a summary statistic, which we referred to as the "overall safety climate." These percentages were calculated as the averages of all responses received. Comparisons among working departments and job types were calculated separately for respondents who indicated that they worked in any of the 9 types of working departments (internal medicine, surgery, obstetrics and gynecology, pediatrics, ICU, ED, anesthesiology and OR, clinical auxiliary departments (CADs) and others) and then for employees by different job type (frontline physicians, frontline nurses, medical technicians, managers and others).

A two-level random intercept model was used to examine how working department and job type affected each dimension and the overall patient safety climate, with the hospital as the level-2 cluster. All models were controlled for other respondent characteristics (gender, age, education, length of working years, and monthly income) and hospital characteristics (region, hospital level, bed size, and doctor-nurse ratio). When estimating the predicted values of the PPR for each dimension and overall by

working department and job type, mean values and proportions were used for continuous covariates and categorical covariates, respectively.

To test the appropriateness of using a two-level model to account for the nesting of individuals within hospitals, we first calculated and tested the intra-class correlation coefficient (ICC) of the empty model, which included no independent variables for each dimension and the overall PPR.[36] The results revealed significant differences in employees' perceptions of the patient safety climate among hospitals (P<0.0001).

To depict differences by job type (focusing on physicians with various professional titles and other employees) within selected working departments, we added the interaction of the working department and job type variables to the models. To provide an immediate visual summary of the relevant predictions, we applied a heat map that provided a two-dimensional representation of data using colors. We focused on physicians in the interaction analysis because they are the most important staff in the healthcare service, and we intended to investigate whether there were differences among chief physicians, attending physicians, residents or below and other personnel within a department.

Statistical analyses and graphics were generated using SAS 9.20, Excel 2007 and R software 3.3.3.

Results

Respondent Characteristics

In this study, 4,176 staff from 54 selected hospitals responded to the survey. The response rate and the valid response rate were 87.86% and 86.70%, respectively. Among the valid respondents, 55.67% worked in secondary hospitals, 50.81% worked in Shanghai hospitals, and 52.15% worked in large bed size hospitals (>800 beds). The percentages of respondents working in the internal medicine, surgery,

For peer review only

obstetrics and gynecology, pediatric, ICU, ED, anesthesiology, OR and CADs were 19.46%, 18.99%, 6.54%, 3.15%, 3.59%, 6.08%, 1.55%, 2.21% and 13.51%, respectively. According to job type, 38.66% were frontline physicians (including 10.32% associate chief physicians or chief physicians, 15.53% attending physicians, and 12.81% residents or below), 36.61% were frontline nurses, 9.28% were medical technicians and 2.87% were managers.

The respondents were predominantly female (66.31%) and older than 45 years (52.43%). Nearly 52% of the respondents had worked within their hospitals for 10 years or more (Appendix A).

Perceptions of the Safety Climate

In this multi-region study, the mean PPR of the overall safety climate among all 54 hospitals was 9.00%. The dimensions with the three highest PPRs were "fear of blame and punishment" (64.81%), "fear of shame" (20.42%) and "provisions of safe care" (16.31%), and the dimension with the lowest PPR was "collective learning" (2.10%) (Table 1).

Variations among Clinical Departments

After controlling for the hospital and other individual characteristics, the top four predicted PPRs for the overall safety climate were in the ED (9.63%, 95%CL: 8.29%, 10.98%), CADs (9.52%, 95%CL: 8.49%, 10.55%), pediatrics (9.46%, 95%CL: 7.70%, 11.21%) and the ICU (9.19%, 95%CL: 7.56%, 10.83%); the two lowest predicted PPRs were in the obstetrics and gynecology department (6.82%, 95%CL: 5.52%,8.11%) and anesthesiology and the OR (7.44%, 95%CL: 5.84%, 9.04%). Generally, staff in obstetrics and gynecology departments seemed to perceive patient safety climate more positively across almost all dimensions than those in many other working departments. In addition, the personnel in anesthesiology and ORs reported slightly lower PPRs for the dimensions of work-unit contribution to the safety climate. Although the PPRs for "fear of shame" were universally high across the 9

types of departments, the PPR was slightly higher among surgery departments (23.51%, 95%CL: 19.49%, 27.53%) (Table 2).

In addition, the PPR for "organizational resources for safety" was higher in pediatrics (14.41%, 95%CL: 9.85%, 18.97%) than in the departments of surgery, obstetrics and gynecology, anesthesiology and OR, CADs, and others; the PPRs for "psychological safety" were also higher in pediatrics (9.50%, 95%CL: 5.90%, 13.09%) and in CADs (8.95%, 95%CL: 6.86%, 11.05%) than in the departments of internal medicine, surgery, obstetrics and gynecology, anesthesiology and OR, and others. Finally, the PPR for "problem responsiveness" was higher in CADs (5.50%, 95%CL: 4.00%, 7.01%) than in the other working departments (Table 2).

Variations among Job Types

After adjusting for other personnel and hospital characteristics, the PPR reported by physicians was highest for the overall safety climate (10.19%, 95%CL: 9.52%, 10.86%), whereas the PPR among managers was the lowest (7.45%, 95%CL: 5.47%, 9.43%). The PPRs for "fear of shame" and "fear of blame and punishment" were generally high among staff with various job types (Table 3).

Frontline workers, especially physicians, perceived "organizational resources for safety" more negatively (12.25%, 95%CL: 10.48%, 14.02%) than managers and non-professionals and non-managers. Physicians reported higher PPR for "unit recognition and support for safety efforts" (9.78%, 95%CL: 8.33%, 11.22%) than nurses, managers and non-professionals and non-managers. The perceptions of the physicians of "psychological safety" (8.30%, 95%CL: 6.96%, 9.63%) and "problem responsiveness" (4.09%, 95%CL: 3.12%, 5.07%) were also worse than those of all other staff (Table 3, Appendix B). Additionally, the managers' responses to many dimensions seemed to be similar to those of frontline workers (Appendix C).

For peer review only

Variations within Working Departments among Physicians with Various Titles and Other Personnel

Figure 1 shows the variations of the predicted means of perception on each dimension and the overall patient safety climate among physicians with different titles and other personnel within each of the 9 departments. The results revealed that PPRs reported by various types of staff in pediatrics, ICU, and CAD differed across more dimensions.

For example, chief physicians and associate chief physicians in pediatrics responded more negatively than other physicians and personnel on "organizational resources for safety" (37.03%, 95%CL: 24.87%, 49.18%) and "unit recognition and support for safety efforts" (20.28%, 95%CL: 10.66%, 29.91%); chief physicians and associate chief physicians and residents or below reported higher PPRs for "fear of shame" (31.58%, 95%CL: 13.44% 49.71%; 26.76%, 95%CL: 13.26%, 40.26%, respectively). However, attending physicians and residents or below reported higher PPRs for "provision of safe care" (29.56%, 95%CL: 16.21%, 42.90%; 26.17%, 95%CL: 13.27%, 39.08%, respectively) (Figure 1).

In the ICU, attending physicians seemed to respond notably negatively across almost all dimensions, with PPRs above 10%. However, chief physicians and associate chief physicians' PPR for the "provision of safe care" was remarkably higher than that of other physicians and personnel (65.10%, 95%CL: 47.78%, 82.42%) (Figure 1).

In anesthesiology and the OR, chief physicians and associate chief physicians and residents or below reported higher PPRs for "fear of shame" than other physicians and personnel (32.43%, 95%CL: 14.86%, 50.00%; 37.35%, 95%CL: 22.06%, 52.63%, respectively). However, residents or below and other personnel reported higher PPRs for "fear of blame and punishment" than other physicians (78.23%,

For peer review only

95%CL: 60.42%, 96.03%; 68.60%, 95%Cl: 59.39%, 77.81%, respectively) (Figure 1).

In the CAD, PPRs of residents or below were slightly higher across many dimensions. In addition, chief physicians and associate chief physicians had more negative perceptions regarding “provision of safe care” (35.64%, 95%CL: 16.92%, 54.35%) (Figure 1).

Discussion

The Patient Safety Climate in Hospitals

Patient safety climate is a core determinant that ensures and promotes hospital safety in China. The Chinese Hospital Association (CHA) has promulgated annual patient safety goals since 2007, and the National Health and Family Planning Commission (previously known as the Ministry of Health) established a web-based voluntary adverse event reporting system in 2008. Additionally, many provincial governments have actively facilitated the use of clinical pathways, disease management programs, and computer-assisted quality and safety programs; they have also linked government subsidies for public hospitals to the quality and safety of their medical care.[37,38] These efforts all aim to facilitate an improved patient safety climate in China’s hospitals.

The survey results revealed that the perception of overall patient climate was relatively good (PPR=9%). However, substantial attention should be paid to the dimensions “fear of blame and punishment” (65%), “fear of shame” (20%), “provision of safe care” (16%) and “organizational resources for safety” (10%) according to HRO theories.[39] The high prevalence of “fear of blame and punishment” and “fear of shame” among staff with different job types and in various working departments may be attributed to inappropriate systems of performance assessments and rewards, a hierarchical management style, difficult doctor-patient

For peer review only

relationships and the quintessentially Chinese notion of "face." Because a hierarchical management style and the quintessential notion of "face" are common in Chinese culture, high PPRs for "fear of blame and punishment" and "fear of shame" may exist in other hospitals in which the dominant Chinese culture is present. However, we also found that the PPRs of these two dimensions were significantly lower than those in the previous study in Pudong New Area (78.53% and 41.16%).[22] It is possible that an increasing number of policymakers, hospital managers and health professionals have gradually realized that concealing mistakes or errors might result in worse consequences and have implemented measures such as improving reward systems, changing the patient safety climate, and establishing information systems for easier error reporting to encourage the reporting of errors or mistakes.[40,41]

General Safety Climate Variations by Department

The results highlighted significant differences in perceptions of the patient safety climate among working departments. We found that personnel in pediatrics, the ICU, the ED, and CADs perceived lower levels of safety climate than personnel in the other working departments. Staff in the obstetrics and gynecology departments responded more positively both across many dimensions of safety climate and overall. Staff in anesthesiology and ORs perceived relatively low PPRs for the dimensions of work-unit contribution to the safety climate. These results are similar to those of previous studies showing that more resources may have been delivered and greater efforts made to overcome safety hazards in the anesthesiology department and Ors.[15,16] However, this survey also found that in anesthesiology and the OR, chief physicians, associate chief physicians, and residents perceived significantly high PPRs for "fear of shame," and residents and other personnel gave remarkably high PPRs for "fear of blame and punishment". Furthermore, a notably high percentage of personnel in surgery departments problematically perceived the dimension of "fear of shame". Generally, surgeons, who are physicians with high professional titles, have greater social identity and self-identity. However, medical

For peer review only - http://bmjopen.bmj.com/site/about/guidelines.xhtml

errors and/or accidents occurring in surgery, anesthesiology and the OR are usually more severe than those in other working departments. Therefore, staff in these departments will experience more pressure and face more serious consequences.

General Safety Climate Variations by Job Type

The results showed that physicians' PPRs were systematically higher than those of other staff in this study, whereas Western studies have shown the opposite.[13,16,19,42] Most frontline physicians in China are full-time employees of public hospitals. They hold dominant positions and are close to patients through the process of medical services. They are also responsible for ensuring patient safety and quality. If adverse events or healthcare errors happen, patients are more likely to complain and blame their physicians. Accordingly, frontline physicians might pay more attention to quality and safety measures and systems, and they also might obtain more firsthand experience or information about safety hazards.

This finding indicates that hospital managers and unit directors should establish and continuously improve internal managerial systems to encourage frontline physicians to report or share their experiences and information on patient safety and healthcare quality and to facilitate their proactive participation in related projects for improvement. Measures to appoint frontline physicians to serve as unit quality controllers, to encourage them to lead interdisciplinary groups to implement quality control circle activities, and to involve indicators of patient safety and quality improvement in individual performance assessments, etc., have been implemented in some hospitals in China and should be generalized.[43-45]

Besides, the results also revealed that managers' perceptions were relatively consistent with those of frontline workers on many dimensions but differed from those reported in many previous studies.[22,24,34,42] This finding might be partly attributed to managers' efforts and interventions to promote "speaking up" and

For peer review only

"communicating down" in China's public hospitals, including activities such as safety culture-oriented simulation training, Leadership WalkRounds, and efforts to engage frontline workers and managers in open discussion about safety events.[46,47] It is also possible, however, that these results were driven by the extent to which the managers in this survey involved working departments directors, in contrast to our 2013 study in Shanghai.[22] Working departments' directors in China are physicians who also provide healthcare for patients on the front lines.

Pediatrics Departments Should Receive More Attention

In this investigation, pediatrics personnel responded with higher PPRs in several dimensions of the safety climate, especially "organizational resources for safety" and "psychological safety." This is an alarming finding but is consistent with the increasingly severe shortage of pediatricians in China reported in some articles and news sources.[48-50] Statistical analyses have shown that the number of pediatricians in China increased by only 5,000 from 1995-2010,[51] and the current number of pediatric doctors per 1,000 children is only 0.5, one-third of the ratio in the US.[52] This issue will become more problematic following the implementation of China's Two-Child policy. Pediatricians continue to leave medical practice, especially in primary healthcare institutions, and many new medical graduates are unwilling to join.[53] The survey revealed that chief physicians and associate chief pediatricians were more worried about organizational resources for safety; attending physicians and residents with patient contact reported higher PPRs for "provision of safe care." The human-resources shortage in healthcare would be very likely to have a negative impact on health care quality and patient safety.

ICUs and EDs Should Receive More Attention

Working in ICUs and EDs is associated with a higher level of risk, complexity, and difficulty, a faster pace and lower predictability.[15,16] These work areas are intrinsically hazardous, and personnel working in these areas are prone to encounter

For peer review only

a high workload and pressure that can result in burnout.[54-57] Previous studies have indicated that burnout among healthcare providers may lead to reduced patient safety.[58-60] Nahrgang et al.[61] generally argued that burned-out employees' mental and physical energy levels decrease safe work behaviors and thus increase the likelihood of errors and work-related injuries. Flinn et al. conducted a post-call assessment of junior doctors after extended work shifts (average 32.75 h) and noted a significant decline in cognitive functioning and clinical decision-making performance.[62] Sharpe et al.[63] showed deteriorating performance of ICU residents during 26 h of continuous wakefulness. The results of this survey suggest that more attention should be paid to ICUs and EDs, whose predicted PPRs for the overall safety climate ranked in the top four among the working departments. Because of the intrinsic characteristics of ICUs and EDs, it is very important and practical to design and optimize systems to protect ICU and ED patients from preventable harm. This approach requires a balanced interdisciplinary effort directed at process characteristics and the execution of several measures in parallel.[64] Recent examples such as standardization of processes,[65-67] adaptation to humans' cognitive limitations,[68] optimization of working conditions,[69] and increasing use of supporting information technologies should be explored.

We also found that attending ICU physicians responded more negatively; almost all PPRs were above 10%. These health care workers are important personnel on the front lines in their departments and perhaps experience more safety problems.[43] Hospital managers and policy makers should carefully consider their opinions.

CADs Need Attention

Another meaningful finding of our study was that perceptions of patient safety climate among staff in CADs were worse than in many other working departments, especially for "psychological safety" and "problem responsiveness." Residents in the CADs were more worried about several dimensions, such as "overall emphasis on patient safety," "unit safety norms," "unit recognition and support for safety efforts,"

For peer review only - http://bmjopen.bmj.com/site/about/guidelines.xhtml

"collective learning," "psychological safety" and "problem responsiveness." Hospital managers might not pay sufficient attention to CADs compared with other working departments, especially with respect to the residents' training and motivation in this type of department.

Limitations

First, the data were based on self-reporting, which might involve recall/report bias. Second, because this study was a cross-sectional study, we could not rule out the potential for omitted variables. Although our analyses represent an important advance over prior studies because we adjusted for important known individual and hospital characteristics, unmeasured characteristics could play a role in distinguishing personnel by working department and job type. Third, the results from the 54 public general hospitals in three regions might not be generalizable to all hospitals in China, although our sample size was reasonably large and represented public hospitals in high, middle, and low socioeconomic level regions and the eastern, central, and western regions of China.

Conclusions

Our study highlights differences in perceptions of patient safety climate among and within working departments and job types that have not been previously documented in China. The findings indicate that safety climate improvement efforts should involve greater attention to the climate in the ICU, the ED, pediatrics, and CADs. Therefore, it would be effective and reasonable to implement specific measures to improve patient safety climate according to targeted departments and personnel.

Acknowledgments

We gratefully acknowledge the significant contributions of the following members of the research project team: Hongbo Zhu, Xuefeng Wei, and Jianjun Gu. The authors also acknowledge all hospitals that provided assistance with data collection

For peer review only

in this research project. We truly appreciate Dr. Yongfu Yu of the Department of Clinical Epidemiology at Aarhus University Hospital Olof Palmes, who provided us with important guidance on the statistical analysis.

Contributors

Di Xue conceived and designed the study. Di Xue, Fei Bai, Mingqi Li, Huiqing Tang, Jie Bai, and Ping Zhou performed the investigation. Ping Zhou and Mingqi Li conducted statistical analyses and interpreted the data. Ping Zhou drafted and revised the manuscript. Di Xue revised the manuscript. All authors read and approved the final manuscript.

Competing interests

The authors have declared that no competing interest exists.

Funding

This research project was supported by the National Natural Science Foundation of China, grant number 71473047.

Ethics approval

The study was approved by the Institutional Review Board of the School of Public Health, Fudan University (IRB# 2014-03-0502).

Data sharing statement

No additional data are available.

For peer review only

References

1 Cheng YM, Liu Y, Liu YM. Review on research and application of adverse events reporting system in China. *Chin Hosp Manage* 2012;32:40–2.

2 Hu R. WHO release of the Chinese version of the patient safety curriculum guide: multi-professional edition. *Chin Community Physician* 2012;29:23.

3 Zhu J, Li L, Li Y, et al. What constitutes patient safety culture in Chinese hospitals? *Int J Qual Health Care* 2012;24:250-7. doi: 10.1093/intqhc/mzs010.

4 Xu SX, National Health and Family Planning Commission of PR. [Seven measures to improve medical quality and patient safety]. China Hospital CEO 25 Dec 2015. http://www.h-ceo.com/article/read/6978/6978.html (accessed 7 Aug 2016).

5 Braithwaite J, Westbrook MT, Travaglia JF, et al. Cultural and associated enablers of, and barriers to, adverse incident reporting. *Qual Saf Health Care* 2010;19:229–33. doi: 10.1136/qshc.2008.030213.

6 Singer S, Lin S, Falwell A, et al. Relationship of safety climate and safety performance in hospitals. *Health Serv Res* 2009;44:399–421. doi: 10.1111/j.1475-6773.2008.00918.x.

7 Mardon RE, Khanna K, Sorra J, et al. Exploring relationships between Hospital patient safety culture and adverse events. *J Patient Saf* 2010;6:226–32. doi: 10.1097/PTS.0b013e3181fd1a00.

8 Huang DT, Clermont G, Kong L, et al. Intensive care unit safety culture and outcomes: a US multicenter study. *Int J Qual Health Care* 2010;22:151-61.

9 Hansen LO, Williams MV, Singer SJ. Perceptions of hospital safety climate and incidence of readmission. *Health Serv Res* 2011;46:596–616. doi: 10.1111/j.1475-6773.2010.01204.x.

10 Rose JS, Thomas CS, Tersigni A, et al. A leadership framework for culture change in health care. *Jt Comm J Qual Patient Saf* 2006;32:433– 42.

11 Flin R, Burns C, Mearns K, et al. Measuring safety climate in health care. *Qual Saf Health Care* 2006;15:109–15. doi: 10.1136/qshc.2005.014761.

12 Singer SJ, Vogus TJ. Safety climate research: taking stock and looking forward. *BMJ Qual Saf* 2013;22:1–4. doi: 10.1136/bmjqs-2012-001572.

13 Makary MA, Sexton JB, Freischlag JA, et al. Operating room teamwork among physicians and nurses: teamwork in the eye of the beholder. *J Am Coll Surg* 2006;202:746 –52. doi: 10.1016/j.jamcollsurg.2006.01.017.

14 Cooper JB, Blum RH, Carroll JS, et al. Differences in safety climate among hospital anesthesia departments and the effect of a realistic simulation-based training program. *Anesth Analg* 2008;106:574–84. doi: 10.1213/01.ane.0000296462.39953.d3.

15 Huang DT, Clermont G, Sexton JB, et al. Perceptions of safety culture vary across the intensive care units of a single institution. *Crit Care Med* 2007;35:165–76. doi: 10.1097/01.CCM.0000251505.76026.CF.

For peer review only

16 Singer SJ, Gaba DM, Falwell A, et al. Patient safety climate in 92 US hospitals: differences by work area and discipline. *Med Care* 2009;47:23-31. doi: 10.1097/MLR.0b013e31817e189d.

17 Paine LA, Rosenstein BJ, Sexton JB, et al. Assessing and improving safety culture throughout an academic medical centre: a prospective cohort study. *Qual Saf Health Care* 2010;19:547-54. doi: 10.1136/qshc.2009.039347.

18 Gehring K, Mascherek AC, Bezzola P, et al. Safety climate in Swiss hospital units: Swiss version of the Safety Climate Survey. *J Eval Clin Practice* 2015;21:332–8. doi: 10.1111/jep.12326.

19 Sullivan A, Camargo C, Cleary P, et al. Do emergency physicians and nurses differently perceive safety-related factors? The National ED Safety Study. *Acad Emerg Med* 2007;14:S210–1. doi: 10.1197/j.aem.2007.03.1310.

20 Sexton JB, Holzmueller CG, Pronovost PJ, et al. Variation in caregiver perceptions of teamwork climate in labor and delivery units. *J Perinatol* 2006;26:463– 70. doi: 10.1038/sj.jp.7211556.

21 Thomas EJ, Sexton JB, Helmreich RL. Discrepant attitudes about teamwork among critical care nurses and physicians. *Crit Care Med* 2003;31:956–9. doi: 10.1097/01.CCM.0000056183.89175.76.

22 Zhou P, Bundorf MK, Gu J, et al. Survey on patient safety climate in public hospitals in China. *BMC Health Serv Res* 2015;15:53. doi: 10.1186/s12913-015-0710-x.

For peer review only

23 Singer S, Meterko M, Baker L, et al. Workforce perceptions of hospital safety culture: development and validation of the patient safety climate in healthcare organizations survey. *Health Serv Res* 2007;42:1999–2021. doi: 10.1111/j.1475-6773.2007.00706.x.

24 Hartmann CW, Rosen AK, Meterko M, et al. An overview of patient safety climate in the VA. *Health Serv Res* 2008;43:1263–84. doi: 10.1111/j.1475-6773.2008.00839.x.

25 Singer SJ, Hartmann CW, Hanchate A, et al. Comparing safety climate between two populations of hospitals in the United States. *Health Serv Res* 2009;44:1563–83. doi: 10.1111/j.1475-6773.2009.00994.x.

26 Colla JB, Bracken AC, Kinney LM, et al. Measuring patient safety climate: a review of surveys. *Qual Saf Health Care* 2005;14:364-6.

27 Weick KE, Sutcliffe KM, 2001. Managing the Unexpected. San Francisco, CA: Jossey-Bass.

28 Nie Y, Mao X, Cui H, et al. Hospital survey on patient safety culture in China. *BMC Health Serv Res* 2013;13:228.

29 Chen IC, Li HH. Measuring patient safety culture in Taiwan using the Hospital Survey on Patient Safety Culture (HSOPSC). *BMC Health Serv Res* 2010;10:152.

30 Bentler PM, Bonett DG. Significance tests and goodness of fit in the analysis of covariance structures. *Psychol Bull* 1980;88:588–606.

For peer review only

31 McDonald RP, Ho MHR. Principles and practice in reporting structural Equation Analyses. *Psychol Methods* 2002;7:64–82.

32 Gefen D, Straub DW, Boudreau MC. Structural equation modeling and regression: guidelines for research practice. *Commun Assoc Inform Syst* 2000;4:1-77.

33 Gaba DM, Howard SK, Jump B. Production pressure in the work environment. California anesthesiologists' attitudes and experiences. *Anesthesiology* 1994;81:488–500. doi: 10.1097/00000542-199408000-00028.

34 Singer SJ, Gaba DM, Geppert JJ, et al. The culture of safety: results of an organization-wide survey in 15 California hospitals. *Qual Saf Health Care* 2003;12:112–8. doi: 10.1097/MLR.0b013e31817925c1.

35 Singer SJ, Falwell A, Gaba DM, et al. Patient safety climate in US hospitals: variation by management Level. *Med Care* 2008;46:1149–56.

36 Wang JC, Xie HY, Jiang BF, 2010. Multilevel Models: Methods and Applications. Beijing: Higher Education Press.

37 Gong TX, 2012. Impact of Financial Compensation Policy: an Intervention Study in 15 Hospitals of Traditional Chinese Medicine. Beijing: Beijing University of Chinese Medicine.

38 Zhou X, Wu EZ. The practice and discussion on performance appraisal reform in county-level public hospitals in Haining City. *J Health Econ Res* 2015;8:24-6, 27.

39 Gaba DM, Singer SJ, Sinaiko AD, et al. Differences in safety climate between Hospital personnel and Naval aviators. *Hum Factors* 2003;45:173–85. doi: 10.1518/hfes.45.2.175.27238.

40 Chiang HY, Hsiao YC, Lin SY, et al. Incident reporting culture: scale development with validation and reliability and assessment of hospital nurses in Taiwan. *Int J Qual Health Care* 2011;23:429-36.

41 Sanghera IS, Franklin BD, Dhillon S. The attitudes and beliefs of healthcare professionals on the causes and reporting of medication errors in a UK intensive care unit. *Anaesthesia* 2007;62:53-61.

42 Pronovost PJ, Weast B, Holzmueller CG, et al. Evaluation of the culture of safety: survey of clinicians and managers in an academic medical center. *Qual Saf Health Care* 2003;12:405– 10.

43 He XY, Gu JJ, Sun X, et al. Medical quality control system in public general hospitals in Pudong New Area. *Chin Health Qual Manag* 2015;22:25-8.

44 Wang YQ, Qin XY, Gao X, et al. Quality control circle in the continuous quality improvement campaign of the hospital. *Chin J Hosp Admin* 2014;30:425-7.

45 Liu SJ, Xu MY. The practice of the assessment of medical quality in clinical departments in a general hospital. *Chin Hosp Manag* 2013;33:56-7.

46 Wang LH, Zhao GG, Yang X, et al. On the practice and insights of building a safety-oriented culture in the hospital. *Chin J Hosp Admin* 2009;25:595-8.

For peer review only

47 Liu HS. Reinforcing internal communication to promote the harmonious development of the hospital. *China Science and Technology Review* 2009;26:102.

48 Ren YQ, Lu MH, Zhang Y, et al. Issues and solutions of building a pediatrician pool in Shanghai. *Hospital Administration Journal of Chinese PLA* 2016;23:228-30.

49 Liu SH, Pi GH, Kang Q, et al. Current situation of pediatric in Nanchong of Sichuan Province. *Journal North Sichuan Medical College* 2015;30:109-11.

50 *Xin Hua News.* China faces shortage of pediatricians. 2014. http://news.xinhuanet.com/english/health/2014-09/11/c_133636236.htm (accessed 15 Sept 2016).

51 Zhao CY. China faces critical pediatrician shortage. *China Daily,* 2011. http://www.chinadaily.com.cn/china/2011-02/17/content_12034787.htm (accessed 10 Sept 2016).

52 China Network Television (CNTV). China facing pediatrician shortage. 2016. http://www.china.org.cn/video/2016-01/20/content_37620967.htm (accessed 10 Sept 2016).

53 Pan SJ, Chen JM, Bai JG. Analysis and countermeasures on the causes of pediatric human resources shortage--Take Shanxi Province as an example. *Soft Sci Health* 2016;30:31-4.

For peer review only

54 Lin PH, Xiao HX, Lin XQ. Survey on stress management of emergency physicians and its countermeasures in Fujian Province. *Chin Rural Health Serv Admin* 2014;34:503-6.

55 Wang L, Liu ZF, Peng XW, et al. Present situation investigation and analysis of emergency departments of 20 tertiary general hospitals in Heilongjiang Province. *Management* 2015;35:68-9.

56 Sheng WJ, Zhang S, Zhang L. Analysis on ICU healthcare providers' stress resources and its countermeasures. *Chronic Pathematology Journal* 2010;12:1674-5.

57 Li C, Tian XH, Feng LN, et al. Study on correlation between job stress and coping styles and job burnout of ICU nurses (in Chinese). *Chin Nurs Res* 2014;28:3236-7.

58 Teng CI, Shyu YI, Chiou WK, et al. Interactive effects of nurse-experienced time pressure and burnout on patient safety: a cross-sectional survey. *Int J Nurs Stud* 2010;47:1442–50. doi: 10.1016/j.ijnurstu.2010.04.005.

59 West CP, Tan AD, Habermann TM, et al. Association of resident fatigue and distress with perceived medical errors. *JAMA* 2009;302:1294–300. doi: 10.1001/jama.2009.1389.

60 Halbesleben JR, Rathert C. Linking physician burnout and patient outcomes: exploring the dyadic relationship between physicians and patients. *Health Care Manage Rev* 2008;33:29–39. doi: 10.1097/01.HMR.0000304493.87898.72.

For peer review only

61 Nahrgang JD, Morgeson FP, Hofmann DA. Safety at work: a meta-analytic investigation of the link between job demands, job resources, burnout, engagement, and safety outcomes. *J Appl Psychol* 2011;96:71–94. doi: 10.1037/a0021484.

62 Flinn F, Armstrong C. Junior doctors' extended work hours and the effects on their performance: the Irish case. *Int J Qual HealthCare* 2011;23:210–7. doi: 10.1093/intqhc/mzq088.

63 Sharpe R, Koval V, Ronco JJ, et al. The impact of prolonged continuous wakefulness on resident clinical performance in the intensive care unit: a patient simulator study. *Crit Care Med* 2010;38:766–70. doi: 10.1097/CCM.0b013e3181cd122a.

64 Valentin A. Approaches to decreasing medication and other care errors in the ICU. *Curr Opin Crit Care* 2013;19:474-9. doi: 10.1097/MCC.0b013e328364d4f9.

65 Dehmel C, Braune SA, Kreymann G, et al. Do centrally pre-prepared solutions achieve more reliable drug concentrations than solutions prepared on the ward? *Intensive Care Med* 2011;37:1311–6.

66 Poon EG, Keohane CA, Yoon CS, et al. Effect of bar-code technology on the safety of medication administration. *N Engl J Med* 2010;362:1698–707.

67 Askari M, Eslami S, Louws M, et al. Frequency and nature of drug-drug interactions in the intensive care unit. *Pharmacoepidemiol Drug Saf* 2013;22:430–7.

For peer review only

68 Garrouste-Orgeas M, Soufir L, Tabah A, et al. A multifaceted program for improving quality of care in intensive care units: IATROREF study. *Crit Care Med* 2012;40:468–76.

69 Wallace DJ, Angus DC, Barnato AE, et al. Nighttime intensivist staffing and mortality among critically ill patients. *N Engl J Med* 2012;366:2093–101.

For peer review only

Tables and captions

Table 1 Problematic Responses for Dimensions and Items

Table 2 Patient Safety Climate by Working Department

Table 3 Patient Safety Climate by Job Type

Table 1 Problematic Responses for Dimensions and Items

Dimensions and Items †	% Problematic
Hospital contributions to the safety climate	
Senior managers' engagement	2.84
Organizational resources for safety	9.55
Overall emphasis on patient safety	2.63
Work-unit contributions to the safety climate	
Unit managers' support	5.42
Unit safety norms	4.51
Unit recognition and support for safety efforts	6.68
Collective learning	2.10
Psychological safety	5.68
Problem responsiveness	2.61
Interpersonal contributions to the safety climate	
Fear of shame	20.42
Fear of blame and punishment	64.81
Other aspects of the safety climate	
Provision of safe care	16.31
Overall average	9.00

† Means of all items in a dimension were averaged to calculate the dimension mean.

For peer review only

Table 2 Patient Safety Climate by Working Department †

Dimensions	Internal Medicine		Surgery		Obstetrics & Gynecology	
	Estimate	95% CL	Estimate	95% CL	Estimate	95% CL
Hospital contributions to the safety climate						
Senior managers’ engagement	2.74	[1.58,3.90]	2.06	[0.87,3.25]	1.80	[0.21,3.39]
Organizational resources for safety	9.38	[6.90,11.86]	7.54	[5.00,10.09]	7.02	[3.65,10.40]
Overall emphasis on patient safety	2.96	[1.57,4.35]	2.19	[0.76,3.62]	1.56	[-0.38,3.49]
Work-unit contributions to the safety climate						
Unit managers' support	5.21	[3.40,7.01]	4.90	[3.05,6.76]	3.15	[0.65,5.64]
Unit safety norms	3.85	[2.67,5.03]	3.26	[2.05,4.47]	3.92	[2.29,5.55]
Unit recognition and support for safety efforts	5.95	[3.95,7.94]	5.51	[3.46,7.56]	4.29	[1.59,6.99]
Collective learning	2.14	[1.10,3.19]	1.40	[0.33,2.48]	0.91	[-0.53,2.35]
Psychological safety	2.87	[0.94,4.79]	1.83	[-0.15,3.80]	0.72	[-1.92,3.37]
Problem responsiveness	0.93	[-0.46,2.31]	0.42	[-1.00,1.84]	-0.40	[-2.30,1.49]
Interpersonal contributions to the safety climate						
Fear of shame	19.77	[15.84,23.69]	23.51	[19.49,27.53]	12.46	[7.26,17.67]
Fear of blame and punishment	66.45	[61.69,71.21]	69.93	[65.06,74.79]	58.80	[52.60,65.01]
Other aspects of the safety climate						
Provision of safe care	17.19	[13.45,20.93]	17.67	[13.84,21.50]	22.72	[17.75,27.70]
Overall	8.51	[7.57,9.46]	8.28	[7.30,9.25]	6.82	[5.52,8.11]

Continued:

Dimensions	Pediatrics		ICU		ED	
	Estimate	95% CL	Estimate	95% CL	Estimate	95% CL
Hospital contributions to the safety climate						
Senior managers' engagement	2.19	[0.02,4.35]	4.86	[2.84,6.87]	4.62	[2.97,6.28]
Organizational resources for safety	14.41	[9.85,18.97]	10.54	[6.28,14.79]	8.83	[5.32,12.35]
Overall emphasis on patient safety	2.60	[-0.04,5.24]	2.79	[0.33,5.26]	2.71	[0.70,4.73]
Work-unit contributions to the safety climate						
Unit managers' support	4.42	[1.02,7.82]	5.44	[2.27,8.62]	6.29	[3.69,8.89]
Unit safety norms	4.71	[2.50,6.92]	3.72	[1.66,5.79]	5.43	[3.73,7.12]
Unit recognition and support for safety efforts	8.57	[4.93,12.20]	5.10	[1.71,8.48]	8.33	[5.52,11.13]
Collective learning	3.17	[1.22,5.13]	2.54	[0.71,4.36]	2.60	[1.11,4.10]
Psychological safety	9.50	[5.90,13.09]	6.69	[3.34,10.04]	5.29	[2.54,8.05]
Problem responsiveness	0.75	[-1.81,3.32]	1.11	[-1.29,3.50]	2.02	[0.05,4.00]
Interpersonal contributions to the safety climate						
Fear of shame	16.95	[10.01,23.88]	20.25	[13.78,26.71]	20.09	[14.68,25.50]
Fear of blame and punishment	61.47	[53.27,69.67]	60.06	[52.40,67.71]	65.68	[59.24,72.13]
Other aspects of the safety climate						
Provision of safe care	19.48	[12.84,26.12]	22.22	[16.03,28.41]	19.94	[14.77,25.10]
Overall	9.46	[7.70,11.21]	9.19	[7.56,10.83]	9.63	[8.29,10.98]

Continued:

Dimensions	Anesthesiology & OR		CAD		Others	
	Estimate	95% CL	Estimate	95% CL	Estimate	95% CL
Hospital contributions to the safety climate						
Senior managers' engagement	1.87	[-0.11,3.84]	1.53	[0.27,2.80]	1.89	[0.85,2.94]
Organizational resources for safety	6.10	[1.93,10.28]	7.12	[4.43,9.81]	7.80	[5.55,10.05]
Overall emphasis on patient safety	3.26	[0.85,5.68]	2.44	[0.93,3.96]	1.87	[0.63,3.12]
Work-unit contributions to the safety climate						
Unit managers' support	2.85	0.26,5.96]	5.80	[3.84,7.77]	4.29	[2.67,5.91]
Unit safety norms	2.36	[0.34,4.38]	6.12	[4.83,7.40]	3.99	[2.93,5.06]
Unit recognition and support for safety efforts	3.03	[-0.30,6.36]	8.97	[6.80,11.13]	5.34	[3.52,7.15]
Collective learning	0.44	[-1.35,2.23]	3.20	[2.06,4.34]	0.91	[-0.03,1.85]
Psychological safety	2.28	[-1.01,5.56]	8.95	[6.86,11.05]	3.05	[1.32,4.78]
Problem responsiveness	0.43	[-1.92,2.78]	5.50	[4.00,7.01]	0.26	[-0.99,1.51]
Interpersonal contributions to the safety climate						
Fear of shame	21.80	[15.44,28.15]	16.22	[11.99,20.45]	15.60	[11.99,19.20]
Fear of blame and punishment	66.92	[59.39,74.45]	68.73	[63.63,73.83]	67.12	[62.72,71.53]
Other aspects of the safety climate						
Provision of safe care	19.52	[13.44,25.60]	16.54	[12.51,20.58]	18.94	[15.51,22.37]
Overall	7.44	[5.84,9.04]	9.52	[8.49,10.55]	7.79	[6.93,8.65]

† The results reflect predicted means (estimate and 95% 95% CL) by clinical departments based on 2-level random intercept models for each dimension and the overall safety climate adjusted for other individual characteristics (age, gender, education, working years and monthly income) and hospital characteristics (tertiary level vs secondary level, hospital size, hospital location, and doctor-nurse ratio). When estimating the predicted mean values of the PPR for each dimension and overall by clinical department and job type, we held other covariates constant at their means.

Table 3 Patient Safety Climate by Job Type †

Dimensions	Physicians		Nurses		Medical technicians	
	Estimate	95% CL	Estimate	95% CL	Estimate	95% CL
Hospital contributions to the safety climate						
Senior managers' engagement	4.01	[3.20,4.81]	2.17	[1.28,3.07]	2.76	[0.95,4.58]
Organizational resources for safety	12.25	[10.48,14.02]	9.97	[8.02,11.92]	8.86	[5.04,12.69]
Overall emphasis on patient safety	3.36	[2.43,4.29]	2.37	[1.34,3.41]	1.83	[-0.38,4.04]
Work-unit contributions to the safety climate						
Unit managers' support	6.15	[4.92,7.38]	5.50	[4.13,6.86]	4.26	[1.41,7.10]
Unit safety norms	4.92	[4.11,5.74]	4.99	[4.09,5.90]	2.93	[1.07,4.78]
Unit recognition and support for safety efforts	9.78	[8.33,11.22]	5.45	[3.87,7.04]	6.19	[3.14,9.24]
Collective learning	2.86	[2.14,3.58]	1.80	[0.99,2.60]	0.75	[-0.89,2.39]
Psychological safety	8.30	[6.96,9.63]	5.84	[4.35,7.32]	3.90	[0.89,6.91]
Problem responsiveness	4.09	[3.12,5.07]	1.63	[0.56,2.71]	0.10	[-2.05,2.25]
Interpersonal contributions to the safety climate						
Fear of shame	18.39	[15.43,21.35]	19.76	[16.56,22.97]	17.48	[11.64,23.33]
Fear of blame and punishment	65.04	[61.35,68.73]	63.07	[59.10,67.03]	62.67	[55.72,69.61]
Other aspects of the safety climate						
Provision of safe care	17.57	[14.76,20.37]	21.07	[18.03,24.11]	18.98	[13.39,24.58]
Overall	10.19	[9.52,10.86]	8.83	[8.09,9.57]	7.89	[6.42,9.36]

Continued:

Dimensions	Managers ‡		Non-professionals and Non-managers	
	Estimate	95% CL	Estimate	95% CL
Hospital contributions to the safety climate				
Senior managers’ engagement	2.39	[-0.06,4.83]	1.76	[0.49,3.03]
Organizational resources for safety	5.71	[0.56,10.85]	6.96	[4.25,9.67]
Overall emphasis on patient safety	3.12	[0.11,6.13]	1.76	[0.24,3.28]
Work-unit contributions to the safety climate				
Unit managers' support	4.38	[0.52,8.24]	3.25	[1.28,5.22]
Unit safety norms	3.36	[0.85,5.86]	4.56	[3.27,5.85]
Unit recognition and support for safety efforts	4.70	[0.60,8.79]	4.48	[2.30,6.65]
Collective learning	1.72	[-0.49,3.94]	2.49	[1.35,3.64]
Psychological safety	-0.41	[-4.48,3.65]	5.26	[3.15,7.36]
Problem responsiveness	-1.32	[-4.22,1.58]	1.61	[0.10,3.12]
Interpersonal contributions to the safety climate				
Fear of shame	18.95	[11.19,26.72]	17.99	[13.73,22.24]
Fear of blame and punishment	66.40	[57.25,75.54]	67.92	[62.78,73.06]
Other aspects of the safety climate				
Provision of safe care	20.67	[13.24,28.11]	18.50	[14.45,22.56]
Overall	7.45	[5.47,9.43]	8.21	[7.18,9.25]

† The results reflect predicted means (estimate and 95% 95% CL) for clinical departments based on 2-level random intercept models for each dimension and the overall safety climate adjusted for other individual characteristics (age, gender, education, working years, and monthly income) and hospital characteristics (tertiary level vs secondary level, hospital size, hospital location, and doctor-nurse ratio). When estimating the predicted mean values of the PPR for each dimension and overall by clinical department and job type, we held other covariates constant at their means.

‡ Including managers in the clinical departments, CADs, and administrative departments.

Figures and legends

Figure 1. Heatmap of predictive means of staff among 9 working departments across physicians with various titles and other staff on each dimension and the overall safety climate.

For peer review only

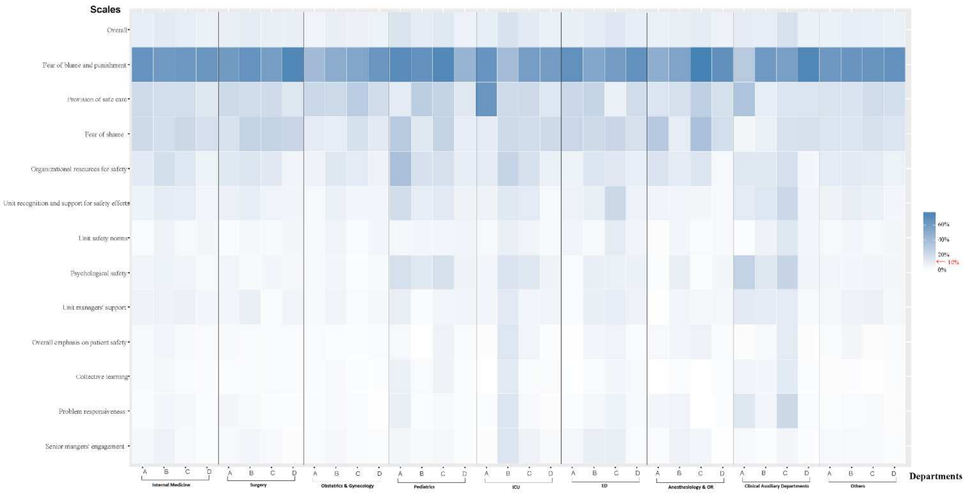

Figure 1. Heatmap of predictive means of staff among 9 working departments across physicians with various titles and other staff on each dimension and the overall safety climate

Note: A- chief physicians or associate chief physicians; B-attending physicians; C-residents or below; D-others

Figure 1. Heatmap of predictive means of staff among 9 working departments across physicians with various titles and other staff on each dimension and the overall safety climate.

1074x595mm (96 x 96 DPI)

Appendix A: General Respondent Information

Work Area Characteristics	N	%	Worker Characteristics	N	%
Region			Gender		
Shanghai	2094	50.81	Male	1365	33.69
Hubei Province	1095	26.57	Female	2687	66.31
Gansu Province	932	22.62	Total	4052	100.00
Total	4121	100.00	Age		
Hospital level			<25	269	6.67
Tertiary	1827	44.33	25-34	771	19.11
Secondary	2294	55.67	35-45	879	21.79
Total	4121	100.00	>=45	2115	52.43
Bed size (beds)			Total	4034	100.00
Small (≤500)	841	20.41	Education		
Middle (501-800)	1131	27.44	Master's degree or above	737	18.03
Large (>800)	2149	52.15	Bachelor's degree	2205	53.94
Total	4121	100.00	Associate's degree or below	1146	28.03
Affiliated with medical school			Total	4088	100.00
Yes	2644	65.08	Working years		
No	1419	34.92	<5	1073	26.63
Total	4063	100.00	5-10	852	21.15
Clinical department			>10	2104	52.22
Internal Medicine	791	19.46	Total	4029	100.00
Surgery	772	18.99	Monthly income (RMB)		
Obstetrics & Gynecology	266	6.54	<4000	1311	32.25
Pediatrics	128	3.15	4000-7999.99	1767	43.47
ICU	146	3.59	≥8000	987	24.28
Emergency Department (ED)	247	6.08	Total	4065	100.00
Anesthesiology	63	1.55	Job type		
Operating Room (OR)	90	2.21	Frontline physicians	1563	38.66
Clinical Auxiliary Departments (CAD)†	549	13.51	*(Associate) Chief physicians*	*417*	*10.32*
Others△	1013	24.92	*Attending physicians*	*628*	*15.53*
Total	4065	100.00	*Resident or below*	*518*	*12.81*
			Frontline nurses	1480	36.61
Doctor-nurse ratio			Medical technicians	375	9.28
Mean (SD)	0.81	0.59	Managers‡	116	2.87
Bed-Nurse ratio			Non-professionals and non-managers	509	12.58
Mean (SD)	2.53	2.52	Total	4043	100.00

† Referring to laboratory department, image department, ECG department, pathology department, pharmacy department, supply center, etc.

△Including stomatology, dermatology, ENT, ophthalmology, psychiatry, traditional Chinese medicine departments, and administrative offices.

‡ Including managers in the clinical departments, clinical auxiliary departments, and administrative departments.

For peer review only

Appendix B: Differences in Patient Safety Climate among Job Types (Pair-wise Comparison with Physicians) †

Dimensions	Nurses-Physicians		Medical Technicians-Physicians		Managers-Physicians		Others-Physicians	
	Estimate	95% CL	Estimate	95% CL	Estimate	95% CL	Estimate	95% CL
Hospital contributions to the safety climate								
Senior managers' engagement	-1.83	[-3.29,-0.38]	-1.24	[-3.71,1.23]	-1.62	[-4.82,1.58]	-2.25	[-3.96,-0.53]
Organizational resources for safety	-2.28	[-5.34,0.77]	-3.39	[-8.56,1.78]	-6.55	[-13.24,-0.15]	-5.30	[-8.89,-1.70]
Overall emphasis on patient safety	-0.98	[-2.78,0.81]	-1.53	[-4.59,1.53]	-0.24	[-4.20,3.72]	-1.60	[-3.71,0.51]
Work-unit contributions to the safety climate								
Unit managers' support	-0.65	[-2.95,1.65]	-1.89	[-5.80,2.02]	-1.76	[-6.83,3.30]	-2.90	[-5.6,-0.19]
Unit safety norms	0.07	[-1.43,1.56]	-2.00	[-4.53,0.53]	-1.57	[-4.85,1.71]	-0.36	[-2.12,1.39]
Unit recognition and support for safety efforts	-4.32	[-6.75,-1.90]	-3.59	[-7.68,0.51]	-5.08	[-10.39,-0.23]	-5.30	[-8.16,-2.44]
Collective learning	-1.06	[-2.38,0.26]	-2.11	[-4.35,0.13]	-1.13	[-4.04,1.77]	-0.36	[-1.92,1.19]
Psychological safety	-2.46	[-4.89,-0.04]	-4.40	[-8.51,-0.29]	-8.71	[-14.03,-3.39]	-3.04	[-5.89,-0.19]
Problem responsiveness	-2.46	[-4.19,-0.73]	-3.99	[-6.92,-1.07]	-5.41	[-9.20,-1.62]	-2.48	[-4.51,-0.45]
Interpersonal contributions to the safety climate								
Fear of shame	1.37	[-3.18,5.93]	-0.90	[-8.59,6.78]	0.57	[-9.39,10.52]	-0.40	[-5.78,4.97]
Fear of blame and punishment	-1.98	[-7.30,3.34]	-2.38	[-11.34,6.59]	1.35	[-10.26,12.96]	2.88	[-3.41,9.16]
Other aspects of the safety climate								
Provision of safe care	3.50	[-0.87,7.87]	1.42	[-5.96,8.80]	3.11	[-6.45,12.66]	0.94	[-4.22,6.09]
Overall	-1.36	[-2.53,-0.18]	-2.29	[-4.28,-0.3]	-2.74	[-5.32,-0.16]	-1.97	[-3.36,-0.59]

† Results reflect differences in predicted values (estimate and 95% 95% CL) among job types by pair-wise comparison with physicians, based on 2-level random intercept models for each dimension and the overall safety climate adjusted for other individual characteristics (age, gender, education, working years, and monthly income) and hospital characteristics (tertiary level vs secondary level, hospital size, hospital location, and doctor-nurse ratio). When estimating the predicted mean values of the PPR for each dimension and overall by clinical department and job type, we held other covariates constant at their means.

For peer review only

Appendix C: Differences in Patient Safety Climate among Job Types (Pair-wise Comparison with Managers) †

Dimensions	Physicians-Managers		Nurses-Managers		Medical Technicians -Managers		Others-Managers	
	Estimate	95% CL	Estimate	95% CL	Estimate	95% CL	Estimate	95% CL
Hospital contributions to the safety climate								
Senior managers' engagement	1.62	[-1.38,4.62]	-0.21	[-3.36,2.94]	0.38	[-2.47,3.23]	-0.63	[-3.74,2.49]
Organizational resources for safety	6.55	[0.28,12.82]	4.27	[-2.33,10.86]	3.16	[-2.81,9.12]	1.25	[-5.27,7.78]
Overall emphasis on patient safety	0.24	[-3.47,3.94]	-0.75	[-4.64,3.15]	-1.29	[-4.82,2.23]	-1.36	[-5.21,2.00]
Work-unit contributions to the safety climate								
Unit managers' support	1.76	[-2.98,6.51]	1.11	[-3.87,6.10]	-0.13	[-4.64,4.38]	-1.13	[-6.07,3.80]
Unit safety norms	1.57	[-1.50,4.64]	1.64	[-1.60,4.87]	-0.43	[-3.35,2.49]	1.21	[-1.99,4.40]
Unit recognition and support for safety efforts	5.08	[0.11,10.05]	0.76	[-4.47,5.99]	1.50	[-3.23,6.23]	-0.22	[-5.39,4.96]
Collective learning	1.13	[-1.58,3.85]	0.07	[-2.78,2.93]	-0.97	[-3.56,1.61]	0.77	[-2.05,3.60]
Psychological safety	8.71	[3.73,13.70]	6.25	[1.01,11.49]	4.31	[-0.43,9.06]	5.67	[0.48,10.85]
Problem responsiveness	5.41	[1.86,8.96]	2.95	[-0.78,6.68]	1.42	[-1.96,4.79]	2.93	[-0.76,6.62]
Interpersonal contributions to the safety climate								
Fear of shame	-0.57	[-9.89,8.76]	0.81	[-9.00,10.62]	-1.47	[-10.34,7.41]	-0.97	[-10.68,8.74]
Fear of blame and punishment	-1.35	[-12.23,9.52]	-3.33	[-14.78,8.11]	-3.73	[-14.08,6.63]	1.52	[-9.81,12.85]
Other aspects of the safety climate								
Provision of safe care	-3.11	[-12.05,5.84]	0.39	[-9.02,9.81]	-1.69	[-10.21,6.83]	-2.17	[-11.49,7.15]
Overall	2.74	[0.32,5.15]	1.38	[-1.16,3.92]	0.44	[-1.85,2.74]	0.76	[-1.75,3.28]

† Results reflect differences in predicted values (estimate and 95% 95% CL) among job types by pair-wise comparison with managers, based on 2-level random intercept models for each dimension and the overall safety climate adjusted for other individual characteristics (age, gender, education, working years, and monthly income) and hospital characteristics (tertiary level vs secondary level, hospital size, hospital location, and doctor-nurse ratio). When estimating the predicted mean values of the PPR for each dimension and overall by clinical department and job type, we held other covariates constant at their means.

For peer review only

STROBE Statement—checklist of items that should be included in reports of observational studies

	Item No	Recommendation	Our research
**Title and abstract**	1	(*a*) Indicate the study's design with a commonly used term in the title or the abstract	P1
		(*b*) Provide in the abstract an informative and balanced summary of what was done and what was found	P2
**Introduction**			
Background/rationale	2	Explain the scientific background and rationale for the investigation being reported	P4-5
Objectives	3	State specific objectives, including any prespecified hypotheses	P
**Methods**			
Study design	4	Present key elements of study design early in the paper	P5-10
Setting	5	Describe the setting, locations, and relevant dates, including periods of recruitment, exposure, follow-up, and data collection	P6-7
Participants	6	(*a*) *Cohort study*—Give the eligibility criteria, and the sources and methods of selection of participants. Describe methods of follow-up	-
		Case-control study—Give the eligibility criteria, and the sources and methods of case ascertainment and control selection. Give the rationale for the choice of cases and controls	_
		Cross-sectional study—Give the eligibility criteria, and the sources and methods of selection of participants	P7
		(*b*) *Cohort study*—For matched studies, give matching criteria and number of exposed and unexposed	_
		Case-control study—For matched studies, give matching criteria and the number of controls per case	_
Variables	7	Clearly define all outcomes, exposures, predictors, potential confounders, and effect modifiers. Give diagnostic criteria, if applicable	P9-10
Data sources/ measurement	8*	For each variable of interest, give sources of data and details of methods of assessment (measurement). Describe comparability of assessment methods if there is more than one group	P5-6
Bias	9	Describe any efforts to address potential sources of bias	P9
Study size	10	Explain how the study size was arrived at	P6-7
Quantitative variables	11	Explain how quantitative variables were handled in the analyses. If applicable, describe which groupings were chosen and why	P8-10
Statistical methods	12	(*a*) Describe all statistical methods, including those used to control for confounding	P8-10
		(*b*) Describe any methods used to examine subgroups and interactions	P8-10
		(*c*) Explain how missing data were addressed	-
		(*d*) *Cohort study*—If applicable, explain how loss to follow-up was addressed	_
		Case-control study—If applicable, explain how matching of cases and controls was addressed	_
		Cross-sectional study—If applicable, describe analytical methods	P8-10

For peer review only - http://bmjopen.bmj.com/site/about/guidelines.xhtml

taking account of sampling strategy	
(*e*) Describe any sensitivity analyses	_

Continued on next page

For peer review only

For peer review only - http://bmjopen.bmj.com/site/about/guidelines.xhtml

Results			Our research
Participants	13*	(a) Report numbers of individuals at each stage of study—eg numbers potentially eligible, examined for eligibility, confirmed eligible, included in the study, completing follow-up, and analysed	-
		(b) Give reasons for non-participation at each stage	-
		(c) Consider use of a flow diagram	-
Descriptive data	14*	(a) Give characteristics of study participants (eg demographic, clinical, social) and information on exposures and potential confounders	P10-11
		(b) Indicate number of participants with missing data for each variable of interest	-
		(c) *Cohort study*—Summarise follow-up time (eg, average and total amount)	-
Outcome data	15*	*Cohort study*—Report numbers of outcome events or summary measures over time	-
		Case-control study—Report numbers in each exposure category, or summary measures of exposure	-
		Cross-sectional study—Report numbers of outcome events or summary measures	P10
Main results	16	(*a*) Give unadjusted estimates and, if applicable, confounder-adjusted estimates and their precision (eg, 95% confidence interval). Make clear which confounders were adjusted for and why they were included	P10-P12
		(*b*) Report category boundaries when continuous variables were categorized	-
		(*c*) If relevant, consider translating estimates of relative risk into absolute risk for a meaningful time period	-
Other analyses	17	Report other analyses done—eg analyses of subgroups and interactions, and sensitivity analyses	P12
**Discussion**			
Key results	18	Summarise key results with reference to study objectives	P12-17
Limitations	19	Discuss limitations of the study, taking into account sources of potential bias or imprecision. Discuss both direction and magnitude of any potential bias	P17
Interpretation	20	Give a cautious overall interpretation of results considering objectives, limitations, multiplicity of analyses, results from similar studies, and other relevant evidence	P12-17
Generalisability	21	Discuss the generalisability (external validity) of the study results	P17
**Other information**			
Funding	22	Give the source of funding and the role of the funders for the present study and, if applicable, for the original study on which the present article is based	P18

*Give information separately for cases and controls in case-control studies and, if applicable, for exposed and unexposed groups in cohort and cross-sectional studies.

Note: An Explanation and Elaboration article discusses each checklist item and gives methodological background and published examples of transparent reporting. The STROBE checklist is best used in conjunction with this article (freely available on the Web sites of PLoS Medicine at http://www.plosmedicine.org/, Annals of Internal Medicine at http://www.annals.org/, and Epidemiology at http://www.epidem.com/). Information on the STROBE Initiative is available at www.strobe-statement.org.

For peer review only

BMJ Open

Patient Safety Climate in General Public Hospitals in China: Differences Associated with Department and Job Type Based on a Cross-sectional Survey

Journal:	*BMJ Open*
Manuscript ID	bmjopen-2016-015604.R3
Article Type:	Research
Date Submitted by the Author:	14-Dec-2017
Complete List of Authors:	Zhou, Ping; Fudan University, School of Public Health<br>Bai, Fei; National center for medical service administration<br>Tang, Huiqing; Health and Family Planning Commission of Hubei Province<br>Bai, Jie; Fudan University School of Public Health<br>Li, Minqi; Fudan University School of Public Health<br>Xue, Di; Fudan University School of Public Health
**Primary Subject Heading**:	Health services research
Secondary Subject Heading:	Patient-centred medicine, Medical management
Keywords:	patient safety climate, working department, job type, public hospital, China

SCHOLARONE™
Manuscripts

Patient Safety Climate in General Public Hospitals in China: Differences Associated with Department and Job Type Based on a Cross-sectional Survey

Authors

Ping Zhou[1], Fei Bai[2], Huiqing Tang[3], Jie Bai[1], Minqi Li[1], Di Xue[1]

[1] Key Laboratory of Health Technology Assessment (MOH); School of Public Health, Fu Dan University, Shanghai 200032, P.R. China

[2] National Center for Medical Service Administration, Beijing, 100044, P.R. China

[3] Health and Family Planning Commission of Hubei Province, Wuhan, 430071, P.R. China

Corresponding author:

Di Xue

Address: P.O. Box 197, 138 Yi Xue-yuan Rd., Shanghai, 200032, P.R. China

Email: xuedi@shmu.edu.cn

Fax: 86-21-33565181

Word count for the main text of the manuscript: 3824

For peer review only

Abstract (238 words)

Objective: This study analyzed differences in the perceived patient safety climate among different working departments and job types in public general hospitals in China.

Design: Cross-sectional survey.

Setting: Eighteen tertiary hospitals and 36 secondary hospitals from 10 areas in Shanghai, Hubei Province and Gansu Province, China.

Participants: Overall, 4,753 staff, including physicians, nurses, medical technicians, and managers, were recruited from March to June 2015.

Main outcome measure: The Patient Safety Climate in Health Care Organizations (PSCHO) tool and the percentages of "problematic responses" (PPRs) were used as outcome measures. Multivariable two-level random intercept models were applied in the analysis.

Results: A total of 4,121 valid questionnaires were collected. Perceptions regarding the patient safety climate varied among departments and job types. Physicians responded with relatively more negative evaluations of "organizational resources for safety," "unit recognition and support for safety efforts," "psychological safety," "problem responsiveness" and overall safety climate. Pediatrics departments, intensive care units (ICUs), emergency departments (EDs) and clinical auxiliary departments (CADs) require more attention. The PPRs for "fear of blame and punishment" were universally significantly high, and the PPRs for "fear of shame" and "provision of safe care" were remarkably high, especially in some departments. Departmental differences across all dimensions and the overall safety climate primarily depended on job type.

Conclusions: The differences suggest that strategies and measures for improving the patient safety climate should be tailored by working department and job type.

Keywords

patient safety climate, working department, job type, public hospital, China

For peer review only

Strengths and limitations of this study

- This study was conducted in Shanghai Municipality, Hubei Province and Gansu Province, which represent high, middle, and low socioeconomic status levels in the eastern, central and western regions of China, using a large sample of 4,176 respondents from public general hospitals.
- This study is the first to investigate variations in the perceived patient safety climate among different departments and job types and their interaction in China's public general hospitals.
- We used 2-level random intercept models to examine the relationships between working departments/job types and the patient safety climate survey responses.
- To depict differences by job type within selected working departments, we incorporated the interaction of the working department and job type variables in the model and graphically displayed the predictions using a heat map.
- Although our analyses represent an important advance over prior studies because we adjusted for important known individual and hospital characteristics, other characteristics that were not measured could play a role in distinguishing personnel by working department and job type.
- The results from 54 public general hospitals in three regions might not be generalizable to all hospitals in China, although our sample size was large and represented public hospitals in high, middle, and low socioeconomic level regions in the eastern, central, and western regions of China.

For peer review only

Introduction

Patient safety is a core issue in health care services. Both hospitals and the Chinese government have expended substantial efforts to strengthen patient safety climate and improve patient safety performance.[1-4]

Because patient safety climate is associated with positive outcomes such as greater error reporting,[5] fewer adverse events,[6,7] lower mortality rates,[8] and lower readmission rates,[9] measuring patient safety climate and understanding its variations can be helpful for targeting efforts to improve patient safety.[10-12] However, patient safety climate can vary within organizations. Previous studies have indicated that the patient safety climate of particular departments varies both across and within institutions.[13-17] Therefore, neglecting the patient safety climate at the unit level can mask important local variations, and measuring individual departments' patient safety climate can identify important opportunities for improvement.

The previous literature has suggested that variations in patient safety climate may be related to the pace and complexity of the work performed in different work areas.[15,16] Most studies investigating unit-specific climates have focused on measuring the climate in one or more types of departments with higher levels of intrinsic risk, such as the operating room (OR), the intensive care unit (ICU), and/or the emergency department (ED).[13-16,18] However, research concerning the patient safety climates of other units, such as the pediatric, internal medicine, surgery, and clinical auxiliary departments, is insufficient.

Some studies have measured the perception of the patient safety climate among personnel by job type.[14,19-22] In some studies, physicians demonstrated more positive perceptions of the patient safety climate than did nurses and other clinical personnel.[16,18,21] However, in a previous study conducted in hospitals in Pudong

For peer review only

New Area, Shanghai (a Chinese municipality), we found that physicians had more negative perceptions of the patient safety climate than did nurses.[22]

Given the variations in the perception of the patient safety climate among different departments and job types, efforts to improve patient safety climate should not be limited to interventions at the hospital level but should extend to the department level and different types of employees. Few articles have focused on patient safety climates at the department level and among different types of employees in hospitals in China.

This study analyzed differences in the perception of the patient safety climate among different working departments and job types in 54 public hospitals located in the eastern, central and western regions of China. We selected not only intrinsically hazardous departments but also other departments, including the internal medicine, surgery, obstetrics and gynecology, pediatric, and clinical auxiliary departments. We specifically explored (1) in which departments the staff perceived the patient safety climate more negatively; (2) in which job types the staff gave lower scores across safety climate dimensions; and (3) whether there were differences by job type in the selected working departments.

Methods

Survey instrument

In the study, we applied the Patient Safety Climate in Healthcare Organizations (PSCHO) tool[23-25] to measure patient safety climate. Although various instruments are available to measure a hospital's safety climate,[26] we selected the PSCHO tool because of its good reliability and validity[22,24,25]. Moreover, it includes "fear of blame" and "fear of shame" to measure potential barriers to improving patient safety,[27] both of which capture underlying characteristics of Chinese culture.[22,28,29]

For peer review only

The PSCHO contains 12 dimensions and four categories (based on hospital, work unit, interpersonal contributions to the safety climate and other aspects of the safety climate).[23,24] The PSCHO items use a five-point Likert scale ranging from "strongly disagree" to "strongly agree," with a neutral midpoint.

In this study, we added two items to the PSCHO: "Staff can freely voice their opinions on patient safety" in the dimension "Psychological Safety," and "We analyze accidents or unexpected events in a timely manner" in the dimension "Problem Responsiveness." These items were added because they reflect a more general psychological safety climate (not specific to certain concerns) and timely responses to adverse events in hospitals, respectively. The survey also asked informants to provide demographic information, including gender, age, education, working years, monthly income, working department and job type.

Sample

A cross-sectional survey was conducted using a stratified sampling method in Shanghai Municipality, Hubei Province and Gansu Province in China from March to June of 2015. The regions were selected to capture various socioeconomic statuses and geographic distributions within China. First, we selected 3 provinces/municipalities representing high, middle, and low socioeconomic status levels located in the eastern, central, and western regions of China.

Hubei Province has 12 prefecture-level cities and 1 autonomous prefecture. Gansu Province has 12 prefecture-level cities and 2 autonomous prefectures. We selected 3 prefecture-level cities or autonomous prefectures (areas) in both Hubei Province and Gansu Province to represent high, middle, and low socioeconomic status levels within each province. In each area, two tertiary public general hospitals and four secondary public general hospitals were selected.

For peer review only

Shanghai Municipality has 16 districts. Because Shanghai's tertiary hospitals are not evenly distributed among these districts, 6 tertiary public general hospitals were selected to represent tertiary hospitals owned by universities, the Shanghai government, or district governments. Additionally, 12 secondary public general hospitals were selected from 4 districts (A-D) (areas). Because District A is the largest district in Shanghai and comprises both urban and rural areas covering approximately 20% of Shanghai's total population, 6 secondary public general hospitals were selected in this district. In the other 3 districts in Shanghai, 2 secondary public general hospitals were selected.

The sampled public hospitals, including 18 tertiary hospitals and 36 secondary hospitals, represent public hospitals in China quite well, with different numbers of beds (90~3283 beds), at different levels (secondary vs. tertiary), from the eastern, central and western regions, and from provinces and areas with different social and economic statuses.

Data sources

For each selected hospital, general data (i.e., hospital level, number of beds, number of physicians, and number of nurses) were collected, and anonymous, paper-based, self-administered employee questionnaires were distributed and collected by trained coordinators in the surveyed hospitals or regions according to our study design.

In the employee surveys, we randomly sampled 10% of the managers and administrative staff (at least 15), 10% of the frontline physicians (at least 15), nurses (at least 15) and health technicians and staff working in medical auxiliary departments (at least 5) in each hospital. The term manager refers to a hospital or department director (including working departments and administrative offices), and the term administrative staff refers to non-managerial employees working in administrative offices related to patient safety and medical quality. Frontline workers are non-managerial employees who interact directly with patients. Employees

For peer review only

working in internal medicine, surgery, obstetrics and gynecology, pediatrics, the ICU, the ED, anesthesiology and operating room (OR), clinical auxiliary departments, and other departments (including stomatology, dermatology, ENT, ophthalmology, psychiatry, traditional Chinese medicine departments, administrative and supporting departments) were recruited. The term "clinical auxiliary departments" refers to laboratory departments, imaging departments, electrocardiograph departments, pathology departments, pharmacy departments, supply centers, etc.

This study was approved by the Institutional Review Board of the School of Public Health, Fudan University (IRB# 2014-03-0502), and written informed consent was exempted because the questionnaire survey of employees was anonymous and had less than minimum risk. During the survey, anonymous, self-administered questionnaires were distributed and collected according to our study design by trained coordinators who were employees in the surveyed hospitals or administrators from the local health bureaus. Sampled employees who were willing to participate in our study filled out the questionnaires and returned them to the coordinators.

Survey Data Analysis

Psychometric analysis

We conducted item analyses and confirmatory factor analysis to test the reliability and validity of the PSCHO revised for the Chinese context. In the confirmatory factor analysis, we used PROC CALIS in SAS (SAS Institute Inc., Cary, NC) to form a structural equation model with the raw data. The analysis showed that eleven dimensions had high internal consistency (Cronbach's α coefficients ranged from 0.77 to 0.93), but one dimension ("fear of blame and punishment") had a lower Cronbach's α coefficient (0.66). The overall scale had a high Cronbach's α coefficient (0.96). The standardized root mean square residual (SRMR) and the root mean square error of approximation (RMSEA) of the PSCHO were 0.049 and 0.058, respectively. The Bentler's comparative fit index (CFI), the Bentler-Bonett normed

For peer review only

fit index (NFI) and the non-normed index values were 0.913, 0.906, and 0.907, respectively. The adjusted goodness of fit (AGFI) was 0.83. However, the goodness of fit index (GFI) was 0.84, which is slightly lower than the criterion for this index (GFI >0.85). Overall, the constructive validity of the PSCHO revised for the Chinese cultural context in this study was acceptable.[30-32]

Statistical analysis

We used the percentage of "problematic responses" (PPRs) to measure patient safety climate. A rating of less than 3 for a positive statement or greater than 3 for a negative statement was identified as a problematic response. A lower PPR is indicative of a better perception of the safety climate. This scoring method identifies areas of non-uniformity in safety that are of potential concern and may benefit from interventions to improve the safety climate.[21,22,33-35]

We computed the PPR for each safety climate dimension, with each item in the dimension weighted equally. We also calculated the average PPR for all questions in the survey as a summary statistic, which we referred to as the "overall safety climate." These percentages were calculated as the averages of all responses received. Comparisons among working departments and job types were calculated separately for respondents who indicated that they worked in any of the 9 types of working departments (internal medicine, surgery, obstetrics and gynecology, pediatrics, ICU, ED, anesthesiology and OR, clinical auxiliary departments (CADs) and others) and then for employees by different job type (frontline physicians, frontline nurses, medical technicians, managers and others).

A two-level random intercept model was used to examine how working department and job type affected each dimension and the overall patient safety climate, with the hospital as the level-2 cluster. All models controlled for other respondent characteristics (gender, age, education, number of working years, and monthly

For peer review only

income) and hospital characteristics (region, hospital level, bed size, and doctor-nurse ratio). When estimating the predicted PPR values for each dimension and overall by working department and job type, mean values and proportions were used for continuous covariates and categorical covariates, respectively.

To test the appropriateness of using a two-level model to account for the nesting of individuals within hospitals, we first calculated and tested the intra-class correlation coefficient (ICC) of the empty model, which included no independent variables for each dimension and the overall PPR.[36] The results revealed significant differences in employees' perceptions of the patient safety climate among hospitals ($P<0.0001$).

To depict differences by job type (focusing on physicians with various professional titles and other employees) within selected working departments, we added the interaction of the working department and job type variables to the models. To provide an immediate visual summary of the relevant predictions, we applied a heat map that provided a two-dimensional representation of the data using colors. We focused on physicians in the interaction analysis because they are the most important staff members in healthcare services, and we intended to investigate whether there were differences among chief physicians, attending physicians, residents or below and other personnel within a department.

Statistical analyses and graphics were generated using SAS 9.20, Excel 2007 and R software 3.3.3.

Results

Respondent Characteristics

In this study, 4,176 staff members from 54 selected hospitals responded to the survey. The response rate and the valid response rate were 87.86% and 86.70%, respectively. Among the valid respondents, 55.67% worked in secondary hospitals,

For peer review only

50.81% worked in Shanghai hospitals, and 52.15% worked in hospitals with a large number of beds (>800 beds). The percentages of respondents working in the internal medicine, surgery, obstetrics and gynecology, pediatric, ICU, ED, anesthesiology, OR and CADs were 19.46%, 18.99%, 6.54%, 3.15%, 3.59%, 6.08%, 1.55%, 2.21% and 13.51%, respectively. By job type, 38.66% were frontline physicians (including 10.32% associate chief physicians or chief physicians, 15.53% attending physicians, and 12.81% residents or below), 36.61% were frontline nurses, 9.28% were medical technicians and 2.87% were managers.

The respondents were predominantly female (66.31%) and older than 45 years of age (52.43%). Nearly 52% of the respondents had worked in their hospitals for 10 years or more (Appendix A).

Perceptions of Safety Climate

In this multi-region study, the mean PPR of the overall safety climate among all 54 hospitals was 9.00%. The dimensions with the four highest PPRs were "fear of blame and punishment" (64.81%), "fear of shame" (20.42%), "provisions of safe care" (16.31%), and "organizational resources for safety" (9.55%) (Table 1).

Variations among Clinical Departments

After controlling for the hospital and other individual characteristics, the top four predicted PPRs for the overall safety climate were in the ED (9.63%), CADs (9.52%), pediatrics (9.46%) and the ICU (9.19%); the two lowest predicted PPRs were in the obstetrics and gynecology department (6.82%) and anesthesiology and the OR (7.44%).

The PPRs for “fear of shame” and "fear of blame and punishment" were universally very high across different types of departments. In addition, the PPRs of “fear of shame” and "fear of blame and punishment" in surgery departments were both the highest among the 9 types of departments (23.51% and 69.93%, respectively).

For peer review only

Moreover, the PPR of "organizational resources for safety" in pediatrics departments was the highest across the various types of departments (14.41%) (Table 2).

Variations among Job Types

After adjusting for other personnel and hospital characteristics, the PPR reported by physicians was the highest for the overall safety climate (10.19%), whereas the PPR among managers was the lowest (7.45%). The PPRs for "fear of shame" and "fear of blame and punishment" were generally high among staff members of various job types (Table 3).

Physicians perceived "organizational resources for safety" more negatively (PPR=12.25%) than non-professionals and non-managers (PPR=6.96%). Physicians reported a higher PPR for "unit recognition and support for safety efforts" (9.78%) than nurses (5.45%) and non-professionals and non-managers (4.48%). Physicians' perceptions of "psychological safety" and "problem responsiveness" were also worse than those of all other staff members (Table 3, Appendix B). Additionally, the managers' responses to many dimensions seemed to be similar to those of frontline workers (Appendix C).

Variations within Working Departments among Physicians with Various Titles and Other Personnel

The results revealed that the PPRs reported by various types of staff members in pediatrics, ICU, and CADs differed across many dimensions. For example, chief physicians and associate chief physicians in pediatrics responded more negatively than other physicians and personnel on "organizational resources for safety" (37.03%). However, attending physicians and residents or below reported higher PPRs for "provision of safe care" (29.56% and 26.17%, respectively) than other physicians and personnel (Figure 1).

For peer review only

In the ICU, attending physicians seemed to respond particularly negatively across almost all dimensions, with PPRs above 10%. However, chief physicians and associate chief physicians' PPR for the "provision of safe care" was remarkably higher than that of other physicians and personnel (65.10%) (Figure 1). In the CADs, the PPRs of residents or below were slightly higher across many dimensions, such as "overall emphasis on patient safety," "unit safety norms," "unit recognition and support for safety efforts," "collective learning," "psychological safety" and "problem responsiveness." (Figure 1).

Discussion

Patient Safety Climate in Hospitals

Patient safety climate is a core determinant that ensures and promotes hospital safety in China. The Chinese Hospital Association (CHA) has promulgated annual patient safety goals since 2007, and the National Health and Family Planning Commission (previously known as the Ministry of Health) established a web-based voluntary adverse event reporting system in 2008. Additionally, many provincial governments have actively facilitated the use of clinical pathways, disease management programs, and computer-assisted quality and safety programs; they have also linked government subsidies for public hospitals to the quality and safety of their medical care.[37,38] These efforts all aim to facilitate an improved patient safety climate in China's hospitals.

The survey results revealed that perceptions of the overall patient climate were relatively good (PPR=9%). However, substantial attention should be paid to the dimensions "fear of blame and punishment" (65%), "fear of shame" (20%), "provision of safe care" (16%) and "organizational resources for safety" (10%) based on high-reliability organization (HRO) theories.[39] The high prevalence of "fear of blame and punishment" and "fear of shame" among staff members with different job types and in various working departments may be attributed to

For peer review only

inappropriate systems for performance assessments and rewards, a hierarchical management style, difficult doctor-patient relationships and the quintessentially Chinese notion of “face.” Because a hierarchical management style and the quintessential notion of “face” are common in Chinese culture, high PPRs for “fear of blame and punishment” and “fear of shame” may exist in other hospitals in which the dominant Chinese culture is present. However, we also found that the PPRs of these two dimensions were significantly lower than those in the previous study in Pudong New Area (78.53% and 41.16%, respectively).[22] It is possible that an increasing number of policymakers, hospital managers and health professionals have gradually realized that concealing mistakes or errors might result in worse consequences and have therefore implemented measures such as improving reward systems, changing the patient safety climate, and establishing information systems for easier error reporting to encourage the reporting of errors or mistakes.[40,41]

General Safety Climate Variations by Department

Our study highlighted the very high proportion of problematic responses related to "fear of shame" and "fear of blame and punishment" that universally appeared across the 9 types of departments. This result revealed the most jarring and prevailing issues within Chinese public hospitals, as discussed above. We also found that personnel in pediatrics, the ICU, the ED, and CADs perceived slightly lower levels of overall safety climate than personnel in the other working departments (PPRs were very close to 10%). Staff members in obstetrics and gynecology departments and in anesthesiology departments and ORs responded slightly positively for overall safety climate. For anesthesiology and ORs, we found some previous studies with similar results, which indicates that more resources may have been provided and greater efforts exerted to overcome safety hazards in anesthesiology and ORs.[15,16] Furthermore, the results showed that the respondents in surgery departments reported the highest PPR for "fear of shame." In general, surgeons, who are physicians with high professional titles, have greater social identity and self-identity. However, medical errors and/or accidents occurring in surgery are usually more

For peer review only

severe than those in other working departments. Therefore, staff members in these departments experience more pressure and face more serious consequences.

General Safety Climate Variations by Job Type

The results showed that physicians' PPRs were systematically higher than those of other staff members in this study, whereas Western studies have shown the opposite.[13,16,19,42] Most frontline physicians in China are full-time employees of public hospitals. They hold dominant positions and are close to patients in the process of providing medical services. They are also responsible for ensuring patient safety and quality. If adverse events or healthcare errors occur, patients are more likely to complain and blame their physicians. Accordingly, frontline physicians might pay more attention to quality and safety measures and systems, and they also might obtain more firsthand experience or information about safety hazards.

This finding indicates that hospital managers and unit directors should establish and continuously improve internal managerial systems to encourage frontline physicians to report or share their experiences and information regarding patient safety and healthcare quality and to facilitate their proactive participation in related improvement projects. Measures to appoint frontline physicians to serve as unit quality controllers, to encourage them to lead interdisciplinary groups to implement quality control circle activities, and to involve indicators of patient safety and quality improvement in individual performance assessments have been implemented in some hospitals in China and should be generalized.[43-45]

In addition, the results also revealed that managers' perceptions were relatively consistent with those of frontline workers on many dimensions but differed from those reported in many previous studies.[22,24,34,42] This finding might be partly attributed to managers' efforts and interventions to promote "speaking up" and "communicating down" in China's public hospitals, including activities such as

For peer review only

safety culture-oriented simulation training, Leadership WalkRounds, and efforts to engage frontline workers and managers in open discussions about safety events.[46,47] It is also possible, however, that these results were driven by the extent to which the managers in this survey involved working department directors, in contrast to our 2013 study in Shanghai.[22] Working department directors in China are physicians who also provide healthcare for patients on the front lines.

Pediatrics Departments Should Receive More Attention

In this investigation, pediatrics personnel responded with a high PPR (14.41%) for "organizational resources for safety". In particular, chief and associate chief pediatricians were much worried about organizational resources for safety (PPR=37.03%). This is an alarming finding but is consistent with the increasingly severe shortage of pediatricians in China reported in some articles and news stories.[48-50] Statistical analyses have shown that the number of pediatricians in China increased by only 5,000 from 1995-2010,[51] and the current number of pediatric doctors per 1,000 children is only 0.5, which is one-third of the ratio in the US. By contrast, in China, the number of obstetricians and gynecologists per 1,000 maternal-age women (20 to 40 years of age) is estimated to be approximately 1.2, and the number of internists per 1,000 adults (over 14 years of age) is estimated to be nearly 0.65.[52] This issue will become more problematic following the implementation of China's Two-Child Policy. Pediatricians continue to leave medical practice, especially in primary healthcare institutions, and many new medical graduates are unwilling to join.[53] The results also showed that attending physicians and residents with direct and close patient contact reported high PPRs for "provision of safe care" (29.56% and 26.17%, respectively). The human resources shortage in healthcare is very likely to have a negative impact on health care quality and patient safety.

For peer review only

ICUs and EDs Should Receive More Attention

Working in ICUs and EDs is associated with a higher level of risk, complexity, and difficulty, a faster pace and lower predictability.[15,16] These work areas are intrinsically hazardous, and personnel working in these areas are prone to have a high workload, to work long hours and to face substantial pressure, which can result in burnout.[54-57] Previous studies have indicated that burnout among healthcare providers may lead to reduced patient safety.[58-60] Nahrgang et al.[61] generally argued that burned-out employees' mental and physical energy levels decrease safe work behaviors and thus increase the likelihood of errors and work-related injuries. Flinn et al. conducted a post-call assessment of junior doctors after extended work shifts (average 32.75 h) and noted a significant decline in cognitive functioning and clinical decision-making performance.[62] Sharpe et al.[63] showed deteriorating performance of ICU residents during 26 hours of continuous wakefulness. Because of the intrinsic characteristics of ICUs and EDs, we should attach great importance to them. In addition, the results also revealed that the predicted PPRs in ICUs and EDs for overall safety climate ranked in the top four among the working departments. It is very important and practical to design and optimize systems to protect ICU and ED patients from preventable harm. This approach requires a balanced interdisciplinary effort directed at process characteristics and the simultaneous execution of several other measures [64] Recent examples such as standardization of processes,[65-67] adaptation to humans' cognitive limitations,[68] optimization of working conditions,[69] and increased use of supporting information technologies should be explored.

We also found that attending ICU physicians responded more negatively than other physicians and personnel; almost all of their PPRs were above 10%. These health care workers are important personnel on the front lines in their departments and perhaps experience more safety problems.[43] Hospital managers and policy makers should carefully consider their opinions.

For peer review only - http://bmjopen.bmj.com/site/about/guidelines.xhtml

CADs Need Attention

Our study showed that the perceptions of the overall patient safety climate among staff in CADs was the second worst among the 9 types of departments. In Particular, residents in the CADs were more worried about several dimensions, such as "overall emphasis on patient safety," "unit safety norms," "unit recognition and support for safety efforts," "collective learning," "psychological safety" and "problem responsiveness." Hospital managers might not pay sufficient attention to CADs compared with other working departments, especially with respect to the residents' training and motivation in this type of department.

Limitations

First, the data were based on self-reporting, which might involve recall/report bias. Second, because this study was cross-sectional, we could not rule out the potential for omitted variables. Although our analyses represent an important advance over prior studies because we adjusted for important known individual and hospital characteristics, unmeasured characteristics could play a role in distinguishing personnel by working department and job type. Third, the results from the 54 public general hospitals in three regions might not be generalizable to all hospitals in China, although our sample size was reasonably large and represented public hospitals in high, middle, and low socioeconomic level regions and the eastern, central, and western regions of China.

Conclusions

Our study highlights differences in perceptions of the patient safety climate among and within working departments and job types that have not been previously documented in China. The findings indicate that safety climate improvement efforts should involve greater attention to the climate in the ICU, the ED, pediatrics, and CADs. Therefore, it would be effective and reasonable to implement specific

For peer review only

measures to improve the patient safety climate that target specific departments and personnel.

Acknowledgments

We gratefully acknowledge the significant contributions of the following members of the research project team: Hongbo Zhu, Xuefeng Wei, and Jianjun Gu. The authors also acknowledge all hospitals that provided assistance with data collection in this research project. We truly appreciate Dr. Yongfu Yu of the Department of Clinical Epidemiology at Aarhus University Hospital Olof Palmes, who provided us with important guidance on the statistical analysis.

Contributors

Di Xue conceived and designed the study. Di Xue, Fei Bai, Mingqi Li, Huiqing Tang, Jie Bai, and Ping Zhou performed the investigation. Ping Zhou and Mingqi Li conducted statistical analyses and interpreted the data. Ping Zhou drafted and revised the manuscript. Di Xue revised the manuscript. All authors read and approved the final manuscript.

Competing interests

The authors have declared that no competing interest exists.

Funding

This research project was supported by the National Natural Science Foundation of China, grant number 71473047.

Ethics approval

The study was approved by the Institutional Review Board of the School of Public Health, Fudan University (IRB# 2014-03-0502).

For peer review only

Data sharing statement

No additional data are available.

References

1 Cheng YM, Liu Y, Liu YM. Review on research and application of adverse events reporting system in China. *Chin Hosp Manage* 2012;32:40–2.

2 Hu R. WHO release of the Chinese version of the patient safety curriculum guide: multi-professional edition. *Chin Community Physician* 2012;29:23.

3 Zhu J, Li L, Li Y, et al. What constitutes patient safety culture in Chinese hospitals? *Int J Qual Health Care* 2012;24:250-7. doi: 10.1093/intqhc/mzs010.

4 Xu SX, National Health and Family Planning Commission of PR. [Seven measures to improve medical quality and patient safety]. China Hospital CEO 25 Dec 2015. http://www.h-ceo.com/article/read/6978/6978.html (accessed 7 Aug 2016).

5 Braithwaite J, Westbrook MT, Travaglia JF, et al. Cultural and associated enablers of, and barriers to, adverse incident reporting. *Qual Saf Health Care* 2010;19:229–33. doi: 10.1136/qshc.2008.030213.

6 Singer S, Lin S, Falwell A, et al. Relationship of safety climate and safety performance in hospitals. *Health Serv Res* 2009;44:399–421. doi: 10.1111/j.1475-6773.2008.00918.x.

For peer review only

7 Mardon RE, Khanna K, Sorra J, et al. Exploring relationships between Hospital patient safety culture and adverse events. *J Patient Saf* 2010;6:226–32. doi: 10.1097/PTS.0b013e3181fd1a00.

8 Huang DT, Clermont G, Kong L, et al. Intensive care unit safety culture and outcomes: a US multicenter study. *Int J Qual Health Care* 2010;22:151-61.

9 Hansen LO, Williams MV, Singer SJ. Perceptions of hospital safety climate and incidence of readmission. *Health Serv Res* 2011;46:596–616. doi: 10.1111/j.1475-6773.2010.01204.x.

10 Rose JS, Thomas CS, Tersigni A, et al. A leadership framework for culture change in health care. *Jt Comm J Qual Patient Saf* 2006;32:433– 42.

11 Flin R, Burns C, Mearns K, et al. Measuring safety climate in health care. *Qual Saf Health Care* 2006;15:109–15. doi: 10.1136/qshc.2005.014761.

12 Singer SJ, Vogus TJ. Safety climate research: taking stock and looking forward. *BMJ Qual Saf* 2013;22:1–4. doi: 10.1136/bmjqs-2012-001572.

13 Makary MA, Sexton JB, Freischlag JA, et al. Operating room teamwork among physicians and nurses: teamwork in the eye of the beholder. *J Am Coll Surg* 2006;202:746 –52. doi: 10.1016/j.jamcollsurg.2006.01.017.

14 Cooper JB, Blum RH, Carroll JS, et al. Differences in safety climate among hospital anesthesia departments and the effect of a realistic simulation-based training program. *Anesth Analg* 2008;106:574–84. doi: 10.1213/01.ane.0000296462.39953.d3.

For peer review only

For peer review only - http://bmjopen.bmj.com/site/about/guidelines.xhtml

15 Huang DT, Clermont G, Sexton JB, et al. Perceptions of safety culture vary across the intensive care units of a single institution. *Crit Care Med* 2007;35:165–76. doi: 10.1097/01.CCM.0000251505.76026.CF.

16 Singer SJ, Gaba DM, Falwell A, et al. Patient safety climate in 92 US hospitals: differences by work area and discipline. *Med Care* 2009;47:23-31. doi: 10.1097/MLR.0b013e31817e189d.

17 Paine LA, Rosenstein BJ, Sexton JB, et al. Assessing and improving safety culture throughout an academic medical centre: a prospective cohort study. *Qual Saf Health Care* 2010;19:547-54. doi: 10.1136/qshc.2009.039347.

18 Gehring K, Mascherek AC, Bezzola P, et al. Safety climate in Swiss hospital units: Swiss version of the Safety Climate Survey. *J Eval Clin Practice* 2015;21:332–8. doi: 10.1111/jep.12326.

19 Sullivan A, Camargo C, Cleary P, et al. Do emergency physicians and nurses differently perceive safety-related factors? The National ED Safety Study. *Acad Emerg Med* 2007;14:S210–1. doi: 10.1197/j.aem.2007.03.1310.

20 Sexton JB, Holzmueller CG, Pronovost PJ, et al. Variation in caregiver perceptions of teamwork climate in labor and delivery units. *J Perinatol* 2006;26:463– 70. doi: 10.1038/sj.jp.7211556.

21 Thomas EJ, Sexton JB, Helmreich RL. Discrepant attitudes about teamwork among critical care nurses and physicians. *Crit Care Med* 2003;31:956–9. doi: 10.1097/01.CCM.0000056183.89175.76.

For peer review only

22 Zhou P, Bundorf MK, Gu J, et al. Survey on patient safety climate in public hospitals in China. *BMC Health Serv Res* 2015;15:53. doi: 10.1186/s12913-015-0710-x.

23 Singer S, Meterko M, Baker L, et al. Workforce perceptions of hospital safety culture: development and validation of the patient safety climate in healthcare organizations survey. *Health Serv Res* 2007;42:1999–2021. doi: 10.1111/j.1475-6773.2007.00706.x.

24 Hartmann CW, Rosen AK, Meterko M, et al. An overview of patient safety climate in the VA. *Health Serv Res* 2008;43:1263–84. doi: 10.1111/j.1475-6773.2008.00839.x.

25 Singer SJ, Hartmann CW, Hanchate A, et al. Comparing safety climate between two populations of hospitals in the United States. *Health Serv Res* 2009;44:1563–83. doi: 10.1111/j.1475-6773.2009.00994.x.

26 Colla JB, Bracken AC, Kinney LM, et al. Measuring patient safety climate: a review of surveys. *Qual Saf Health Care* 2005;14:364-6.

27 Weick KE, Sutcliffe KM, 2001. Managing the Unexpected. San Francisco, CA: Jossey-Bass.

28 Nie Y, Mao X, Cui H, et al. Hospital survey on patient safety culture in China. *BMC Health Serv Res* 2013;13:228.

29 Chen IC, Li HH. Measuring patient safety culture in Taiwan using the Hospital Survey on Patient Safety Culture (HSOPSC). *BMC Health Serv Res* 2010;10:152.

For peer review only

30 Bentler PM, Bonett DG. Significance tests and goodness of fit in the analysis of covariance structures. *Psychol Bull* 1980;88:588–606.

31 McDonald RP, Ho MHR. Principles and practice in reporting structural Equation Analyses. *Psychol Methods* 2002;7:64–82.

32 Gefen D, Straub DW, Boudreau MC. Structural equation modeling and regression: guidelines for research practice. *Commun Assoc Inform Syst* 2000;4:1-77.

33 Gaba DM, Howard SK, Jump B. Production pressure in the work environment. California anesthesiologists' attitudes and experiences. *Anesthesiology* 1994;81:488–500. doi: 10.1097/00000542-199408000-00028.

34 Singer SJ, Gaba DM, Geppert JJ, et al. The culture of safety: results of an organization-wide survey in 15 California hospitals. *Qual Saf Health Care* 2003;12:112–8. doi: 10.1097/MLR.0b013e31817925c1.

35 Singer SJ, Falwell A, Gaba DM, et al. Patient safety climate in US hospitals: variation by management Level. *Med Care* 2008;46:1149–56.

36 Wang JC, Xie HY, Jiang BF, 2010. Multilevel Models: Methods and Applications. Beijing: Higher Education Press.

37 Gong TX, 2012. Impact of Financial Compensation Policy: an Intervention Study in 15 Hospitals of Traditional Chinese Medicine. Beijing: Beijing University of Chinese Medicine.

For peer review only

38 Zhou X, Wu EZ. The practice and discussion on performance appraisal reform in county-level public hospitals in Haining City. *J Health Econ Res* 2015;8:24-6, 27.

39 Gaba DM, Singer SJ, Sinaiko AD, et al. Differences in safety climate between Hospital personnel and Naval aviators. *Hum Factors* 2003;45:173–85. doi: 10.1518/hfes.45.2.175.27238.

40 Chiang HY, Hsiao YC, Lin SY, et al. Incident reporting culture: scale development with validation and reliability and assessment of hospital nurses in Taiwan. *Int J Qual Health Care* 2011;23:429-36.

41 Sanghera IS, Franklin BD, Dhillon S. The attitudes and beliefs of healthcare professionals on the causes and reporting of medication errors in a UK intensive care unit. *Anaesthesia* 2007;62:53-61.

42 Pronovost PJ, Weast B, Holzmueller CG, et al. Evaluation of the culture of safety: survey of clinicians and managers in an academic medical center. *Qual Saf Health Care* 2003;12:405– 10.

43 He XY, Gu JJ, Sun X, et al. Medical quality control system in public general hospitals in Pudong New Area. *Chin Health Qual Manag* 2015;22:25-8.

44 Wang YQ, Qin XY, Gao X, et al. Quality control circle in the continuous quality improvement campaign of the hospital. *Chin J Hosp Admin* 2014;30:425-7.

45 Liu SJ, Xu MY. The practice of the assessment of medical quality in clinical departments in a general hospital. *Chin Hosp Manag* 2013;33:56-7.

For peer review only

46 Wang LH, Zhao GG, Yang X, et al. On the practice and insights of building a safety-oriented culture in the hospital. *Chin J Hosp Admin* 2009;25:595-8.

47 Liu HS. Reinforcing internal communication to promote the harmonious development of the hospital. *China Science and Technology Review* 2009;26:102.

48 Ren YQ, Lu MH, Zhang Y, et al. Issues and solutions of building a pediatrician pool in Shanghai. *Hospital Administration Journal of Chinese PLA* 2016;23:228-30.

49 Liu SH, Pi GH, Kang Q, et al. Current situation of pediatric in Nanchong of Sichuan Province. *Journal North Sichuan Medical College* 2015;30:109-11.

50 *Xin Hua News.* China faces shortage of pediatricians. 2014. http://news.xinhuanet.com/english/health/2014-09/11/c_133636236.htm (accessed 15 Sept 2016).

51 Zhao CY. China faces critical pediatrician shortage. *China Daily,* 2011. http://www.chinadaily.com.cn/china/2011-02/17/content_12034787.htm (accessed 10 Sept 2016).

52 China Network Television (CNTV). China facing pediatrician shortage. 2016. http://www.china.org.cn/video/2016-01/20/content_37620967.htm (accessed 10 Sept 2016).

53 Pan SJ, Chen JM, Bai JG. Analysis and countermeasures on the causes of pediatric human resources shortage--Take Shanxi Province as an example. *Soft Sci Health* 2016;30:31-4.

For peer review only

54 Lin PH, Xiao HX, Lin XQ. Survey on stress management of emergency physicians and its countermeasures in Fujian Province. *Chin Rural Health Serv Admin* 2014;34:503-6.

55 Wang L, Liu ZF, Peng XW, et al. Present situation investigation and analysis of emergency departments of 20 tertiary general hospitals in Heilongjiang Province. *Management* 2015;35:68-9.

56 Sheng WJ, Zhang S, Zhang L. Analysis on ICU healthcare providers' stress resources and its countermeasures. *Chronic Pathematology Journal* 2010;12:1674-5.

57 Li C, Tian XH, Feng LN, et al. Study on correlation between job stress and coping styles and job burnout of ICU nurses (in Chinese). *Chin Nurs Res* 2014;28:3236-7.

58 Teng CI, Shyu YI, Chiou WK, et al. Interactive effects of nurse-experienced time pressure and burnout on patient safety: a cross-sectional survey. *Int J Nurs Stud* 2010;47:1442–50. doi: 10.1016/j.ijnurstu.2010.04.005.

59 West CP, Tan AD, Habermann TM, et al. Association of resident fatigue and distress with perceived medical errors. *JAMA* 2009;302:1294–300. doi: 10.1001/jama.2009.1389.

60 Halbesleben JR, Rathert C. Linking physician burnout and patient outcomes: exploring the dyadic relationship between physicians and patients. *Health Care Manage Rev* 2008;33:29–39. doi: 10.1097/01.HMR.0000304493.87898.72.

For peer review only

61 Nahrgang JD, Morgeson FP, Hofmann DA. Safety at work: a meta-analytic investigation of the link between job demands, job resources, burnout, engagement, and safety outcomes. *J Appl Psychol* 2011;96:71–94. doi: 10.1037/a0021484.

62 Flinn F, Armstrong C. Junior doctors' extended work hours and the effects on their performance: the Irish case. *Int J Qual HealthCare* 2011;23:210–7. doi: 10.1093/intqhc/mzq088.

63 Sharpe R, Koval V, Ronco JJ, et al. The impact of prolonged continuous wakefulness on resident clinical performance in the intensive care unit: a patient simulator study. *Crit Care Med* 2010;38:766–70. doi: 10.1097/CCM.0b013e3181cd122a.

64 Valentin A. Approaches to decreasing medication and other care errors in the ICU. *Curr Opin Crit Care* 2013;19:474-9. doi: 10.1097/MCC.0b013e328364d4f9.

65 Dehmel C, Braune SA, Kreymann G, et al. Do centrally pre-prepared solutions achieve more reliable drug concentrations than solutions prepared on the ward? *Intensive Care Med* 2011;37:1311–6.

66 Poon EG, Keohane CA, Yoon CS, et al. Effect of bar-code technology on the safety of medication administration. *N Engl J Med* 2010;362:1698–707.

67 Askari M, Eslami S, Louws M, et al. Frequency and nature of drug-drug interactions in the intensive care unit. *Pharmacoepidemiol Drug Saf* 2013;22:430–7.

For peer review only

68 Garrouste-Orgeas M, Soufir L, Tabah A, et al. A multifaceted program for improving quality of care in intensive care units: IATROREF study. *Crit Care Med* 2012;40:468–76.

69 Wallace DJ, Angus DC, Barnato AE, et al. Nighttime intensivist staffing and mortality among critically ill patients. *N Engl J Med* 2012;366:2093–101.

For peer review only

Tables and captions

Table 1 Problematic Responses for Dimensions and Items

Table 2 Patient Safety Climate by Working Department

Table 3 Patient Safety Climate by Job Type

Table 1 Problematic Responses for Dimensions and Items

Dimensions and Items †	% Problematic
Hospital contributions to the safety climate	
Senior managers' engagement	2.84
Organizational resources for safety	9.55
Overall emphasis on patient safety	2.63
Work-unit contributions to the safety climate	
Unit managers' support	5.42
Unit safety norms	4.51
Unit recognition and support for safety efforts	6.68
Collective learning	2.10
Psychological safety	5.68
Problem responsiveness	2.61
Interpersonal contributions to the safety climate	
Fear of shame	20.42
Fear of blame and punishment	64.81
Other aspects of the safety climate	
Provision of safe care	16.31
Overall average	9.00

† The means of all items in a dimension were averaged to calculate the dimension mean.

For peer review only

Table 2 Patient Safety Climate by Working Department †

Dimensions	Internal Medicine		Surgery		Obstetrics & Gynecology	
	Estimate	95% CL	Estimate	95% CL	Estimate	95% CL
Hospital contributions to the safety climate						
Senior managers' engagement	2.74	[1.58,3.90]	2.06	[0.87,3.25]	1.80	[0.21,3.39]
Organizational resources for safety	9.38	[6.90,11.86]	7.54	[5.00,10.09]	7.02	[3.65,10.40]
Overall emphasis on patient safety	2.96	[1.57,4.35]	2.19	[0.76,3.62]	1.56	[-0.38,3.49]
Work-unit contributions to the safety climate						
Unit managers' support	5.21	[3.40,7.01]	4.90	[3.05,6.76]	3.15	[0.65,5.64]
Unit safety norms	3.85	[2.67,5.03]	3.26	[2.05,4.47]	3.92	[2.29,5.55]
Unit recognition and support for safety efforts	5.95	[3.95,7.94]	5.51	[3.46,7.56]	4.29	[1.59,6.99]
Collective learning	2.14	[1.10,3.19]	1.40	[0.33,2.48]	0.91	[-0.53,2.35]
Psychological safety	2.87	[0.94,4.79]	1.83	[-0.15,3.80]	0.72	[-1.92,3.37]
Problem responsiveness	0.93	[-0.46,2.31]	0.42	[-1.00,1.84]	-0.40	[-2.30,1.49]
Interpersonal contributions to the safety climate						
Fear of shame	19.77	[15.84,23.69]	23.51	[19.49,27.53]	12.46	[7.26,17.67]
Fear of blame and punishment	66.45	[61.69,71.21]	69.93	[65.06,74.79]	58.80	[52.60,65.01]
Other aspects of the safety climate						
Provision of safe care	17.19	[13.45,20.93]	17.67	[13.84,21.50]	22.72	[17.75,27.70]
Overall	8.51	[7.57,9.46]	8.28	[7.30,9.25]	6.82	[5.52,8.11]

Continued:

Dimensions	Pediatrics		ICU		ED	
	Estimate	95% CL	Estimate	95% CL	Estimate	95% CL
Hospital contributions to the safety climate						
Senior managers' engagement	2.19	[0.02,4.35]	4.86	[2.84,6.87]	4.62	[2.97,6.28]
Organizational resources for safety	14.41	[9.85,18.97]	10.54	[6.28,14.79]	8.83	[5.32,12.35]
Overall emphasis on patient safety	2.60	[-0.04,5.24]	2.79	[0.33,5.26]	2.71	[0.70,4.73]
Work-unit contributions to the safety climate						
Unit managers' support	4.42	[1.02,7.82]	5.44	[2.27,8.62]	6.29	[3.69,8.89]
Unit safety norms	4.71	[2.50,6.92]	3.72	[1.66,5.79]	5.43	[3.73,7.12]
Unit recognition and support for safety efforts	8.57	[4.93,12.20]	5.10	[1.71,8.48]	8.33	[5.52,11.13]
Collective learning	3.17	[1.22,5.13]	2.54	[0.71,4.36]	2.60	[1.11,4.10]
Psychological safety	9.50	[5.90,13.09]	6.69	[3.34,10.04]	5.29	[2.54,8.05]
Problem responsiveness	0.75	[-1.81,3.32]	1.11	[-1.29,3.50]	2.02	[0.05,4.00]
Interpersonal contributions to the safety climate						
Fear of shame	16.95	[10.01,23.88]	20.25	[13.78,26.71]	20.09	[14.68,25.50]
Fear of blame and punishment	61.47	[53.27,69.67]	60.06	[52.40,67.71]	65.68	[59.24,72.13]
Other aspects of the safety climate						
Provision of safe care	19.48	[12.84,26.12]	22.22	[16.03,28.41]	19.94	[14.77,25.10]
Overall	9.46	[7.70,11.21]	9.19	[7.56,10.83]	9.63	[8.29,10.98]

Continued:

Dimensions	Anesthesiology & OR		CADs		Others	
	Estimate	95% CL	Estimate	95% CL	Estimate	95% CL
Hospital contributions to the safety climate						
Senior managers’ engagement	1.87	[-0.11,3.84]	1.53	[0.27,2.80]	1.89	[0.85,2.94]
Organizational resources for safety	6.10	[1.93,10.28]	7.12	[4.43,9.81]	7.80	[5.55,10.05]
Overall emphasis on patient safety	3.26	[0.85,5.68]	2.44	[0.93,3.96]	1.87	[0.63,3.12]
Work-unit contributions to the safety climate						
Unit managers' support	2.85	[0.26,5.96]	5.80	[3.84,7.77]	4.29	[2.67,5.91]
Unit safety norms	2.36	[0.34,4.38]	6.12	[4.83,7.40]	3.99	[2.93,5.06]
Unit recognition and support for safety efforts	3.03	[-0.30,6.36]	8.97	[6.80,11.13]	5.34	[3.52,7.15]
Collective learning	0.44	[-1.35,2.23]	3.20	[2.06,4.34]	0.91	[-0.03,1.85]
Psychological safety	2.28	[-1.01,5.56]	8.95	[6.86,11.05]	3.05	[1.32,4.78]
Problem responsiveness	0.43	[-1.92,2.78]	5.50	[4.00,7.01]	0.26	[-0.99,1.51]
Interpersonal contributions to the safety climate						
Fear of shame	21.80	[15.44,28.15]	16.22	[11.99,20.45]	15.60	[11.99,19.20]
Fear of blame and punishment	66.92	[59.39,74.45]	68.73	[63.63,73.83]	67.12	[62.72,71.53]
Other aspects of the safety climate						
Provision of safe care	19.52	[13.44,25.60]	16.54	[12.51,20.58]	18.94	[15.51,22.37]
Overall	7.44	[5.84,9.04]	9.52	[8.49,10.55]	7.79	[6.93,8.65]

† The results reflect predicted means (estimate and 95% 95% CL) by clinical departments based on 2-level random intercept models for each dimension and the overall safety climate adjusted for other individual characteristics (age, gender, education, working years and monthly income) and hospital characteristics (tertiary level vs secondary level, hospital size, hospital location, and doctor-nurse ratio). When estimating the predicted mean values of the PPR for each dimension and overall by clinical department and job type, we held other covariates constant at their means.

Table 3 Patient Safety Climate by Job Type †

Dimensions	Physicians		Nurses		Medical technicians	
	Estimate	95% CL	Estimate	95% CL	Estimate	95% CL
Hospital contributions to the safety climate						
Senior managers' engagement	4.01	[3.20,4.81]	2.17	[1.28,3.07]	2.76	[0.95,4.58]
Organizational resources for safety	12.25	[10.48,14.02]	9.97	[8.02,11.92]	8.86	[5.04,12.69]
Overall emphasis on patient safety	3.36	[2.43,4.29]	2.37	[1.34,3.41]	1.83	[-0.38,4.04]
Work-unit contributions to the safety climate						
Unit managers' support	6.15	[4.92,7.38]	5.50	[4.13,6.86]	4.26	[1.41,7.10]
Unit safety norms	4.92	[4.11,5.74]	4.99	[4.09,5.90]	2.93	[1.07,4.78]
Unit recognition and support for safety efforts	9.78	[8.33,11.22]	5.45	[3.87,7.04]	6.19	[3.14,9.24]
Collective learning	2.86	[2.14,3.58]	1.80	[0.99,2.60]	0.75	[-0.89,2.39]
Psychological safety	8.30	[6.96,9.63]	5.84	[4.35,7.32]	3.90	[0.89,6.91]
Problem responsiveness	4.09	[3.12,5.07]	1.63	[0.56,2.71]	0.10	[-2.05,2.25]
Interpersonal contributions to the safety climate						
Fear of shame	18.39	[15.43,21.35]	19.76	[16.56,22.97]	17.48	[11.64,23.33]
Fear of blame and punishment	65.04	[61.35,68.73]	63.07	[59.10,67.03]	62.67	[55.72,69.61]
Other aspects of the safety climate						
Provision of safe care	17.57	[14.76,20.37]	21.07	[18.03,24.11]	18.98	[13.39,24.58]
Overall	10.19	[9.52,10.86]	8.83	[8.09,9.57]	7.89	[6.42,9.36]

Continued:

Dimensions	Managers ‡		Non-professionals and non-managers	
	Estimate	95% CL	Estimate	95% CL
Hospital contributions to the safety climate				
Senior managers' engagement	2.39	[-0.06,4.83]	1.76	[0.49,3.03]
Organizational resources for safety	5.71	[0.56,10.85]	6.96	[4.25,9.67]
Overall emphasis on patient safety	3.12	[0.11,6.13]	1.76	[0.24,3.28]
Work-unit contributions to the safety climate				
Unit managers' support	4.38	[0.52,8.24]	3.25	[1.28,5.22]
Unit safety norms	3.36	[0.85,5.86]	4.56	[3.27,5.85]
Unit recognition and support for safety efforts	4.70	[0.60,8.79]	4.48	[2.30,6.65]
Collective learning	1.72	[-0.49,3.94]	2.49	[1.35,3.64]
Psychological safety	-0.41	[-4.48,3.65]	5.26	[3.15,7.36]
Problem responsiveness	-1.32	[-4.22,1.58]	1.61	[0.10,3.12]
Interpersonal contributions to the safety climate				
Fear of shame	18.95	[11.19,26.72]	17.99	[13.73,22.24]
Fear of blame and punishment	66.40	[57.25,75.54]	67.92	[62.78,73.06]
Other aspects of the safety climate				
Provision of safe care	20.67	[13.24,28.11]	18.50	[14.45,22.56]
Overall	7.45	[5.47,9.43]	8.21	[7.18,9.25]

† The results reflect predicted means (estimate and 95% 95% CL) for clinical departments based on 2-level random intercept models for each dimension and the overall safety climate adjusted for other individual characteristics (age, gender, education, working years, and monthly income) and hospital characteristics (tertiary level vs secondary level, hospital size, hospital location, and doctor-nurse ratio). When estimating the predicted mean values of the PPR for each dimension and overall by clinical department and job type, we held other covariates constant at their means.

‡ Including managers in clinical departments, CADs, and administrative departments.

For peer review only - http://bmjopen.bmj.com/site/about/guidelines.xhtml

Figures and legends

Figure 1. Heatmap of predictive means of staff among 9 working departments across physicians with various titles and other staff on each dimension and the overall safety climate.

For peer review only

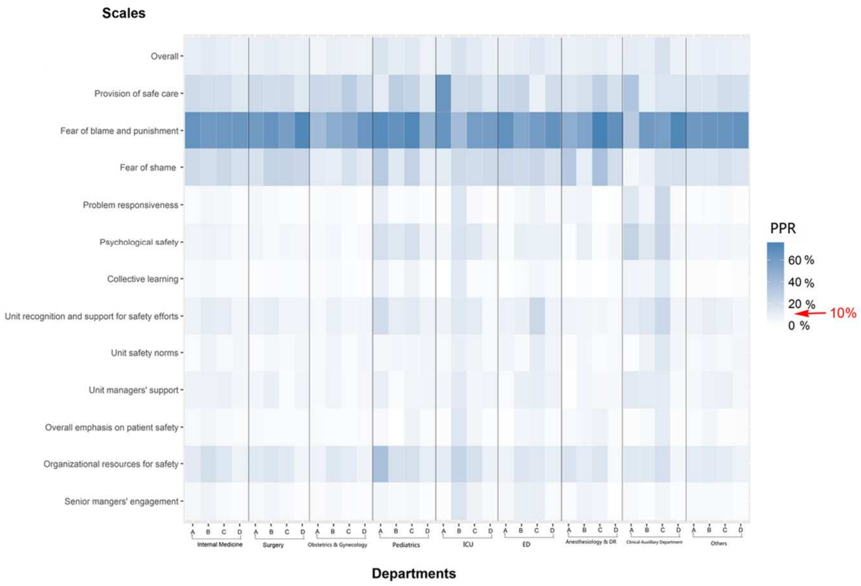

Figure 1. Heatmap of predictive means of staff among 9 working departments across physicians with various titles and other staff on each dimension and the overall safety climate

Note: A-chief physicians or associate chief physicians; B-attending physicians; C-residents or below; D-others

Figure 1. Heatmap of predictive means of staff among 9 working departments across physicians with various titles and other staff on each dimension and the overall safety climate.

115x84mm (300 x 300 DPI)

For peer review only - http://bmjopen.bmj.com/site/about/guidelines.xhtml

Appendix A: General Respondent Information

Work Area Characteristics	N	%	Worker Characteristics	N	%
Region			Gender		
Shanghai	2094	50.81	Male	1365	33.69
Hubei Province	1095	26.57	Female	2687	66.31
Gansu Province	932	22.62	Total	4052	100.00
Total	4121	100.00	Age		
Hospital level			<25	269	6.67
Tertiary	1827	44.33	25-34	771	19.11
Secondary	2294	55.67	35-45	879	21.79
Total	4121	100.00	>=45	2115	52.43
Hospital size (beds)			Total	4034	100.00
Small (≤500)	841	20.41	Education		
Middle (501-800)	1131	27.44	Master's degree or above	737	18.03
Large (>800)	2149	52.15	Bachelor's degree	2205	53.94
Total	4121	100.00	Associate's degree or below	1146	28.03
Affiliated with medical school			Total	4088	100.00
Yes	2644	65.08	Working years		
No	1419	34.92	<5	1073	26.63
Total	4063	100.00	5-10	852	21.15
Clinical department			>10	2104	52.22
Internal Medicine	791	19.46	Total	4029	100.00
Surgery	772	18.99	Monthly income (RMB)		
Obstetrics & Gynecology	266	6.54	<4000	1311	32.25
Pediatrics	128	3.15	4000-7999.99	1767	43.47
ICU	146	3.59	≥8000	987	24.28
Emergency Department (ED)	247	6.08	Total	4065	100.00
Anesthesiology	63	1.55	Job type		
Operating Room (OR)	90	2.21	Frontline physicians	1563	38.66
Clinical Auxiliary Departments (CADs)†	549	13.51	*(Associate) Chief physicians*	*417*	*10.32*
Others△	1013	24.92	*Attending physicians*	*628*	*15.53*
Total	4065	100.00	*Resident or below*	*518*	*12.81*
			Frontline nurses	1480	36.61
Doctor-nurse ratio			Medical technicians	375	9.28
Mean (SD)	0.81	0.59	Managers‡	116	2.87
Bed-Nurse ratio			Non-professionals and non-managers	509	12.58
Mean (SD)	2.53	2.52	Total	4043	100.00

† Refers to laboratory department, image department, ECG department, pathology department, pharmacy department, supply center, etc.

△ Including stomatology, dermatology, ENT, ophthalmology, psychiatry, traditional Chinese medicine departments, and administrative offices.

‡ Including managers in the clinical departments, clinical auxiliary departments, and administrative departments.

For peer review only

Appendix B: Differences in Patient Safety Climate among Job Types (Pair-wise Comparison with Physicians) †

Dimensions	Nurses-Physicians		Medical Technicians-Physicians		Managers-Physicians		Others-Physicians	
	Estimate	95% CL	Estimate	95% CL	Estimate	95% CL	Estimate	95% CL
Hospital contributions to the safety climate								
Senior managers' engagement	-1.83	[-3.29,-0.38]	-1.24	[-3.71,1.23]	-1.62	[-4.82,1.58]	-2.25	[-3.96,-0.53]
Organizational resources for safety	-2.28	[-5.34,0.77]	-3.39	[-8.56,1.78]	-6.55	[-13.24,-0.15]	-5.30	[-8.89,-1.70]
Overall emphasis on patient safety	-0.98	[-2.78,0.81]	-1.53	[-4.59,1.53]	-0.24	[-4.20,3.72]	-1.60	[-3.71,0.51]
Work-unit contributions to the safety climate								
Unit managers' support	-0.65	[-2.95,1.65]	-1.89	[-5.80,2.02]	-1.76	[-6.83,3.30]	-2.90	[-5.6,-0.19]
Unit safety norms	0.07	[-1.43,1.56]	-2.00	[-4.53,0.53]	-1.57	[-4.85,1.71]	-0.36	[-2.12,1.39]
Unit recognition and support for safety efforts	-4.32	[-6.75,-1.90]	-3.59	[-7.68,0.51]	-5.08	[-10.39,-0.23]	-5.30	[-8.16,-2.44]
Collective learning	-1.06	[-2.38,0.26]	-2.11	[-4.35,0.13]	-1.13	[-4.04,1.77]	-0.36	[-1.92,1.19]
Psychological safety	-2.46	[-4.89,-0.04]	-4.40	[-8.51,-0.29]	-8.71	[-14.03,-3.39]	-3.04	[-5.89,-0.19]
Problem responsiveness	-2.46	[-4.19,-0.73]	-3.99	[-6.92,-1.07]	-5.41	[-9.20,-1.62]	-2.48	[-4.51,-0.45]
Interpersonal contributions to the safety climate								
Fear of shame	1.37	[-3.18,5.93]	-0.90	[-8.59,6.78]	0.57	[-9.39,10.52]	-0.40	[-5.78,4.97]
Fear of blame and punishment	-1.98	[-7.30,3.34]	-2.38	[-11.34,6.59]	1.35	[-10.26,12.96]	2.88	[-3.41,9.16]
Other aspects of the safety climate								
Provision of safe care	3.50	[-0.87,7.87]	1.42	[-5.96,8.80]	3.11	[-6.45,12.66]	0.94	[-4.22,6.09]
Overall	-1.36	[-2.53,-0.18]	-2.29	[-4.28,-0.3]	-2.74	[-5.32,-0.16]	-1.97	[-3.36,-0.59]

† Results reflect differences in predicted values (estimate and 95% 95% CL) among job types by pair-wise comparison with physicians based on 2-level random intercept models for each dimension and the overall safety climate adjusted for other individual characteristics (age, gender, education, working years, and monthly income) and hospital characteristics (tertiary level vs secondary level, hospital size, hospital location, and doctor-nurse ratio). When estimating the predicted mean values of the PPR for each dimension and overall by clinical department and job type, we held other covariates constant at their means.

For peer review only

Appendix C: Differences in Patient Safety Climate among Job Types (Pair-wise Comparison with Managers) †

Dimensions	Physicians-Managers		Nurses-Managers		Medical Technicians -Managers		Others-Managers	
	Estimate	95% CL	Estimate	95% CL	Estimate	95% CL	Estimate	95% CL
Hospital contributions to the safety climate								
Senior managers' engagement	1.62	[-1.38,4.62]	-0.21	[-3.36,2.94]	0.38	[-2.47,3.23]	-0.63	[-3.74,2.49]
Organizational resources for safety	6.55	[0.28,12.82]	4.27	[-2.33,10.86]	3.16	[-2.81,9.12]	1.25	[-5.27,7.78]
Overall emphasis on patient safety	0.24	[-3.47,3.94]	-0.75	[-4.64,3.15]	-1.29	[-4.82,2.23]	-1.36	[-5.21,2.00]
Work-unit contributions to the safety climate								
Unit managers' support	1.76	[-2.98,6.51]	1.11	[-3.87,6.10]	-0.13	[-4.64,4.38]	-1.13	[-6.07,3.80]
Unit safety norms	1.57	[-1.50,4.64]	1.64	[-1.60,4.87]	-0.43	[-3.35,2.49]	1.21	[-1.99,4.40]
Unit recognition and support for safety efforts	5.08	[0.11,10.05]	0.76	[-4.47,5.99]	1.50	[-3.23,6.23]	-0.22	[-5.39,4.96]
Collective learning	1.13	[-1.58,3.85]	0.07	[-2.78,2.93]	-0.97	[-3.56,1.61]	0.77	[-2.05,3.60]
Psychological safety	8.71	[3.73,13.70]	6.25	[1.01,11.49]	4.31	[-0.43,9.06]	5.67	[0.48,10.85]
Problem responsiveness	5.41	[1.86,8.96]	2.95	[-0.78,6.68]	1.42	[-1.96,4.79]	2.93	[-0.76,6.62]
Interpersonal contributions to the safety climate								
Fear of shame	-0.57	[-9.89,8.76]	0.81	[-9.00,10.62]	-1.47	[-10.34,7.41]	-0.97	[-10.68,8.74]
Fear of blame and punishment	-1.35	[-12.23,9.52]	-3.33	[-14.78,8.11]	-3.73	[-14.08,6.63]	1.52	[-9.81,12.85]
Other aspects of the safety climate								
Provision of safe care	-3.11	[-12.05,5.84]	0.39	[-9.02,9.81]	-1.69	[-10.21,6.83]	-2.17	[-11.49,7.15]
Overall	2.74	[0.32,5.15]	1.38	[-1.16,3.92]	0.44	[-1.85,2.74]	0.76	[-1.75,3.28]

† Results reflect differences in predicted values (estimate and 95% 95% CL) among job types by pair-wise comparison with managers based on 2-level random intercept models for each dimension and the overall safety climate adjusted for other individual characteristics (age, gender, education, working years, and monthly income) and hospital characteristics (tertiary level vs secondary level, hospital size, hospital location, and doctor-nurse ratio). When estimating the predicted mean values of the PPR for each dimension and overall by clinical department and job type, we held other covariates constant at their means.

For peer review only

STROBE Statement—checklist of items that should be included in reports of observational studies

	Item No	Recommendation	Our research
**Title and abstract**	1	(*a*) Indicate the study's design with a commonly used term in the title or the abstract	P1
		(*b*) Provide in the abstract an informative and balanced summary of what was done and what was found	P2
**Introduction**			
Background/rationale	2	Explain the scientific background and rationale for the investigation being reported	P4-5
Objectives	3	State specific objectives, including any prespecified hypotheses	P
**Methods**			
Study design	4	Present key elements of study design early in the paper	P5-10
Setting	5	Describe the setting, locations, and relevant dates, including periods of recruitment, exposure, follow-up, and data collection	P6-7
Participants	6	(*a*) *Cohort study*—Give the eligibility criteria, and the sources and methods of selection of participants. Describe methods of follow-up	-
		Case-control study—Give the eligibility criteria, and the sources and methods of case ascertainment and control selection. Give the rationale for the choice of cases and controls	_
		Cross-sectional study—Give the eligibility criteria, and the sources and methods of selection of participants	P7
		(*b*) *Cohort study*—For matched studies, give matching criteria and number of exposed and unexposed	_
		Case-control study—For matched studies, give matching criteria and the number of controls per case	_
Variables	7	Clearly define all outcomes, exposures, predictors, potential confounders, and effect modifiers. Give diagnostic criteria, if applicable	P9-10
Data sources/ measurement	8*	For each variable of interest, give sources of data and details of methods of assessment (measurement). Describe comparability of assessment methods if there is more than one group	P5-6
Bias	9	Describe any efforts to address potential sources of bias	P9
Study size	10	Explain how the study size was arrived at	P6-7
Quantitative variables	11	Explain how quantitative variables were handled in the analyses. If applicable, describe which groupings were chosen and why	P8-10
Statistical methods	12	(*a*) Describe all statistical methods, including those used to control for confounding	P8-10
		(*b*) Describe any methods used to examine subgroups and interactions	P8-10
		(*c*) Explain how missing data were addressed	-
		(*d*) *Cohort study*—If applicable, explain how loss to follow-up was addressed	_
		Case-control study—If applicable, explain how matching of cases and controls was addressed	_
		Cross-sectional study—If applicable, describe analytical methods	P8-10

For peer review only

For peer review only - http://bmjopen.bmj.com/site/about/guidelines.xhtml

taking account of sampling strategy	
(*e*) Describe any sensitivity analyses	_

Continued on next page

For peer review only

For peer review only - http://bmjopen.bmj.com/site/about/guidelines.xhtml

Results			Our research
Participants	13*	(a) Report numbers of individuals at each stage of study—eg numbers potentially eligible, examined for eligibility, confirmed eligible, included in the study, completing follow-up, and analysed	-
		(b) Give reasons for non-participation at each stage	-
		(c) Consider use of a flow diagram	-
Descriptive data	14*	(a) Give characteristics of study participants (eg demographic, clinical, social) and information on exposures and potential confounders	P10-11
		(b) Indicate number of participants with missing data for each variable of interest	-
		(c) *Cohort study*—Summarise follow-up time (eg, average and total amount)	-
Outcome data	15*	*Cohort study*—Report numbers of outcome events or summary measures over time	-
		Case-control study—Report numbers in each exposure category, or summary measures of exposure	-
		Cross-sectional study—Report numbers of outcome events or summary measures	P10
Main results	16	(*a*) Give unadjusted estimates and, if applicable, confounder-adjusted estimates and their precision (eg, 95% confidence interval). Make clear which confounders were adjusted for and why they were included	P10-P12
		(*b*) Report category boundaries when continuous variables were categorized	-
		(*c*) If relevant, consider translating estimates of relative risk into absolute risk for a meaningful time period	-
Other analyses	17	Report other analyses done—eg analyses of subgroups and interactions, and sensitivity analyses	P12
**Discussion**			
Key results	18	Summarise key results with reference to study objectives	P12-17
Limitations	19	Discuss limitations of the study, taking into account sources of potential bias or imprecision. Discuss both direction and magnitude of any potential bias	P17
Interpretation	20	Give a cautious overall interpretation of results considering objectives, limitations, multiplicity of analyses, results from similar studies, and other relevant evidence	P12-17
Generalisability	21	Discuss the generalisability (external validity) of the study results	P17
**Other information**			
Funding	22	Give the source of funding and the role of the funders for the present study and, if applicable, for the original study on which the present article is based	P18

*Give information separately for cases and controls in case-control studies and, if applicable, for exposed and unexposed groups in cohort and cross-sectional studies.

Note: An Explanation and Elaboration article discusses each checklist item and gives methodological background and published examples of transparent reporting. The STROBE checklist is best used in conjunction with this article (freely available on the Web sites of PLoS Medicine at http://www.plosmedicine.org/, Annals of Internal Medicine at http://www.annals.org/, and Epidemiology at http://www.epidem.com/). Information on the STROBE Initiative is available at www.strobe-statement.org.

For peer review only

For peer review only - http://bmjopen.bmj.com/site/about/guidelines.xhtml
